# Synthesis and preliminary cytotoxicity evaluation of water soluble pentacyclic triterpenoid phosphonates

**DOI:** 10.1038/s41598-024-76816-w

**Published:** 2024-11-14

**Authors:** Jevgeņija Lugiņina, Vladislavs Kroškins, Rihards Lācis, Elza Fedorovska, Öznur Demir, Arita Dubnika, Dagnija Loca, Māris Turks

**Affiliations:** 1 Institute of Chemistry and Chemical Technology, Faculty of Natural Sciences and Technology, Riga Technical University, 3 P.Valdena Street, Riga, LV-1048 Latvia; 2 Institute of Biomaterials and Bioengineering, Faculty of Natural Sciences and Technology, Riga Technical University, 3 Pulka Street, Riga, LV-1048 Latvia; 3 Baltic Biomaterials Centre of Excellence, Headquarters at Riga Technical University, Riga, Latvia

**Keywords:** Pentacyclic triterpenoids, Phosphonates, Cytotoxicity, Aqueous solubility, Bone cancer, Natural product synthesis

## Abstract

Synthesis, solubility and cytotoxicity evaluation of anionic phosphonates derived from betulin, betulinic acid, oleanolic acid and ursolic acid is reported. Phosphonate moieties were successfully installed at terpenoid C28 by carboxylic acid deprotonation/alkylation sequence using (dimethoxyphosphoryl)methyl trifluoromethanesulfonate as alkylation reagent. Also, betulin-derived and ether-linked bis-phosphonate is obtained and characterized. After demethylation in the presence of TMSI the resulting phosphonic acids were transformed into their disodium salts. All target compounds display excellent water solubility, which was determined by qNMR in D_2_O. Cytotoxicity tests were performed in different concentrations of each compound (10–50 µM) against human osteosarcoma cell line MG-63 and osteoblast precursor cell line MC3T3-E1. Improved aqueous solubility and low cytotoxicity profile of the newly designed triterpenoid phosphonates reveal high potential for various medicinal chemistry and pharmacological applications in the future.

**Supplementary Information:**

The online version contains supplementary material available at 10.1038/s41598-024-76816-w.

## Introduction

People have used various plant-derived products as remedies for illnesses since prehistoric times. Also, in the modern era, approximately a quarter of drugs are inspired by or derived from natural products. This is particularly pronounced in anticancer and anti-infective areas, where during the past four decades, more than half of drugs are either natural products or natural product derivatives, mimics of natural products, or compounds bearing natural product pharmacophore^1^. The use of naturally abundant agents can help to reduce toxic and side effects due to low toxicity profile in normal cells^2^. Enhanced safety and cost-effectivity promote natural products to great multitarget drug candidates^3^.

Pentacyclic triterpenoids (PCTs) belong to a widespread family of natural isoprene-derived secondary metabolites, which exhibit a broad spectrum of biological properties^4–8^. PCTs can be classified into three major groups: lupane (betulin, betulinic acid, and lupeol), oleanane (oleanolic acid, maslinic acid, erythrodiol, and β-amyrin) and ursane (ursolic acid, uvaol, and α-amyrin)^9^. The ubiquity of PCTs in nature, renewability and their facile isolation process have resulted in numerous studies that have explored potential therapeutic applications of these terpenoids. Among them, the most promising PCTs applications are in the anticancer and antiviral domains^10–16^. Emerging application fields of PCTs and their semi-synthetic derivatives include the search for antibacterial^8,17^ and antifungal agents^18,19^, and compounds that can treat diabetes^20,21^ and inflammatory conditions^22,23^.

However, the development of PCTs drugs is often jeopardized by their physicochemical properties that are characteristic of natural plant compounds – poor solubility in physiological media and low bioavailability^24–29^.The latter can be explained by the high lipophilicity of the steroidal core^30^, which leads to extremely low aqueous solubility. For example, the solubility of betulinic acid in distilled water is 21 ng/mL and 40.1 µg/mL at pH 11.4 in sodium phosphate solution^31^. Improvement of aqueous solubility is a possible option to overcome this limitation^32^, and it can be achieved by chemical modifications of the terpenoid structure^33–35^.One of the approaches is to decorate the lipophilic carbon skeleton either with heteroatoms^36–38^ or with polar ionogenic groups^39^.

**Fig. 1:**
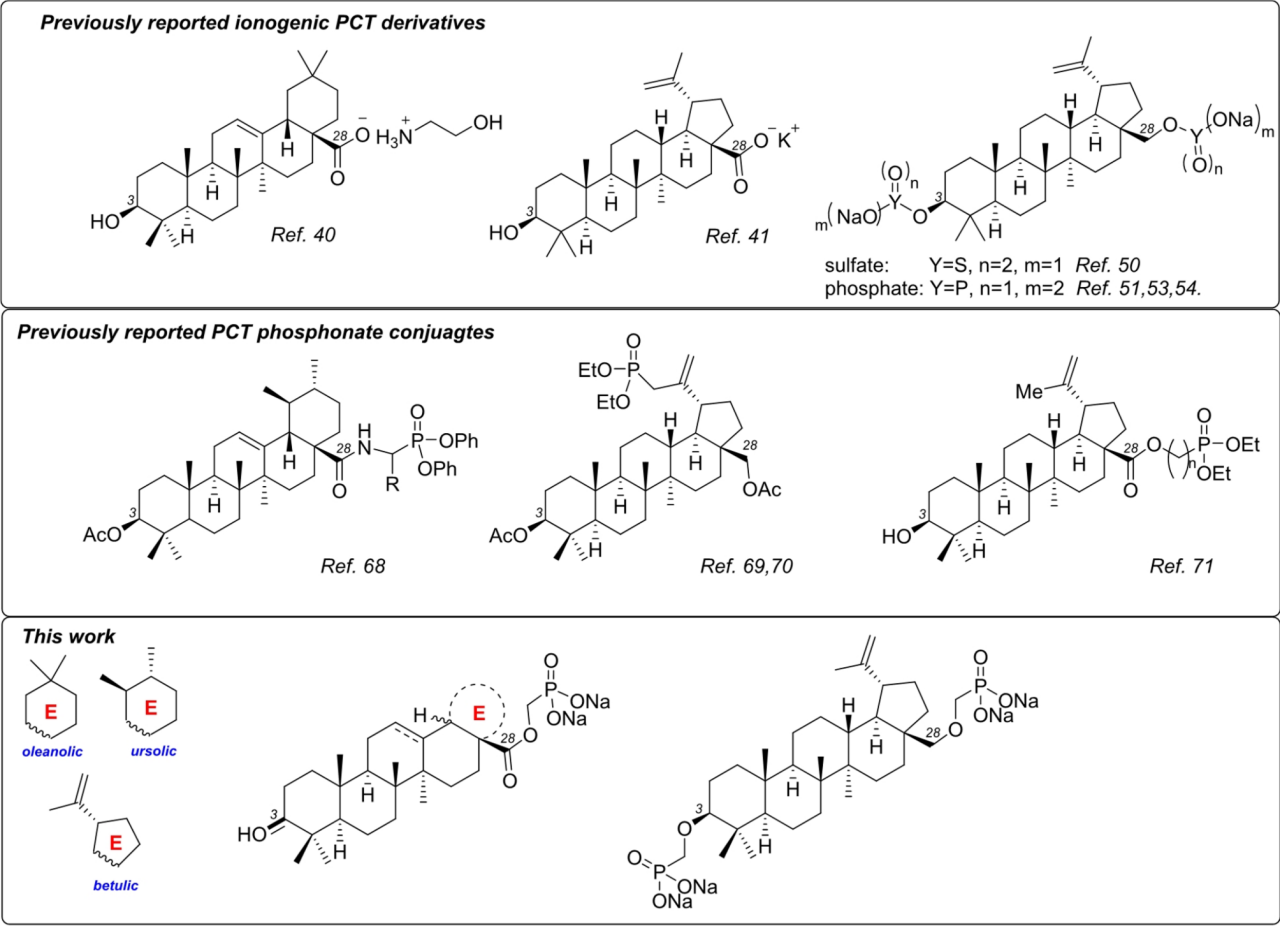
Previously reported ionogenic PCTs, PCT-derived phosphonate conjugates and the original target molecules.

PCTs such as betulinic, ursolic and oleanolic acids possess intrinsic polar ionogenic carboxylate group at C(28), which can be easily transformed into different anionic salts by treatment with alkali and quaternary ammonia hydroxides (Fig. 1). As an example, choline oleanolate displayed the best solubility (81.7 µg/mL) in a simulated gastric juice (aqueous solution of NaCl and sodium dodecyl sulfate, which was adjusted to pH 1.2 by HCl solution)^40,41^. However, solubility studies of potassium and sodium PCT carboxylates did not show satisfactory results, due to the formation of colloids at concentrations above 0.02 mg/g, which significantly complicated the solubility determination.

On the other hand, various semi-synthetic cationic PCT conjugates were reported during the past decade. Various ammonium^42,43^, guanidinium^44^ and imidazolium^45^ moieties possessing diverse counterions were attached to PCTs cores through different type and size linkers. Similarly, C(2), C(28) and C(30) PCT triphenylphosphonium salts have been synthesized and biologically evaluated^46,47^. Unfortunately, solubility data of these cationic PCTs were not reported.

Speaking about alternative semi-synthetic anionic PCT derivatives, sulfate^48–50^ and phosphate^51–60^ groups have been added to the PCTs cores by corresponding sulfation or phosphorylation of C(3)-OH or/and C(28)-OH groups. PCT-derived mono and diphosphates provide a wide range of biological applications. However, detailed aqueous solubility studies thereof are not available. Even if *O*-phosphorylation and *O*-sulfatation of terpenoids may improve their biological activity properties by making structural and spatial changes^61^, the use of such modified compounds can be hampered by low hydrolytic stability^62,63^.

The latter issue can be overcome by replacing the phosphates with isosteric and isoelectronic, yet more stable phosphonates. This approach has made phosphonate derivatives a prominent class of biologically active compounds that have been developed particularly well in the area of antiviral nucleotide drugs^64–67^. Phosphonates as phosphate mimics have also been studied in the triterpenoid series, but the reported examples are quite scarce. Thus, phosphonate moieties have been attached to the PCT core by an amide bond^68^ or C-C bond (Fig. 1)^69,70^. To the best of our knowledge, there is only one example of PCT-phosphonate connected by C(28) carboxylic ester^71^. Nevertheless, the conversion of the reported phosphonates to phosphonic acids or their salts is still unexplored, and the water solubility of such ionogenic species has not been described.

Herein, we report the design and synthesis of novel pentacyclic triterpenoid – phosphonate conjugates of type C(17)-COO-CH_2_-*P* and C(3/28)-O-CH_2_-*P*, where the phosphonate moiety is attached to the terpenoid core by the simplest possible methylene linker and chemically bound by ester or ether moiety (Fig. 1). It is important to note, that the PCT’s C(28) esters are hydrolytically stable and can resist basic and acidic treatment^72–74^. Thus, we demonstrate with the present report that such phosphonate esters and phosphonate sodium salts are straightforward to achieve and the latter are very soluble in aqueous solutions, which is often a delicate task to achieve in the field of triterpenoid chemistry.

## Results and discussion

### Synthesis

Initially, we speculated that the incorporation of methylene phosphonate moiety can be achieved through the ester bond formation between the triterpenic acid C28 carboxylate group and dialkyl (hydroxymethyl) phosphonate via activation of carboxylic acid. For this purpose, we have prepared a series of 3-oxo PCT carboxylic acids **2a**-**c** starting from commercially available betulin **1a**, oleanolic acid **1b** and ursolic acid **1c**. 3-Oxo triterpenic acids **2a-c** were successfully converted into corresponding acyl chlorides by SOCl_2_ treatment, but further reaction with dimethyl (hydroxymethyl)phosphonate was found to be inefficient, due to low conversion of the chloride into the desired product. Following screening of the reaction conditions did not bring the desired result, and the best yield that we reached was 30%. The weak reactivity of triterpenic acid chlorides can be explained by the steric hindrance at C(28) position. Some sources demonstrate that PCT acid halides are capable of reacting with amines^75^ and phenols^76^, but insufficient nucleophilicity of alcohols, together with the electron withdrawing effect of phosphonate moiety, makes it even more complicated.

Therefore, we decided to switch reactivity and explore a possible carboxylate alkylation reaction^77^, in which the reaction site is one atom further from the C(17) quaternary center.

**Fig. 2:**
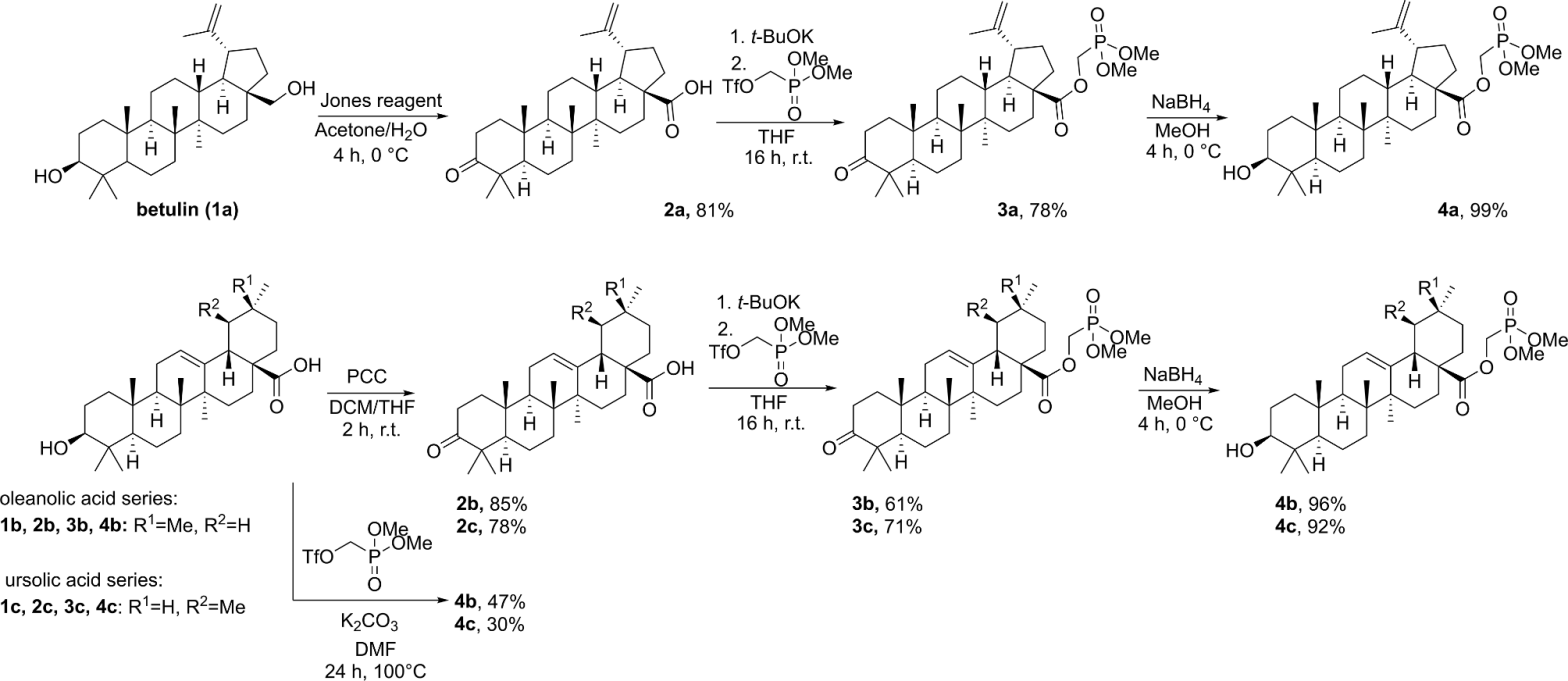
Synthetic route for the preparation of phosphonic esters **4a**-**c**.

We discovered that 3-oxo triterpenic acids **2a**-**c** underwent rapid deprotonation followed by alkylation with (dimethoxyphosphoryl)methyl trifluoromethanesulfonate in the presence of *t*-BuOK in anhydrous THF (Fig. 2). The used triflate is readily available from the previously mentioned alcohol^78^. The desired esters **3a**-**c** were obtained in good yields. Next, 3-hydroxy triterpenic phosphonates **4a-c** were obtained by diastereoselective reduction of C(3) ketones. A similar approach using (dimethoxyphosphoryl)methyl trifluoromethanesulfonate in a combination with 3-hydroxy triterpenic acids **1b**,** c** provided a direct access to compounds **4b**,** c** (process **1b**,** c** → **4b**,** c** Fig. 2), yet with a lower yield due to side reactions that implied laborious purification of the products. For the transformation **1b**,** c** → **4b**,** c** K_2_CO_3_ was used as a weaker base to achieve a better selectivity between C(17)-COOH and C(3)-OH alkylation, but this required a solvent change to DMF for a better solubility if the base. The latter protocol resulted also in transesterification between C(17)-COOH and phosphonic acid methyl ester moiety of the alkylation reagent yielding C(17)-COOMe side product accompanied by TfOCH_2_P(O)(OH)(OMe), the separation of which required additional reverse phase chromatography on C18-silica. Therefore, the developed sequence **2** → **3** → **4** is optimal, as it provides clean transformations and ensures access to both C(3)-OH and C(3) = O series of triterpenoids.

**Fig. 3:**
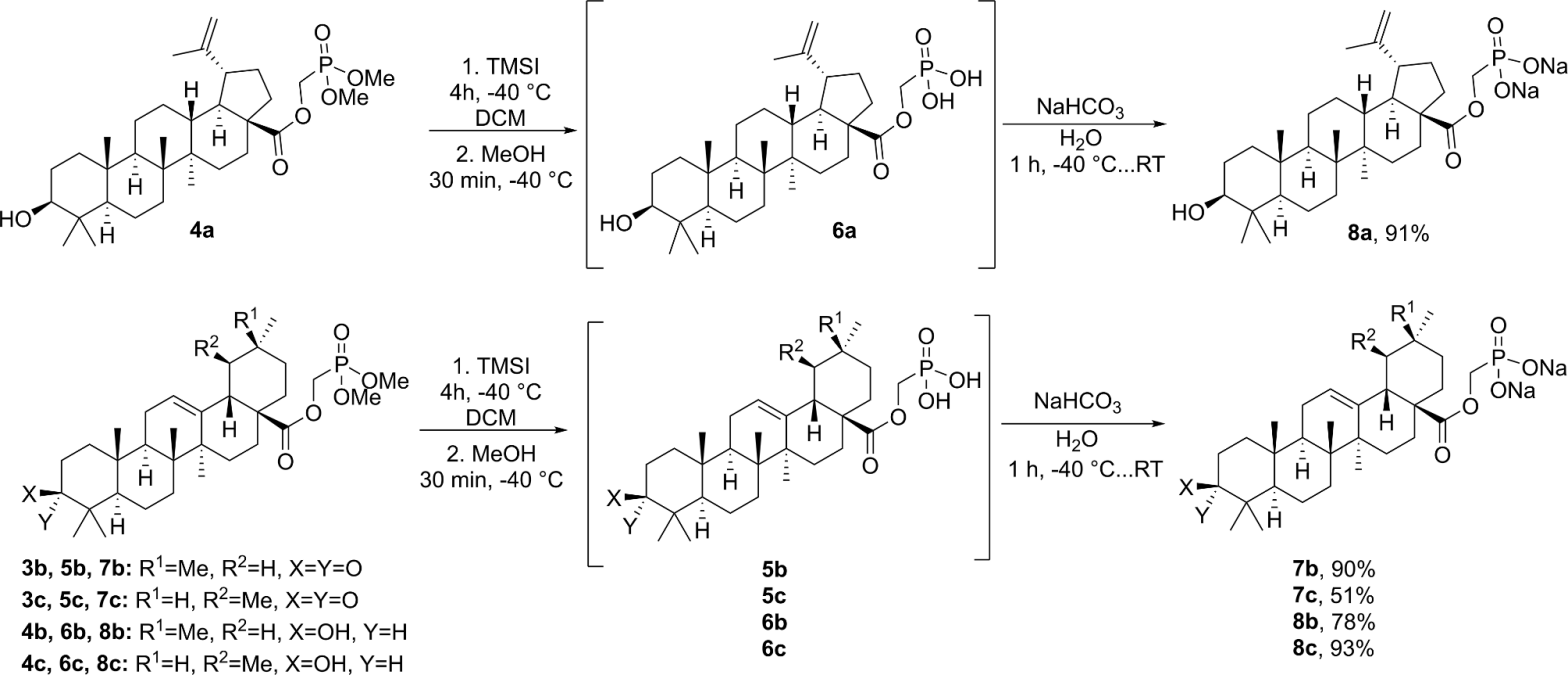
Synthesis of triterpenic acid derived sodium phosphonates **7b**,** c** and **8a-c**.

With the whole series of the desired phosphonates in hand, next we examined the transformation of phosphonates into sodium phosphonates 7**a**-**c** and **8a**-**c** employing TMSI assisted demethylation followed by the conversion of phosphonic acids **5a**-**c** and **6a**-**c** into their salts (Table 1; Fig. 3). Starting with the betulonic acid derived phosphonate **3a** (Table 1), we found that the demethylation must be carried out at -40 °C. At higher temperatures betulinic acid olefin moiety underwent cationic rearrangements^79^ and cleavage of the previously installed ester was observed (Table 1, entries 1, 2). We supposed that the methanolysis of the intermediate *O*-TMS-phosphonates could also be accomplished at a lowered temperature, but the undesired side product was still formed during the evaporation of the solvent (Table 1, entry 3). Neutralization of HI before warming up the reaction mixture was found to be crucial (Table 1, entry 4). Therefore, sodium bicarbonate aqueous solution must be added subsequently at a lowered temperature, ensuring neutralization of HI and formation of sodium salt **7a**.

**Table 1:** Demethylation studies of 3-oxo-PCT phosphonate **3a**.


Entry	Conditions	Yield of **7a** (%)	Yield of **5a’** (%)
1	1. TMSI (5 equiv.), 2 h, RT 2. MeOH, 30 min, 0 °C	0	83
2	1. TMSI (3 equiv.), 1 h, − 40 °C → 0 °C 2. MeOH, 10 min, 0 °C	43	25
3	1. TMSI (3 equiv.), 4 h, − 40 °C 2. MeOH, 10 min, -78 °C	50	16
**4**	1. **TMSI (3 equiv.)**,** 4 h**,** − 40 °C** 2. **MeOH**,** 30 min**,** − 40 °C** 3. **NaHCO**_**3**_**(4 equiv.)**,** 1 h**,** − 40 °C → RT**	**97**	0

The developed demethylation conditions for the transformation **3a** → **7a** were successfully applied on all other compound series consisting of betulinic acid derivative **4a** with free C(3)-OH group, 3-oxo-series of oleanolic and ursolic acid-derived phosphonates **3b**,** c** and their corresponding C(3)-OH derivatives **4b**,** c** (Fig. 3). The target products **7a-c** and **8a-c** were obtained in good to excellent yields and isolated by a simple precipitation/centrifugation approach.

**Fig. 4:**
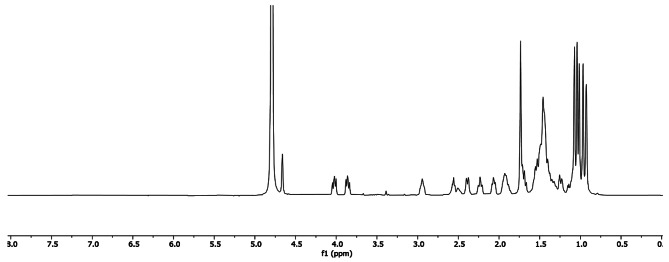
^1^H-NMR (500 MHz, D_2_O) spectrum of 3-oxo-oleanolic acid-derived sodium phosphonate **7b** (5 mg/mL).

As expected, the obtained products **7a-c** and **8a-c** were hydrolytically very stable and an eventual cleavage of the carboxylate ester bond was not observed even after heating under two different basic conditions: (1) 60 °C in 1.5 M NaOH/MeOH solution for 6 h; (2) 100 °C in the presence of 4 equiv. NaOH in H_2_O for 24 h. The obtained ionogenic derivatives revealed excellent water solubility, which can be nicely demonstrated by the acquisition of their ^1^H NMR spectra in D_2_O (Fig. 4).

**Fig. 5:**
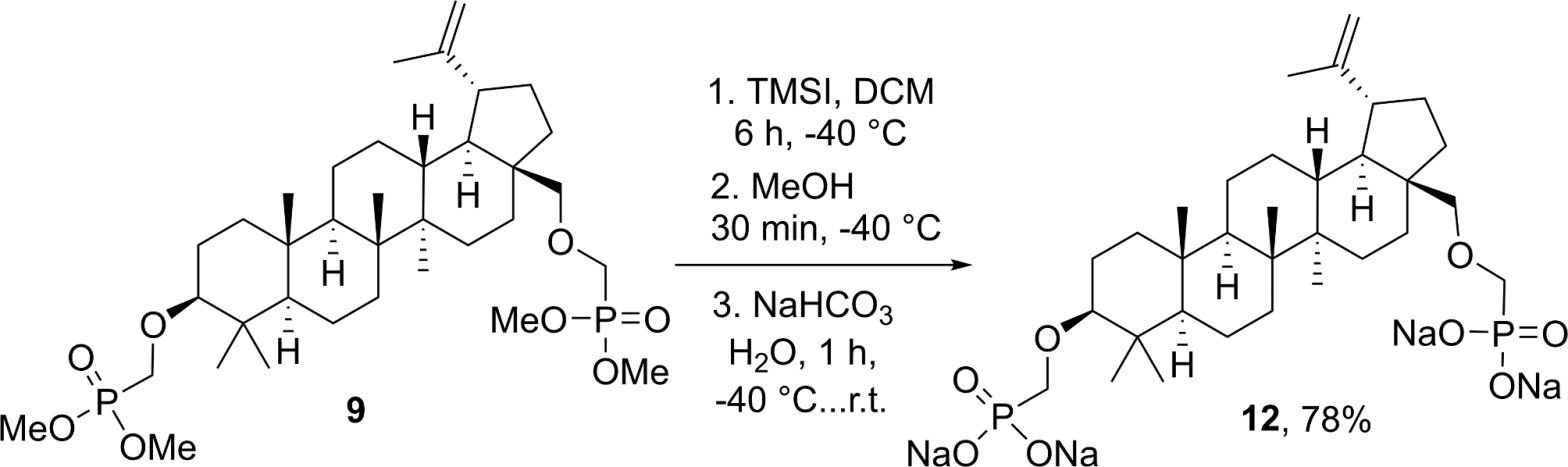
Synthesis of betulin-derived tetrasodium bis-phosphonate **12**.

Next, we investigated the installation of phosphonate moiety via an ether bond (Table 2). Starting with betulin, the most abundant natural PCT-3,28-diol, we examined a one-pot double alkylation possibility involving both hydroxyl groups. Application of such strong bases as NaH, *t*-BuOK, *n*-BuLi and MeMgBr in combination with (dimethoxyphosphoryl)methyl trifluoromethanesulfonate or tosylate were found to be ineffective (Table 2, entries 1–6). On many occasions we detected the degradation of alkylating reagent. To our delight, we have finally found that combining triflate alkylation reagent (2.2 equiv.) and betulin Li-dialkoxide arising from LDA (lithium diisopropylamide) (2.1 equiv.) provided the expected product **9** in 29% isolated yield (Table 2, entry 9). The desired product **9** was accompanied by the C(28)-*O*-monoalkylation product **10** and C(28)-*O*-phosphonylation product **11** in the **9**:**10**:**11** ratio 51:20:29 (by NMR). The latter arises from the alkoxide attack on the phosphorous center due to the presence of two competing electrophilic reaction centers in (dimethoxyphosphoryl)methyl trifluoromethanesulfonate. The obtained tetramethyl bis-phosphonate **9** was converted to tetrasodium salt (78%) using the previously developed TMSI conditions (Fig. 5).

**Table 2:** Method development for the synthesis of tetramethyl bis-phosphonate **9**.


Entry	Reaction conditions					^1^H NMR ratio of products, % (isolated yield, %)			
	Base (equiv.)	X (equiv.)	Solvent	Temperature (°C)	Time (h)	**1**	**9**	**10**	**11**
1.	NaH (2.0)	OTf (2.2)	THF	0 °C → RT	3	100	0	0	0
2.	*t*-BuOK (2.1)	OTf (2.2)	THF	RT → 50 °C	3	100	0	0	0
3.	MeMgCl (2.2)	OTs (4.0)	THF	RT	36	0	0	0	0
4.	MeMgCl (2.2)	OTs (4.0)	THF	40 °C	16	0	0	0	0
5.	MeMgCl (2.2)	OTs (4.0)	CyHex	75 °C	16	100	0	0	0
6.	*n*-BuLi (2.1)	OTf (2.5)	THF	-78 °C → RT	3	0	10	41	49
7.	LDA (2.4)	OTf (2.6)	THF	-78 °C – RT	3	0	6	72	22
8.	LDA (2.4)	OTf (2.2)	THF	-78 °C → RT	3	0	40(14)	42	18
**9.**	**LDA (2.1)**	**OTf (2.2)**	**THF**	**-78 °C → RT**	**3**	**0**	**51(29)**	**20**	**29**
10.	LDA (2.1)	OTf (2.1)	THF	-78 °C → -30 °C	3	61	9	24	6
11.	LDA (2.1)	Br (2.1)	THF	-78 °C → RT	3	0	0	0	0

## Compound solubility and cytotoxicity evaluation

The obtained PCT-derived sodium phosphonates were subjected to water solubility tests (Table 3). For this purpose, we used a quantitative ^1^H-NMR approach in D_2_O, using potassium hydrogen phthalate as an external standard (for experimental details see Supporting Information). The presence of the basic forms of phosphonates^80^ was ensured by the careful addition of NaOD, maintaining pH 8.0–8.5 during the quantification, which is 2–3 units higher than the pKa of phosphonic acid disalt^81^. As expected, our newly designed phosphonates **7a-c**, **8a-c** and **12** exhibited excellent aqueous solubility in a range from 3 to 26 mg/mL (pH 8.0–8.5). This is by at least two to three orders of magnitude higher than the reported solubilities of the parent natural triterpenic acids. For example, aqueous solubility of oleanolic and betulinic acids are < 0.1 µg/mL at neutral pH and can be increased to 42.1 µg/mL for betulinic acid and 99.5 µg/mL for oleanolic acid at pH 11.8^31^. Also, parent ursolic acid exhibits a similarly low aqueous solubility^29^, which can be enhanced to some extent by various modern drug delivery systems^26–28^. Indeed, phosphonic acids are more acidic and easier ionizable than carboxylic acids. This ionic character helps to increase the aqueous solubility as exemplified by here described compounds. On the other hand, a direct numerical comparison of the aqueous solubility of compounds **7a-c**, **8a-c** and **12** with the reported triterpenoid phosphate/phosphonate analogs^51–60^ is burdened as most of the previous reports describe the obtained enhanced solubility in a qualitative manner.

**Table 3:** Aqueous solubility data of PCT-derived sodium phosphonates at pH 8.0…8.5.

Compound number	Structural formula	Aqueous solubility (mg/mL) in D_2_O at pH 8.0…8.5
**7a**		11
**7b**		8
**7c**		26
**8a**		3
**8b**		7
**8c**		7
**12**		3

All target compounds were studied to determine their cytotoxic activity at various concentrations of each compound (10–50 µM) against human-derived osteosarcoma cell line MG-63 (ATCC, CRL-1427) and mouse-derived preosteoblast cell line MC3T3-E1 (ATCC, CRL-2593) (Table 4 and Figures S1-S4 in the Supporting Information). For the comparison, naturally occurring betulinic, oleanolic (**1b**) and ursolic (**1c**) acids as well as their 3-oxo analogs **2a-c** and doxorubicin, were also subjected to the cytotoxicity tests. As expected, the designed water-soluble PCT-derived phosphonates and the natural triterpenic acids, including their 3-oxo-analogs, were harmless to the MC3T3-E1 cells. As an interesting observation and exception should be mentioned the concentration-dependent cell viability drop of MC3T3-E1 cells in the presence of oleanonic acid (0.49 ± 0.12 relative metabolic activity at 50 µM of **2b**). To a lesser extent, ursonic acid affected the metabolic activity of MC3T3-E1 cells (0.72 ± 0.09 relative metabolic activity at 50 µM of **2c**).

Nevertheless, in the presence of oleanonic acid-derived phosphonate **8b** the MG-63 cell line revealed somewhat lower metabolic activity (0.73 ± 0.05 relative metabolic activity at 50 µM of **8b**) than in the presence of its parental oleanonic acid (1.03 ± 0.18 for **2b**). It is interesting to note that ursolic acid (**1c**) and ursonic acid (**2c**) showed a cytotoxic effect towards MG-63 cell line in the cell viability tests (0.28 ± 0.04 and 0.67 ± 0.04 relative metabolic activity at 50 µM of **1c** and **2c**, respectively).

**Table 4:** Cell metabolic activity data in the presence of the title phosphonates and their comparison to naturally occuring PCTs and doxorubicin.

Compound	Relative metabolic activity of MG-63 cells at given concentrations of PCTs			Relative metabolic activity of MC3T3-E1 cells at given concentrations of PCTs		
	50 µM	25 µM	10 µM	50 µM	25 µM	10 µM
Betulinic acid	1.12 ± 0.08	0.91 ± 0.14	0.74 ± 0.16	0.97 ± 0.07	0.56 ± 0.04	0.76 ± 0.07
Betulonic acid **(2a)**	0.88 ± 0.08	0.96 ± 0.04	1.14 ± 0.02	1.22 ± 0.01	1.70 ± 0.05	1.39 ± 0.06
**7a**	1.09 ± 0.04	1.30 ± 0.06	1.10 ± 0.04	1.13 ± 0.03	1.27 ± 0.05	1.41 ± 0.01
**8a**	1.59 ± 0.17	1.24 ± 0.25	1.58 ± 0.05	1.01 ± 0.05	1.19 ± 0.04	1.08 ± 0.05
Oleanolic acid (**1b**)	1.29 ± 0.11	1.58 ± 0.13	0.77 ± 0.14	0.96 ± 0.05	0.97 ± 0.08	1.04 ± 0.07
Oleanonic acid (**2b**)	1.03 ± 0.18	1.60 ± 0.03	1.51 ± 0.08	0.49 ± 0.12	0.99 ± 0.05	1.10 ± 0.01
**7b**	1.19 ± 0.05	1.32 ± 0.05	1.13 ± 0.08	1.04 ± 0.05	1.50 ± 0.04	1.31 ± 0.08
**8b**	0.73 ± 0.05	0.76 ± 0.04	0.84 ± 0.04	0.86 ± 0.08	0.97 ± 0.04	1.00 ± 0.03
Ursolic acid (**1c**)	0.28 ± 0.04	0.64 ± 0.03	0.66 ± 0.03	0.99 ± 0.08	0.83 ± 0.05	1.06 ± 0.05
Ursonic acid (**2c**)	0.67 ± 0.04	0.67 ± 0.04	0.76 ± 0.08	0.72 ± 0.09	1.07 ± 0.07	0.74 ± 0.02
**7c**	1.12 ± 0.03	0.98 ± 0.04	0.96 ± 0.04	0.94 ± 0.05	0.90 ± 0.03	0.91 ± 0.06
**8c**	1.26 ± 0.08	0.91 ± 0.05	1.22 ± 0.09	0.95 ± 0.03	0.89 ± 0.04	0.93 ± 0.09
**12**	0.94 ± 0.04	0.89 ± 0.05	0.97 ± 0.08	0.92 ± 0.04	0.93 ± 0.06	0.90 ± 0.07
Doxorubicin	0.16 ± 0.02	0.19 ± 0.01	0.41 ± 0.02	0.53 ± 0.03	0.60 ± 0.03	0.72 ± 0.07
Positive control	1.42 ± 0.03			1.10 ± 0.10		
Negative control	0.16 ± 0.08			0.28 ± 0.12		

In summary, we have developed a practical synthetic approach for introduction of the simplest phosphonate moiety, containing only single methylene group, into the PCT core via an ester and an ether bond. The corresponding TMSI induced phosphonate demethylation, which provided the target sodium phosphonates, was optimized for a preparative application avoiding acid promoted cationic rearrangements of the triterpenic core. Phosphonate disodium salts were obtained and characterized for the pentacyclic triterpenoids in the betulinic, oleanolic and ursolic acid series, including their 3-oxo-derivatives. All target compounds possess excellent aqueous solubility (3–26 mg/mL at pH 8.0–8.5), which was properly quantified by qNMR. Thus, this report stands out with the sample ^1^H NMR spectra of pentacyclic triterpenoid derivatives acquired in D_2_O. The preliminary cytotoxicity evaluation of the target products revealed that the obtained PCT-derived sodium phosphonates do not possess significant cytotoxicity profile towards normal cells. This fact provides a promising possibility for future studies of these and similar phosphonate derivatives in those biological activity domains that require high selectivity between normal mammalian cells and external factors, such as viral, bacterial and fungal pathogens. Due to their non-toxic nature the title compounds classify also for further studies in the field of antidiabetic and anti-inflammatory agents.

## Methods

### Synthesis: general information

Solvents for the reactions were dried over standard drying agents and freshly distilled prior to use. All purchased chemicals (Fluka, Aldrich) were used as received. All reactions were followed by TLC on E. Merck Kieselgel 60 F_254_ and visualized by using UV lamp. Column chromatography was performed on silica gel (60 Å, 40–63 μm, ROCC). Flash column chromatography was performed on a Büchi Sepacore system (Büchi-Labortechnik GmbH, Essen, Germany) with a Büchi Control Unit C-620, an UV detector Büchi UV photometer C-635, Büchi fraction collector C-660 and two Pump Modules C-605. ^1^H and ^13^C NMR spectra were recorded on a Bruker 300 and 500 MHz, in CDCl_3_ or [D_4_]MeOD at 25 °C. Chemical shifts (δ) values are reported in ppm. The residual solvent peaks are used as internal reference (CDCl_3_) 7.26 ppm, [D_4_]MeOD 3.31 ppm for ^1^H NMR, CDCl_3_ 77.16 ppm, [D_4_]MeOD 49.00 ppm for ^13^C NMR), s (singlet), d (doublet), t (triplet), q (quartet), m (multiplet); *J* in hertz. ^1^H and ^13^C NMR peaks were assigned by analysis of multidimensional NMR (COSY, HSQC, HMBC). For ^31^P NMR calibration, Ph_3_P was used as external reference (-6.00 ppm in MeOD_*d4*_) in a coaxially inserted tube. High-resolution massspectra (ESI) were performed on Agilent 1290 Infinity series UPLC connected to Agilent 6230 TOF mass spectrometer (calibration at m/z 121.050873 and m/z 922.009798). Optical rotation was measured at 20 °C on Anton Paar MCP 500 polarimeter (1-cm cell) using multi-wavelength analysis (589 nm, 546 nm, 436 nm, 405 nm, 365 nm).

## General procedure I for the synthesis of 3-oxo-triterpenic acid esters, process 2a-c → 3a-c

To solution of 3-oxo-triterpenic acid **2a-c** (500 mg, 1.099 mmol, 1 eq.) in anhydrous THF (5 mL) *t*BuOK (185 mg, 1.649 mmol, 1 eq.) is added portion wise at -5 °C. The resulting reaction mixture is stirred in ambient temperature for 30 min, then warmed up to room temperature and stirred for additional 60 min. The obtained mixture is re-cooled to -5 °C and a solution of previously prepared (dimethoxyphosphoryl)methyl trifluoromethanesulfonate (629 mg, 2.198 mmol, 2 eq.) in anhydrous THF (5 mL) is added dropwise. Then the resulting reaction mixture is warmed up to room temperature and stirred for 12 h. The reaction mixture is evaporated to dryness, redissolved in EtOAc (70 mL) and washed with brine (3 × 10 mL). Separated organic layer is dried over anhydrous Na_2_SO_4_. After filtration, the filtrate is concentrated *in vacuo* and purified by silica column chromatography (Hexanes-EtOAc 9:1 → 1:1) to yield carboxylic ester as a white amorphous solid: **3a** (78%, 495 mg); **3b** (61%, 387 mg), **3c** (71%, 450 mg).

### 3-oxo-(17 S)-17-(((dimethoxyphosphoryl)methoxy)carbonyl)-28-norlup-20(29)-ene 3a

^1^H NMR (500 MHz, CDCl_3_) δ 4.73 (s, 1H, H_a_-C(29)), 4.60 (s, 1H, H_b_-C(29)), 4.48 (dd, ^2^*J* = 14.6, 8.3 Hz, 1H, H_a_-C(28’)), 4.39 (dd, ^2^*J* = 14.6, 8.2 Hz, 1H, H_b_-C(28’)), 3.81 (d, ^3^*J* = 10.9, 6 H, (OMe)_2_), 2.98 (td, ^3^*J* = 11.1, 4.8 Hz, 1H, H_−_C(19)), 2.48 (ddd, ^2^*J* = 15.5 Hz, ^3^*J* = 9.8, 7.5 Hz, 1H, H_a_-C(2)), 2.39 (ddd, ^2^*J* = 15.5 Hz, ^3^*J =* 7.6, 4.4 Hz, 1H, H_b_-C(2)), 2.31–2.19 (m, 2H, H_a_-C(16), H-C(13)), 1.96–1.82 (m, 4 H, H_a_-C(15), H_a_-C(21), H_a_-C(22), H_a_-C(1)), 1.76–1.69 (m, 1H, H_a_-C(12)), 1.68 (s, 3H, H_3_-C(30)), 1.62 (t, ^3^*J* = 11.4 Hz, 1H, H-C(18)), 1.54–1.34 (m, 9 H, H_2_-C(6), H_a_-C(11), H_2_-C(7), H_b_-C(21), H_b_-C(1), H-C(9), H_b_-C(16)), 1.34–1.14 (m, 4 H, H_b_-C(11), H_b_-C(15), H_b_-C(22), H-C(5)), 1.06 (s, 3H, H_3_-C(23)), 1.06–1.01 (m, 1H, H_b_-C(12)), 1.01 (s, 3H, H_3_-C(24)), 0.97 (s, 3H, H_3_-C(27)), 0.96 (s, 3H, H_3_-C(26)), 0.92 (s, 3H, H_3_-C(25)). ^13^C NMR (126 MHz, CDCl_3_) δ 218.25 (C3), 174.91 (d, ^3^*J* = 6.4 Hz, (C28)), 150.34 (C20), 109.96 (C29), 56.90 (C17), 55.18 (d, ^1^*J* = 167.4 Hz), 55.10 (C5), 53.20 (d, ^2^*J =* 6.2 Hz, MeO), 53.18 (d, ^2^*J* = 6.2 Hz, MeO), 50.04 (C9), 49.49 (C18), 47.46 (C4), 46.90 (C19), 42.61 (C14), 40.77 (C10), 39.77 (C1), 38.44 (C13), 37.04 (C21), 37.00 (C10), 34.26 (C2), 33.75 (C7), 32.03 (C16), 30.53 (C22), 29.65 (C15), 26.74 (C23), 25.65 (C12), 21.54 (C11), 21.15 (C24), 19.76 (C30), 19.47 (C6), 16.10 (C25), 15.85 (C26), 14.74 (C27). ^31^P NMR (121 MHz, CDCl_3_) δ 21.77. HRMS: [C_33_H_53_O_6_P + H^+^] 577.3653; found 577.3626 (4.7 ppm). $\:[{\alpha]}_{D}^{20}=\:+0.19$; $\:[{\alpha]}_{546}^{20}=\:+0.23$; [$\:{\alpha]}_{436}^{20}=\:+0.50$; $\:[{\alpha]}_{405}^{20}=\:+0.66$; $\:{[\alpha]}_{365}^{20}=\:+1.02$ (c = 1.00, MeOH).

## 3-oxo-(17 S)-17-(((dimethoxyphosphoryl)methoxy)carbonyl)-28-norolean-12(13)-ene 3b

^1^H NMR (500 MHz, CDCl_3_): δ 5.32 (t, ^3^*J* = 3.6 Hz, 1H, H-C(12)), 4.42 (dd, ^2^*J* = 14.0, 8.6 Hz, 1H, H_a_-C(28`)), 4.32 (dd, ^2^*J* = 14.0, 8.6 Hz, 1H, H_b_-C(28`)), 3.81 (d, ^2^*J* = 10.8 Hz, 6 H, (MeO)_2_), 2.88 (dd, ^3^*J* = 14.0, 4.3 Hz, 1H, H-C(18)), 2.55 (ddd, ^2^*J* = 15.9 Hz, ^3^*J* = 11.2, 7.2 Hz, 1H, H_a_-C(2)), 2.36 (ddd, ^2^*J* = 15.9 Hz, ^3^*J* = 6.8, 3.6 Hz, 1H, H_b_-C(2)), 2.01 (dt, ^2^*J* = 14.0 Hz, ^3^*J* = 4.3 Hz, 1H, H_a_-C(16)), 2.00–1.90 (m, 2H, H_2_-C(11)), 1.88 (ddd, ^2^*J* = 13.2 Hz, ^3^*J* = 7, 3.6 Hz, 1H, H_a_-C(1)), 1.72 (dt, 1H, ^2^*J* = 13.6 Hz, ^3^*J* = 4.1 Hz, H_a_-C(22)), 1.69–1.53 (m, 5 H, H_b_-C(16), H_a_-C(19), H-C(9), H_a_-C(7), H_a_-C(15)), 1.52–1.47 (m, 3H, H_2_-C(6), H_b_-C(7)), 1.40 (dt, ^2^*J* = 12.3 Hz, ^3^*J* = 6.0 Hz, 1H, H_b_-C(1)), 1.35–1.28 (m, 4 H, H_b_-C(22), H_a_-C(21), H-C(5), H_b_-C(6)), 1.26–1.08 (m, 3H, H_b_-C(21), H_b_-C(19), H_b_-C(15)), 1.14 (s, 3H, H_3_-C(27)), 1.08 (s, 3H, H_3_-C(23)), 1.04 (s, 6 H, H_3_-C(24), H_3_-C(25)), 0.92 (s, 3H, H_3_-C(29)), 0.90 (s, 3H, H_3_-C(30)), 0.78 (s, 3H, H_3_-C(26)). ^13^C NMR (126 MHz, CDCl_3_): δ 217.90 (C(3)), 176.77 (d, ^3^*J*= 8.0 Hz, (C28)), 143.55 (C13), 122.65 (C12), 55.82 (d, ^1^*J* = 168.7 Hz, (C28`)), 55.47 (C5), 53.24 (d, ^2^*J*= 6.4 Hz, (MeO)_2_), 47.60 (C4), 47.25 (C17), 46.98 (C9), 45.90 (C19), 41.96 (C14), 41.54 (C18), 39.40 (C8), 39.29 (C1), 36.89 (C10), 34.31 (C2), 33.91 (C21), 33.16 (C30), 32.37 (C7), 32.32 (C22), 30.81 (C20), 27.75 (C15), 26.56 (C23), 25.87 (C27), 23.66 (C29), 23.65 (C11), 23.19 (C16), 21.63 (C24), 19.72 (C6), 16.95 (C26), 15.18 (C25). ^31^P NMR (121 MHz, CDCl_3_) δ 21.74. HRMS: [C_33_H_53_O_6_P + H^+^] 577.3653; found 577.3626 (4.7 ppm). $\:[{\alpha]}_{D}^{20}=\:+0.66$; $\:[{\alpha]}_{546}^{20}=\:+0.79$; [$\:{[\alpha]}_{436}^{20}=\:+1.38$; $\:[{\alpha]}_{405}^{20}=\:+1.70$; $\:{[\alpha]}_{365}^{20}=\:+2.28$ (c = 1.00, MeOH).

## 3-oxo-(17 S)-17-(((dimethoxyphosphoryl)methoxy)carbonyl)-28-norurs-12(13)-ene 3c

^1^H NMR (500 MHz, CDCl_3_) δ 5.29 (d, ^3^*J* = 3.8 Hz, 1H, H-C(12)), 4.33 (d, ^2^*J* = 8.5 Hz, 2H, H_2_-C(28’)), 3.80 (d, ^3^*J* = 10.8 Hz, 6 H, (OMe)_2_), 2.55 (ddd, ^2^*J* = 16.0 Hz, ^3^*J* = 10.8, 7.5 Hz, 1H, H_a_-C(2)), 2.37 (ddd, ^2^*J* = 16.0 Hz, ^3^*J* = 6.9, 3.6 Hz, 1H, H_b_-C(2)), 2.25 (d, ^3^*J* = 11.3 Hz, 1H, H-C(18)), 2.04 (td, ^2^*J* = 14.4, ^3^*J* = 6.5 Hz, 1H, H_a_-C(16)), 1.99–1.92 (m, 2H, H_2_-C(11)), 1.90 (ddd, ^2^*J* = 12.0 Hz, ^3^*J* = 7.4, 3.6 Hz, 1H, H_a_-C(1)), 1.80–1.68 (m, 3H, H_b_-C(16), H_a_-C(15), H_a_-C(21)), 1.65–1.55 (m, 2H, H_b_-C(21), H-C(9)), 1.55–1.39 (m, 5 H, H_2_-C(6), H_a_-C(15), H_a_-C(7), H_b_-C(1)), 1.39–1.23 (m, 4 H, H_b_-C(22), H_b_-C(7), H-C(19, H-C(5)), 1.17–1.11 (m, 1H, H_b_-C(15)), 1.09 (s, 3H, H_3_-C(27)), 1.08 (s, 3H, H_3_-C(28)), 1.04 (s, 6 H, H_3_-C(24), H_3_-C(25)), 1.04–0.98 (m, 1H, H-C(20)), 0.94 (d, ^3^*J* = 6.3 Hz, 3H, H_3_-C(30)), 0.86 (d, ^3^*J* = 6.4 Hz, 3H, H_3_-C(29)), 0.79 (s, 3H, H_3_-C(26)). ^13^C NMR (126 MHz, CDCl_3_) δ 217.95 (C3), 176.62 (d, ^3^*J* = 8.2 Hz, (C28)), 138.02 (C13), 125.83 (C12), 55.81 (d, ^1^*J* = 168.9 Hz, (C28’)), 55.13 (C5), 53,24 (d, ^2^*J* = 6.1 Hz, (OMe)), 53.21 (d, ^2^*J* = 6.1 Hz, (OMe)), 53.07 (C18), 48.64 (C17), 47.55 (C4), 46.86 (C9), 42.28 (C14), 39.60 (C8), 39.44 (C1), 39.17 (C19), 38.91 (C20), 36.79 (C10), 36.58 (C21), 34.31 (C2), 32.65 (C7), 30.68 (C22), 28.08 (C15), 26.67 (C23), 24.32 (C16), 23.59 (C27), 23.58 (C11), 21.63 (C24), 21.24 (C30), 19.71 (C6), 17.12 (C29), 17.05 (C26), 15.37 (C25). ^31^P NMR (121 MHz, CDCl_3_) δ 21.85. HRMS: [C_33_H_53_O_6_P + H^+^] 577.3653; found 577.3623 (5.2 ppm). $\:[{\alpha]}_{D}^{20}=\:+0.50$; $\:[{\alpha]}_{546}^{20}=\:+0.58$; [$\:{[\alpha]}_{436}^{20}=\:+0.97$; $\:[{\alpha]}_{405}^{20}=\:+1.23$; $\:{[\alpha]}_{365}^{20}=\:+1.62$ (c = 1.00, MeOH).

### General procedure II for synthesis of 3-hydroxy-triterpenic acid esters, process 3a-c → 4a-c

To solution of 3-oxo-triterpenoic acid ester **3a-c** (200 mg, 0.347, 1 eq.) in MeOH (4 mL) NaBH_4_ (53 mg, 1.388 mmol, 4 eq.) is added portion wise at 0 °C. The resulting reaction mixture is stirred at ambient temperature for 5 h. Then the reaction mixture is quenched by NH_4_Cl saturated aqueous solution (2 mL), evaporated to dryness, redissolved in EtOAc (25 mL) and washed with brine (3 × 10 mL). The combined organic layer is dried over anhydrous Na_2_SO_4_. After filtration, the filtrate is concentrated *in vacuo* and purified by silica column chromatography (Hexanes-EtOAc 9:1 → 1:1) to yield product as a white amorphous solid: **4a** (99%, 198 mg); **4b** (96%, 193 mg); **4c** (92%, 185 mg).

### (17 S)-17-(((dimethoxyphosphoryl)methoxy)carbonyl)-3β-hydroxy-28-norlup-20(29)-ene 4a

^1^H NMR (500 MHz, CDCl_3_) δ 6.02 (bs, 1H, OH), 4.73 (s, 1H, H_a_-C(29)), 4.61 (s, 1H, H_b_-C(29)), 4.48 (dd, ^2^*J* = 14.7, 8.2 Hz, 1H, H_a_-C(28’), 4.39 (dd, ^2^*J* = 14.7, 8.2 Hz, 1H, H_b_-C(28’), 3.81 (d, ^3^*J* = 10.8 Hz, 6 H, (OMe)_2_), 3.18 (dd, ^3^*J* = 11.4, 4.7 Hz, 1H, H-C(3)), 2.98 (td, ^3^*J* = 11.2, 4.7 Hz, 1H, H-C(19)), 2.30–2.34 (m, 1H, H_a_-C(16)), 2.24–2.15 (m, 1H, H-C(13)), 1.98–1.83 (m, 2H, H_a_-C(21), H_a_-C(22)), 1.74–1.31 (m, 17 H, H_a_-C(15), H_3_-C(C30), H_a_-C(12), H-C(18), H_2_-C(6), H_a_-C(11), H_2_-C(7), H_b_-C(21), H_a_-C(1), H-C(9), H_b_-C(16), H_2_-C(2)), 1.31–1.13 (m, 3H, H_b_-C(11), H_b_-C(15), H_b_-C(22)), 1.08–0.99 (m, 1H, H_b_-C(12)), 0.96 (s, 6 H, H_3_-C(27), H_3_-C(26)), 0.92 (s, 3H, H_3_-C(23)), 0.90–0.85 (m, 1H, H_b_-C(1)), 0.82 (s, 3H, H_3_-C(25)), 0.75 (s, 3H, H_3_-C(24)), 0.68 (d, ^3^*J* = 9,4 Hz, 1H, H-C(5)). ^13^C NMR (126 MHz, CDCl_3_) δ 174.97 (d, ^3^*J* = 6.4 Hz, (C28)), 150.46 (C20), 109.91 (C29), 79.12 (C3), 56.96 (C17), 55.0 (C5), 55.17 (d, ^1^*J* = 167.4 Hz, C(28’)), 53.20 (d, ^2^*J* = 6.1 Hz, (MeO)), 53.17 (d, ^2^*J* = 6.1 Hz, (MeO)), 50.70 (C9), 49.58 (C18), 46.96 (C19), 42.58 (C14), 40.85 (C8), 39.01 (C4), 38.88 (C1), 38.39 (C13), 37.35 (10), 37.05 (C21), 34.48 (C7), 32.12 (C16), 30.57 (C22), 29.70 (C15), 28.13 (C23), 27.55 (C2), 25.67 (C12), 21.03 (C11), 19.49 (C30), 18.44 (C6), 16.30 (C25), 16.07 (C26), 15.50 (C24), 14.85 (C27). ^31^P NMR (121 MHz, CDCl_3_) δ 21.88. HRMS: [C_33_H_55_O_6_P + H^+^] 579.3809; found 579.3782 (4.7 ppm). $\:[{\alpha]}_{D}^{20}=\:-0.01$; $\:[{\alpha]}_{546}^{20}=\:-0.01$; [$\:{\alpha]}_{436}^{20}=\:+0.01$; $\:[{\alpha]}_{405}^{20}=\:+0.02$; $\:{[\alpha]}_{365}^{20}=\:+0.02$ (c = 1.00, MeOH).

### (17 S)-17-(((dimethoxyphosphoryl)methoxy)carbonyl)-3β-hydroxy − 28-norolean-12(13)-ene 4b

^1^H NMR (500 MHz, CDCl_3_) δ 5.30 (t, ^3^*J* = 3.6 Hz, 1H, H-C(13)), 4.41 (dd, ^2^*J* = 14.6, 8.7 Hz, 1H, H_a_-C(28’)), 4.31 (dd, ^2^*J* = 14.6, 8.3 Hz, 1H, H_b_-C(28’)), 3.80 (d, ^3^*J* = 10.9 Hz, 6 H, (OMe)_2_), 3.25–3.17 (m, 1H, H-C(3)), 2.90–2.81 (m, 1H, H-C(18)), 2.07–1.95 (m, 1H, H_a_-C(16)), 1.91–1.85 (m, 2H, H_2_-C(11)), 1.77–1.16 (m, 16 H, H_2_-C(6), H_b_-C(16), H_2_-C(2), H_a_-C(15), H_2_-C(22), H_2_-C(7), H_2_-C(21), H-C(9), H_a_-C(1), H_2_-C(19)), 1.13 (s, 3H, H_3_-C(27)), 1.09 (d, ^2^*J* = 14.0 Hz, 1H, H_b_-C(15)), 0.98 (s, 3H, H_3_-C(23)), 0.98–0.92 (m, 1H, H_b_-C(1)), 0.92 (s, 3H, H_3_-C(29)), 0.90 (s, 6 H, H_3_-C(30), H_3_-C(25)), 0.78 (s, 3H, H_3_-C(24)), 0.77–0.72 (m, 1H, H-C(5)), 0.72 (s, 3H, H_3_-C(26)). ^13^C NMR (126 MHz, CDCl_3_) δ 176.81 (d, ^3^*J* = 7.8 Hz, (C28)), 143.49 (C12), 122.89 (C12), 79.16 (C3), 55.92 (d, ^1^*J* = 169.5 Hz, (C28’)), 55.13 (C5)), 53.23 (d, ^2^*J* = 6.3 Hz, (OMe)), 53.23 (d, ^2^*J* = 6.3 Hz, (OMe)), 47.73 (C9), 47.24 (C17), 45.97 (C19), 41.85 (C14), 41.48 (C18), 39.43 (C8), 38.90 (C4), 38.60 (C1), 37.18 (C10), 33.93 (C21), 33.18 (C30), 32.86 (C7), 32.38 (C22), 30.81 (C20), 28.25 (C23), 27.77 (C15), 27.34 (C2), 25.99 (C27), 23.69 (C29), 23.57 (C11), 23.21 (C16), 18.48 (C6), 17.02 (C26), 15.72 (C24), 15.48 (C25). ^31^P NMR (121 MHz, CDCl_3_) δ 21.77. HRMS: [C_33_H_55_O_6_P + NH_4_^+^] 596.4075; found 596.4042 (5.5 ppm). $\:[{\alpha]}_{D}^{20}=\:+0.38$; $\:[{\alpha]}_{546}^{20}=\:+0.46$; [$\:[{\alpha]}_{436}^{20}=\:+0.79$; $\:[{\alpha]}_{405}^{20}=\:+0.97$; $\:[{\alpha]}_{365}^{20}=\:+1.27$ (c = 1.00, MeOH).

### (17 S)-17-(((dimethoxyphosphoryl)methoxy)carbonyl)-3β-hydroxy-28-norurs-12(13)-ene 4c

^1^H NMR (500 MHz, CDCl_3_) δ 5.26 (bs, 1H, H-C(13)), 4.33 (d, ^2^*J* = 8.4 Hz, 2H, H_2_-C(28’)), 3.80 (d, ^3^*J* = 10.8 Hz, 6 H, (OMe)_2_), 3.21 (dd, ^3^*J* = 11.3, 4.6 Hz, 1H, H-C(3)), 2.23 (d, ^3^*J* = 11.3 Hz, 1H, H-C(18)), 2.10–1.99 (m, 1H, H_a_-C(16)), 1.95–1.84 (m, 2H, H_2_-C(11)), 1.79–1.68 (m, 3H, H_b_-C(16), H_a_-C(15), H_a_-C(21)), 1.67–1.22 (m, 12 H, H_b_-C(21), H_a_-C(1), H_2_-C(2), H_2_-C(6), H_2_-C(7), H_2_-C(22), H-C(9), H-C(19)), 1.13–1.08 (m, 1H, H_b_-C(15)), 1.08 (s, 3H, H_3_-C(27)), 1.04–0.99 (m, 1H, H-C(20)), 0.99 (s, 3H, H_3_-C(23)), 0.99–0.94 (m, 1H, H_b_-C(1)), 0.94 (d, ^3^*J* = 6,3 Hz, 3H, H_3_-C(30)), 0.92 (s, 3H, H_3_-C(25)), 0.86 (d, ^3^*J* = 6.6 Hz, 3H, H_3_-C(29)), 0.78 (s, 3H, H_3_-C(24)), 0.73 (s, 3H, H_3_-C(26)), 0.73–0.69 (m, 1H, H-C(5)). ^13^C NMR (126 MHz, CDCl_3_) δ 176.66 (d, ^3^*J* = 8.3 Hz, (C28)), 137.93 (C12), 126.08 (C13), 79.18 (C3), 55.81 (d, ^1^*J* = 168.9 Hz, (C28’)), 55.36 (C5), 53.25 (d, ^2^*J* = 6.4 Hz, (MeO)), 53.25 (d, ^2^*J* = 6.4 Hz, (MeO)), 53.03 (C18), 48.63 (C17), 47.67 (C9), 42.18 (C14), 39.65 (C8), 39.19 (C19), 38.95 (C20), 38.90 (C4), 38.77 (C1), 37.11 (C10), 36.64 (C21), 33.15 (C7), 30.72 (C22), 28.28 (C23), 28.12 (C25), 27.37 (C2), 24.37 (C16), 23.70 (C27), 23.45 (C11), 21.28 (C30), 18.46 (C6), 17.13 (C26), 17.09 (C29), 15.76 (C24), 15.62 (C25). ^31^P NMR (121 MHz, CDCl_3_) δ 21.77. HRMS: [C_33_H_55_O_6_P + H^+^] 579.3809; found 579.3781 (4.8 ppm). $\:[{\alpha]}_{D}^{20}=\:+0.36$; $\:[{\alpha]}_{546}^{20}=\:+0.43$; [$\:[{\alpha]}_{436}^{20}=\:+0.76$; $\:[{\alpha]}_{405}^{20}=\:+0.92$; $\:[{\alpha]}_{365}^{20}=\:+1.20$ (c = 1.00, MeOH).

### **General procedure III for demethylation of phosphonic esters**,** processes 3a-b → 7a-c and 4a-c → 8a-c**

To solution of 3-oxo-triterpenic acid ester **3a-c** or 3-hydroxy-triterpenoic acid ester **4a-c** (0.4 mmol, 1 eq.) in anhydrous DCM (5 mL) TMSI (171 µL 1.2 mmol, 3 eq.) is added dropwise at -40 °C and the resulting reaction mixture is stirred at -40 °C for 5 h. Then MeOH (2.5 mL) is added dropwise at -40 °C. The obtained mixture is stirred for additional 30 min at the same temperature and solution of NaHCO_3_ (101 mg, 1.2 mmol, 3 eq.) in H_2_O (4 mL) is added dropwise at -40 °C. The resulting reaction mixture is warmed up to room temperature and the organic solvents are evaporated *in vacuo*. The obtained aqueous suspension is centrifuged and the supernatant is removed and discarded. The precipitate is re-suspended in deionized water (1 mL) and the centrifugation – supernatant removal procedure is repeated additional two times (in total: washing with water 3 × 1mL). The obtained precipitated is dried at ambient temperature *in vacuo*: **7a** ( 97%, 230 mg); **7b** (90%, 214 mg); **7c** (51%, 121 mg); **8a** (91%, 217 mg); **8b** (78%, 186 mg); **8c** (93%, 222 mg).

### Sodium (3-oxo-(17R)-17-28-norlup-20(29)-en)-2-oxoethyl-phosphonate 7a

^1^H NMR (500 MHz, MeOD_*d4*_) δ 4.72 (s, 1H, H_a_-C(29)), 4.58 (s, 1H, H_b_-C(29)), 4.16 (dd, ^2^*J* = 13.1, 8.7 Hz, 1H, H_a_-C(28’)), 3.90 (dd, ^2^*J* = 13.1, 8.4 Hz, 1H, H_b_-C(29)), 3.02 (td, ^3^*J* = 11.2, 4.4 Hz, 1H, H-C(19)), 2.58–2.36 (m, 4 H, H_2_-C(2), H-C(13), H_a_-C(16)), 2.24 (dd, ^3^*J* = 11.7, 8.1 Hz, 1H, H_a_-C(21)), 2.00–1.86 (m, 2H, H_a_-C(21), H_b_-C(22)), 1.76 (d, ^3^*J* = 13.0 Hz, 1H, H_a_-C(12)), 1.69 (s, 3H, H_3_-C(30)), 1.63 (t, ^3^*J* = 11.3 Hz, 1H, H-C(18)), 1.57–1.27 (m, 13H, H_2_-C(6), H_2_-C(11), H_2_-C(7), H_a_-C(15), H_b_-C(22), H_b_-C(16), H_b_-C(1), H-C(9), H_b_-C(21), H-C(5)), 1.23–1.15 (m, 1H, H_b_-C(22)), 1.13–1.06 (m, 1H, H_b_-C(12)), 1.06 (s, 3H, H_3_-C(23)), 1.02 (s, 3H, H_3_-C(24)), 1.01 (s, 3H, H_3_-C(27)), 1.00 (s, 3H, H_3_-C(26)), 0.95 (s, 3H, H_3_-C(25)). ^13^C NMR (126 MHz, MeOD_*d4*_) δ 221.02 (C3), 176.99 (d, ^3^*J* = 8.4 Hz, (C28)), 151.94 (C20), 110.22 (C29), 59.92 (d, ^1^*J* = 162.6 Hz, (C28’)), 57.99 (C17), 56.11 (C5), 51.23 (C9), 50.68 (C18), 48.31 (C19), 43.61 (C14), 41.87 (C8), 40.70 (C4), 39.60 (C11), 38.06 (C13), 37.79 (C10), 35.05 (C21), 34.74 (C7), 34.72 (C16), 32.92 (C2), 31.61 (C22), 30.88 (C15), 27.17 (C23), 26.89 (C12), 22.62 (C11), 21.43 (C24), 20.76 (C6), 19.55 (C30), 16.54 (C26), 16.35 (C25), 15.03 (C27). ^31^P NMR (121 MHz, MeOD_*d4*_) δ 14.20. HRMS: [C_31_H_49_O_6_P-H^+^] 547.3194; found 547.3198 (0.7 ppm). $\:[{\alpha]}_{D}^{20}=\:+0.16$; $\:[{\alpha]}_{546}^{20}=\:+0.19$; [$\:[{\alpha]}_{436}^{20}=\:+0.38$; $\:[{\alpha]}_{405}^{20}=\:+0.51$; $\:[{\alpha]}_{365}^{20}=\:+0.80$ (c = 1.00, MeOH).

### Sodium (3-oxo-(17R)-17-28-norolean-12(13)-en)-2-oxoethyl-phosphonate 7b

^1^H NMR (500 MHz, MeOD_*d4*_) δ 5.30 (d, ^3^*J* = 3.7 Hz, 1H, H-C(13)), 4.21–4.09 (m, 2H, H_2_-C(28’)), 2.92 (dd, ^3^*J* = 14.1, 4.5 Hz, 1H, H-C(18)), 2.57 (ddd, ^2^*J* = 16.1 Hz, ^3^*J* = 10.8, 7.4 Hz, 1H, H_a_-C(2)), 2.37 (ddd, ^2^*J* = 16.1 Hz, ^3^*J* = 7.1, 3.6 Hz, 1H, H_b_-C(2)), 2.09–1.86 (m, 4 H, H_a_-C(16), H_2_-C(11), H_a_-C(1)), 1.84–1.60 (m, 6 H, H_b_-C(16), H_a_-C(15), H_2_-C(22), H_a_-C(19), H-C(9)), 1.59–1.32 (m, 7 H, H_2_-C(6), H_2_-C(7), H_a_-C(21), H_b_-C(1), H-C(5)), 1.21 (d, ^2^*J* = 13.2 Hz, 1H, H_b_-C(21)), 1.18 (s, 3H, H_3_-C(27)), 1.16–1.09 (m, 2H, H_b_-C(15), H_b_-C(19)), 1.08 (s, 6 H, H_3_-C(23), H_3_-C(25)), 1.05 (s, 3H, H_3_-C(24)), 0.95 (s, 3H, H_3_-C(29)), 0.91 (s, 3H, H_3_-C(30)), 0.83 (s, 3H, H_3_-C(25)). ^13^C NMR (126 MHz, MeOD_*d4*_) δ 220.52 (C3), 180.23 (d, ^3^*J* = 9.0 Hz, (C28)), 145.38 (C12), 123.35 (C13), 60.43 (d, ^1^*J* = 162.8 Hz, (C28’)), 56.60 (C5), 48.83 (C4), 48.53 (C17), 48.26 (C9), 47.32 (C19), 42.98 (C14), 42.93 (C18), 40.56 (C8), 40.27 (C1), 37.92 (C10), 35.11 (C2), 35.00 (C21), 33.64 (C30), 33.38 (C7), 33.10 (C22), 31.58 (C20), 29.14 (C15), 26.96 (C23), 26.30 (C27), 24.59 (C11), 24.15 (C29), 23.78 (C16), 21.90 (C24), 20.73 (C6), 17.54 (C26), 15.53 (C25). ^31^P NMR (121 MHz, MeOD_*d4*_) δ 11.54. HRMS: [C_31_H_49_O_6_P-H^+^] 547.3194; found 547.3194 (0 ppm). $\:[{\alpha]}_{D}^{20}=\:+0.47$; $\:[{\alpha]}_{546}^{20}=\:+0.56$; [$\:[{\alpha]}_{436}^{20}=\:+0.98$; $\:[{\alpha]}_{405}^{20}=\:+1.22$; $\:[{\alpha]}_{365}^{20}=\:+1.65$ (c = 1.00, MeOH).

### Sodium (3-oxo-(17R)-17-28-norurs-12(13)-en)-2-oxoethyl-phosphonate 7c

^1^H NMR (500 MHz, MeOD_*d4*_) δ 5.30 (d, ^3^*J* = 3.7 Hz, 1H, H-C(12)), 4.08 (d, ^2^*J* = 13.2 Hz, 1H, H_a_-C(28’)), 4.00 (d, ^2^*J* = 13.2 Hz, 1H, H_b_-C(28’)), 2.57 (ddd, ^2^*J* = 16.0 Hz, ^3^*J* = 10.7, 7.4 Hz, 1H, H_a_-C(2)), 2.39 (ddd, ^2^*J* = 16.0 Hz, ^3^*J* = 7.1, 3.7 Hz, 1H, H_b_-C(2)), 2.32 (d, ^3^*J* = 11,3 Hz, 1H, H-C(18)), 2.10–1.90 (m, 5 H, H_2_-C(11), H_a_-C(16), H_a_-C(15), H_a_-C(1)), 1.90–1.81 (m, 2H, H_b_-C(16), H_a_-C(21)), 1.72 (dt, ^2^*J* = 13.8 Hz, ^3^*J* = 4.0 Hz, 1H, H_b_-C(21)), 1.67 (dd, ^3^*J* = 11.0, 5.7 Hz, 1H, H-C(9)), 1.62–1.44 (m, 5 H, H_2_-C(6), H_a_-C(22), H_a_-C(7), H_b_-C(1)), 1.44–1.28 (m, 4 H, H_b_-C(22), H_b_-C(7), H-C(5), H-C(19)), 1.12 (s, 3H, H_3_-C(27)), 1.12–1.08 (m, 1H, H_b_-C(15)), 1.08 (s, 6 H, H_3_-C(23), H_3_-C(25)), 1.05 (s, 3H, H_3_-C(24)), 1.03–0.99 (m, 1H, H-C(20)), 0.95 (d, ^3^*J* = 6.2 Hz, 3H, H_3_-C(30)), 0.89 (d, ^3^*J* = 6.4 Hz, 3H, H_3_-C(29)), 0.86 (s, 3H, H_3_-C(26)). ^13^C NMR (126 MHz, MeOD_*d4*_) δ 220.64 (C3), 179.92 (d, ^3^*J* = 8.8 Hz, (C28)), 139.68 (C13), 126.80 (C12), 62.93 (d, ^1^*J* = 153.5 Hz, (C28’)), 56.50 (C5), 54.32 (C18), 49.49 (C17), 48.15 (C9), 43.30 (C14), 40.80 (C8), 40.46 (C19), 40.41 (C1), 40.23 (C20), 37.82 (C10), 37.44 (C21), 35.14 (C7), 33.68 (C2), 31.84 (C22), 29.39 (C15), 27.07 (C23), 25.07 (C16), 24.51 (C11), 24.07 (C27), 21.92 (C24), 21.60 (C30), 20.72 (C6), 17.67 (C29), 17.63 (C26), 15.72 (C25). ^31^P NMR (121 MHz, MeOD_*d4*_) δ 11.53. HRMS: [C_31_H_49_O_6_P-H^+^] 547.3194; found 547.3195 (0.2 ppm). $\:[{\alpha]}_{D}^{20}=\:+0.49$; $\:[{\alpha]}_{546}^{20}=\:+0.58$; [$\:[{\alpha]}_{436}^{20}=\:+0.97$; $\:[{\alpha]}_{405}^{20}=\:+1.23$; $\:[{\alpha]}_{365}^{20}=\:+1.62$ (c = 1.00, MeOH).

### Sodium (3β-hydroxy-(17R)-17-28-norlup-20(29)-en)-2-oxoethyl-phosphonate 8a

^1^H NMR (500 MHz, MeOD_*d4*_) δ 4.62 (s, 1H, H_a_-C(29)), 4.48 (s, 1H, H_b_-C(29)), 4.08 (dd, ^2^*J* = 13.5, 8.9 Hz, 1H, H_a_-C(28’)), 3.95 (dd, ^2^*J* = 13.5, 8.9 Hz, 1H, H_b_-C(28’)), 3.02 (dd, ^3^*J* = 11.4, 4.6 Hz, 1H, H-C(3)), 2.93 (td, ^3^*J* = 11.3, 4.5 Hz, 1H, H-C(19)), 2.32 (d, ^2^*J* = 12.3 Hz, 1H, H_a_-C(16)), 2.27–2.19 (m, 1H, H-C(13)), 1.99 (dd, ^2^*J* = 12.1 Hz, ^3^*J* = 8.1 Hz, 1H, H_a_-C(21)), 1.88–1.80 (m, 1H, H_a_-C(22)), 1.66–1.57 (m, 1H, H_a_-C(12)), 1.59 (s, 3H, H_3_-C(30)), 1.57–1.11 (m, 15 H, H_2_-C(6), H_2_-C(11), H_2_-C(7), H_2_-C(2), H_b_-C(22), H_b_-C(21), H_b_-C(16), H_a_-C(1), H-C(9), H-C(18), H_a_-C(15)), 1.11–0.91 (m, 2H, H_b_-C(15), H_b_-C(12)), 0.89 (s, 3H, H_3_-C(27)), 0.87–0.82 (m, 7 H, H_3_-C(23), H-C(1), H_3_-C(26)), 0.76 (s, 3H, H_3_-C(25)), 0.65 (s, 3H, H_3_-C(24)), 0.63–0.57 (m, 1H, H-C(5)). ^13^C NMR (126 MHz, MeOD_*d4*_) δ 177.50 (d, ^3^*J* = 8.8 Hz, (C28)), 152.07 (C20), 110.11 (C29), 79.69 (C3), 61,13 (d, ^1^*J* = 159.5 Hz, (C28’)), 57.95 (C17), 56.90 (C5), 52.05 (C9), 50.81 (C18), 48.32 (C19), 43.52 (C14), 41.95 (C8), 40.11 (C4), 39.96 (C1), 39.42 (C13), 38.33 (C10), 37.83 (C21), 35.55 (C7), 32.99 (C16), 31.67 (C22), 30.95 (C15), 28.61 (C23), 28.05 (C2), 26.90 (C12), 22.08 (C11), 19.57 (C30), 19.45 (C6), 16.74 (C25), 16.62 (C26), 16.11 (C24), 15.11 (C27). ^31^P NMR (121 MHz, MeOD_*d4*_) δ 11.47. HRMS: [C_31_H_51_O_6_P-H^+^] 549.3350; found 549.3351 (0.2 ppm). $\:[{\alpha]}_{D}^{20}=\:-0.07$; $\:[{\alpha]}_{546}^{20}=\:-0.08$; [$\:[{\alpha]}_{436}^{20}=\:-0.10$; $\:[{\alpha]}_{405}^{20}=\:-0.10$; $\:[{\alpha]}_{365}^{20}=\:-0.11$ (c = 1.00, MeOH).

### Sodium (3β-hydroxy-(17R)-17-28-norurs-12(13)-en)-2-oxoethyl-phosphonate 8b

^1^H NMR (500 MHz, MeOD_*d4*_) δ 5.27 (d, ^3^*J* = 3.9 Hz, 1H, H-C(13)), 4.06 (dd, ^2^*J* = 10.3, 5.1 Hz, 1H, H_a_-C(28’)), 4.02 (dd, ^2^*J* = 10.3, 5.1 Hz, 1H, H_b_-C(28’)), 3.15 (dd, ^3^*J* = 11.4, 4.6 Hz, 1H, H, H-C(3)), 2.31 (d, ^3^*J* = 11.3 Hz, 1H, H-C(18)), 2.04 (td, ^2^*J* = 13.3 Hz, ^3^*J* = 4.3 Hz, 1H, H_a_-C(16)), 1.96–1.85 (m, 3H, H_2_-C(11), H_a_-C(15)), 1.84–1.73 (m, 2H, H_b_-C(16), H_a_-C(21)), 1.73–1.61 (m, 3H, H_a_-C(2), H_b_-C(21), H_a_-C(1)), 1.61–1.46 (m, 5 H, H_a_-C(6), H_b_-C(2), H_a_-C(22), H_a_-C(7), H-C(9)), 1.45–1.26 (m, 4 H, H_b_-C(6), H_b_-C(22), H_b_-C(7), H-C(19)), 1.11 (s, 3H, H_3_-C(27)), 1.07 (d, ^2^*J* = 13.4 Hz, 1H, H_b_-C(15)), 1.04–0.98 (m, 2H, H-C(20), H_b_-C(1)), 0.97 (s, 3H, H_3_-C(23)), 0.96 (d, ^3^*J* = 6.3 Hz, 3H, H_3_-C(30)), 0.95 (s, 3H, H_3_-C(25)), 0.89 (d, ^3^*J* = 6.4 Hz, 3H, H_3_-C(25)), 0.79 (s, 3H, H_3_-C(24)), 0.78 (s, 3H, H_3_-C(26)), 0.74 (d, ^3^*J* = 11.4 Hz, 1H, H-C(5)). ^13^C NMR (126 MHz, MeOD_*d4*_) δ 179.47 (d, ^3^*J* = 9.2 Hz, C(28)), 139.44 (C13), 127.13 (C12), 79.72 (C3), 61.63 (d, ^1^*J* = 158.8 Hz, C(28’)), 56.75 (C5), 54.19 (C18), 49.49 (C17), 49.03 (C9), 43.12 (C14), 40.83 (C8), 40.39 (C19), 40.21 (C20), 40.01 (C1), 39.84 (C4), 38.09 (C10), 37.51 (C21), 34.20 (C7), 31.77 (C22), 29.29 (C15), 28.76 (C23), 27.90 (C2), 25.09 (C16), 24.36 (C11), 24.18 (C27), 21.59 (C30), 19.47 (C6), 17.70 (C24), 17.62 (C29), 16.37 (C26), 16.03 (C25). ^31^P NMR (121 MHz, MeOD_*d4*_) δ 12.17. HRMS: [C_31_H_51_O_6_P-H^+^] 549.3350; found 549.3349 (0.2 ppm). $\:[{\alpha]}_{D}^{20}=\:+0.33$; $\:[{\alpha]}_{546}^{20}=\:+0.40$; [$\:[{\alpha]}_{436}^{20}=\:+0.71$; $\:[{\alpha]}_{405}^{20}=\:+0.88$; $\:[{\alpha]}_{365}^{20}=\:+1.12$ (c = 1.00, MeOH).

### Sodium (3β-hydroxy-(17R)-17-28-norolean-12(13)-en)-2-oxoethyl-phosphonate 8c

^1^H NMR (500 MHz, MeOD_*d4*_) δ 5.25 (t, ^3^*J* = 3.6 Hz, 1H, H-C(13)), 4.16 (dd, ^2^*J* = 13.2, 8.8 Hz, 1H, H_a_-C(28’)), 3.96 (dd, ^2^*J* = 13.3, 8.6 Hz, 1H, H_b_-C(28’)), 3.14 (dd, ^3^*J* = 11.4, 4.5 Hz, 1H, C(3)), 2.88 (dd, ^3^*J* = 14.2, 4.4 Hz, 1H, H-C(18)), 1.99 (dt, ^2^*J* = 13.8 Hz, ^3^*J* = 7,7 Hz, 1H, H_a_-C(16)), 1.96–1.79 (m, 3H, H_b_-C(16), H-C(11)), 1.79–1.27 (m, 13H, H_2_-C(6), H_2_-C(2), H_a_-C(15), H_2_-C(22), H_2_-C(7), H_a_-C(21), H_a_-C(1), H_a_-C(19), H-C(9)), 1.19 (d, ^2^*J* = 14.9 Hz, 1H, H_b_-C(21)), 1.15 (s, 3H, H_3_-C(27)), 1.13–1.00 (m, 2H, H_b_-C(19), H_b_-C(15)), 1.00–0.95 (m, 4 H, H_3_-C(23), H_b_-C(1)), 0.94 (s, 6 H, H_3_-C(29), H_3_-C(25)), 0.90 (s, 3H, H_3_-C(30)), 0.78 (s, 6 H, H_3_-C(26), H_3_-C(24)), 0.77–0.72 (m, 1H, H-C(5)). ^13^C NMR (126 MHz, MeOD_*d4*_) δ 180.03 (d, ^3^*J* = 8.9 Hz, C(C28)), 145.27 (C12), 123.59 (C13), 79.77 (C3), 62.76 (d, ^1^*J* = 154.7 Hz, (C28’)), 56.80 (C5), 49.17 (C9), 48.11 (C17), 47.34 (C19), 42.85 (C18), 40.60 (C14), 39.88 (C8), 39.85 (C1), 38.17 (C4), 38.17 (C10), 34.99 (C21), 33.94 (C7), 33.64 (C30), 33.17 (C22), 31.58 (C20), 29.11 (C15), 28.74 (C23), 27.88 (C2), 26.41 (C27), 24.53 (C11), 24.16 (C29), 23.80 (C16), 19.50 (C6), 17.67 (C26), 16.30 (C24), 15.91 (C25). ^31^P NMR (121 MHz, MeOD_*d4*_) δ 11.59. HRMS: [C_31_H_51_O_6_P-H^+^] 549.3350; found 549.3348 (0.4 ppm). $\:[{\alpha]}_{D}^{20}=\:+0.31$; $\:[{\alpha]}_{546}^{20}=\:+0.43$; [$\:[{\alpha]}_{436}^{20}=\:+0.76$; $\:[{\alpha]}_{405}^{20}=\:+0.91$; $\:[{\alpha]}_{365}^{20}=\:+1.21$ (c = 1.00, MeOH).

### Synthesis of a mixture of products 9, 10 and 11

To solution of betulin (**1a**) (500 mg, 1.131 mmol, 1 eq.) in anhydrous THF (5mL) freshly prepared 1 M LDA solution in THF (2.37 mL, 2.37 mmol, 2.1 eq.) is added dropwise at -78 °C. The resulting reaction mixture is warmed up to 0 °C and stirred at ambient temperature for 40 min. Then the solution of previously prepared (dimethoxyphosphoryl)methyl trifluoromethanesulfonate (712 mg, 2.49 mmol, 2.2 eq.) in anhydrous THF (3 mL) is added dropwise to the suspension of lithium alkoxide at 0 °C. The obtained reaction mixture is warmed up to room temperature and stirred for 3 h. Then the reaction mixture is quenched by MeOH (2 mL ), evaporated to dryness, redissolved in EtOAc (50 mL) and subsequently washed with H_2_O (30 mL) and brine (2 × 30 mL). The combined organic layer is dried over anhydrous Na_2_SO_4_. After filtration, the filtrate is concentrated *in vacuo* and purified by silica column chromatography (Hexanes-EtOAc 4:1–1:9) to yield bis-ether **9** as a white amorphous solid (29%, 226 mg). R*f* = 0.31 (100% EtOAc). Side product **11** was isolated with preparative HPLC on C18 reverse phase column by gradient A/B (60/40) → A/B 0/100)*. However, the presence of monoester **10** was detected by HPLC and NMR, yet the product **10** was not isolated in pure form.

* A: 95 parts of 0.1% aqueous solution of trifluoroacetic acid and 5 parts of acetonitrile;

B: acetonitrile.

### (3 S)-3,28-di((dimethoxyphosphoryl)methyloxy)-lup-20(29)ene 9

^1^H NMR (500 MHz, CDCl_3_) δ 4.67 (s, 1H, H_a_-C(29)), 4.57 (s, 1H, H_b_-C(29)), 3.99 (dd, ^2^*J* = 13.6 Hz, ^3^*J* = 8.7 Hz, 1H, H_a_-C(28’)), 3.87–3.76 (m, 14 H, H_2_-C(3’), (H_3_-CO)_4_), 3.70 (d, ^2^*J* = 8.7 Hz, 1H, H_a_-C(28)), 3.68 (dd, ^2^*J* = 13.6 Hz, ^3^*J* = 9.7 Hz, 1H, H_b_-C(28’)), 3.24 (d, ^2^*J* = 8.7 Hz, 1H, H_b_-C(28)), 2.83 (dd, ^3^*J* = 11.8, 4.3 Hz, 1H, H-C(3)), 2.38 (td, ^3^*J* = 10.8, 5.5 Hz, 1H, H-C(19)), 2.01–1.86 (m, 3H, H_a_-C(16), H_a_-C(21), H_a_-C(22)), 1.78–1.68 (m, 3H, H_a_-C(1), H_a_-C(15), H_a_-C(2)), 1.67 (s, 3H, H_3_-C(30)), 1.65–1.57 (m, 2H, H_a_-C(12), H-C(13)), 1.56–1.43 (m, 3H, H_a_-C(6), H_b_-C(2), H-C(18)), 1.43–1.33 (m, 5 H, H_a_-C(11), H_b_-C(6), H_b_-C(16), H_2_-C(7)), 1.28–1.13 (m, 3H, H_b_-C(11), H_b_-C(22), H-C(9)), 1.08–0.99 (m, 6 H, H_3_-C(26), H_b_-C(12), H_b_-C(15), H_b_-C(21)), 0.98 (s, 3H, H_3_-C(23)), 0.95 (s, 3H, H_3_-C(27)), 0.85–0.78 (m, 4 H, H_3_-C(25)), 0.76 (s, 3H, H_3_-C(26)), 0.67 (d, ^3^*J* = 9.5 Hz, 1H, H-C(5)). ^13^C NMR (126 MHz, CDCl_3_) δ 150.61 (C20), 109.82 (C29), 90.08 (d, ^3^*J* = 12.2 Hz, (C3)), 72.41 (d, ^3^*J* = 9.5 Hz (C28)), 65.40 (d, ^1^*J* = 164.5 Hz, (C3’)), 63.18 (d, ^1^*J* = 166.5 Hz, (C28’)), 55.81 (C5), 53.29 (d, ^2^*J* = 6.6 Hz, (MeO)) 53.14 (d, ^2^*J* = 6.8 Hz, (MeO)), 53.12 (d, ^2^*J* = 6.5 Hz, (MeO)), 53.09 (d, ^2^*J* = 6.9 Hz, (MeO)), 50.46 (C9), 48.98 (C18), 48.03 (C19), 47.56 (C17), 42.81 (C14), 41.06 (C8), 39.10 (C4), 38.52 (C1), 37.66 (C13), 37.26 (C10), 34.65 (C21), 34.31 (C7), 29.96 (C22), 29.86 (C16), 28.15 (C23), 27.20 (C15), 25.30 (C12), 22.35 (C2), 20.97 (C11), 19.25 (C30), 18.27 (C6), 16.25 (C24), 16.20 (C25), 16.10 (C26), 14.89 (C27). ^31^P NMR (121 MHz, CDCl_3_) δ 23.96, 23.73. HRMS: [C_36_H_64_O_8_P_2_ + H^+^] 687.4149; found 687.4141 (1.1 ppm). $\:[{\alpha]}_{D}^{20}=\:+0.21$; $\:[{\alpha]}_{546}^{20}=\:+0.26$; [$\:[{\alpha]}_{436}^{20}=\:+0.47$; $\:[{\alpha]}_{405}^{20}=\:+0.57$; $\:[{\alpha]}_{365}^{20}=\:+0.74$ (c = 1.00, MeOH).

### (((3 S)-3-((dimethoxyphosphoryl)methyloxy)-28-lup-20(29)enyloxy)(methoxy)phosphoryl)methyl trifluoromethanesulfonate 11

^1^H NMR (500 MHz, CDCl_3_) δ 4.68 (s, 1H, H_a_-C(29)), 4.59 (s, 1H, H_b_-C(29)), 4.27 (dd, ^2^*J* = 9.3 Hz, ^3^*J* = 5.2 Hz, 1H, H_a_-C(28)), 4.00 (dd, ^2^*J* = 13.7 Hz, ^3^*J* = 8.8 Hz, 1H, H-C(3)), 3.88–3.78 (m, 9H, (H_3_-COP)_2_, H_2_-C(28’), H_b_-C(28)), 3.70 (dd, ^2^*J* = 13.7 Hz, ^3^*J* = 9.7 Hz, 1H, H-C(3’)), 3.47 (m, 3H, OMe), 2.83 (dd, ^3^*J* = 11.8, 4.3 Hz, 1H, H-C(3)), 2.38 (ddd, ^3^*J* = 10.6, 10.1, 5.6 Hz, 1H, H-C(19)), 2.01–1.86 (m, 3H, H_a_-C(16), H_a_-C(21), H_a_-C(22)), 1.80–1.69 (m, 3H, H_a_-C(2), H_a_-C(15), H_a_-C(1)), 1.66 (s, 3H, H_3_-C(30)), 1.66–1.57 (m, 3H, H_a_-C(12), H-C(13), H-C(18)), 1.57–1.32 (m, 7 H, H_2_-C(6), H_a_-C(11), H_b_-C(2), H_b_-C(22), H-C(7)), 1.32–1.13 (m, 3H, H_b_-C(11), H_b_-C(16)), 1.13–1.03 (m, 3H, H_b_-C(12), H_b_-C(15), H_b_-C(22)), 1.02 (s, 3H, H_3_-C(26)), 0.98 (s, 3H, H_3_-C(23)), 0.96 (s, 3H, H_3_-C(27)), 0.82 (s, 3H, H_3_-C(25)), 0.82–0.75 (m, 1H, H_b_-C(1)), 0.75 (s, 3H, H_3_-C(24)), 0.67 (d, ^3^*J* = 9.6 Hz, 1H, H-C(5)). ^13^C NMR (126 MHz, CDCl_3_) δ 150.13 (C20), 110.09 (C29), 90.11 (d, ^3^*J* = 12.4 Hz, (H-C(3)), 66.33 (d, ^1^*J* = 166.44, H-C(3’)), 65.34 (d,^2^*J* = 7.6 Hz, C(28)), 63.10 (d, ^1^*J* = 167.3 Hz, H-C(28’)), 61.56 (d, ^2^*J* = 13.1 Hz, MeO-P(H_2_C(28))), 55.81 (C5), 53.40 (d, ^2^*J* = 6.5 Hz, (MeO)), 53.37 (d, ^2^*J* = 6.6 Hz, (MeO)), 50.44 (C9), 48.76 (C18), 47.82 (C19), 47.37 (d, ^3^*J* = 6.6 Hz, (C17)), 42.84 (C14), 41.04 (C8), 39.10 (C4), 38.52 (C1), 37.75 (C13), 37.26 (C10), 34.27 (C21), 34.20 (C7), 29.59 (C22), 29.30 (C16), 28.13 (C23), 26.95 (C15), 25.30 (C12), 22.33 (C2), 20.94 (C11), 19.24 (C30), 18.25 (C6), 16.23 (C24), 16.19 (C25), 16.08 (C26), 14.87 (C27). ^31^P NMR (121 MHz, CDCl_3_) δ 24.02, 22.69. R*f* = 0.43 (100% EtOAc).

### Sodium (lup-20(29)-en-(3 S)-3,28-diylbis(oxymethylene))bis(phosphonate) 12, demethylation process 9 → 12

To a solution of compound **9** (275 mg, 0.4 mmol, 1 eq.) in anhydrous DCM (5 mL) TMSI (342 µL 2.4 mmol, 6 eq.) is added dropwise at -40 °C and the resulting reaction mixture is stirred at -40 °C for 5 h. Then MeOH (2 mL) is added dropwise at -40 °C. The obtained reaction mixture is stirred for additional 30 min and solution of NaHCO_3_ (202 mg, 2.4 mmol, 6 eq.) in H_2_O (6 mL) is added dropwise at -40 °C, and the resulting mixture is warmed up to room temperature. The resulting reaction mixture is warmed up to room temperature and the organic solvents are evaporated *in vacuo*. The obtained aqueous suspension is centrifuged and the supernatant is removed and discarded. The precipitate is re-suspended in deionized water (1 mL) and the centrifugation – supernatant removal procedure is repeated additional two times (in total: washing with water 3 × 1mL). The obtained precipitate is then dried in vacuo to yield product **12** as a yellowish amorphous solid (78%, 225 mg).

^1^H NMR (500 MHz, MeOD) δ 4.69 (s, 1H, H_a_-C(29)), 4.57 (s, 1H, H_b_-C(29)), 3.85 (dd, ^2^*J* = 13.3, 9.1 Hz, 1H, H_a_-C(28’), 3.74–3.68 (m, 3H, H_a_-C(28), H_2_-C(3’)), 3.55 (dd, ^2^*J* = 13.3, 10.2 Hz, 1H, H_b_-C(28’)), 3.32(d, ^2^*J* = 11.4 Hz, 1H, H_b_-C(28)), 2.89 (dd, ^3^*J* = 11.7, 4.3 Hz, 1H, H-C(3)), 2.46 (td, ^3^*J* = 11.0, 5.9 Hz, 1H, H-C(19)), 2.08–1.98 (m, 3H, H, H_a_-C(16), H_a_-C(21), H_a_-C(22)), 1.90–1.70 (m, 5 H, H_a_-C(15), H_a_-C(1), H-C(13), H_a_-C(12), H_a_-C(2)), 1.69 (s, 3H, H_3_-C(30)), 1.62–1.39 (m, 7 H, H_2_-C(6), H_a_-C(11), H_b_-C(2), H-C(18), H_2_-C(7)), 1.39–1.10 (m, 4 H, H_b_-C(11), H_b_-C(22), H_b_-C(16), H-C(9)), 1.09 (s, 3H, H_3_-C(26)), 1.09–1.03 (m, 1H, H_b_-C(12)), 1.03 (s, 3H, H_3_-C(23)), 1.02–0.95 (m, 5 H, H_3_-C(27), H_b_-C(21), H_b_-C(15)), 0.95–0.89 (m, 1H, H_b_-C(1)), 0.88 (s, 3H, H_3_-C(25)), 0.80 (s, 3H, H_3_-C(24)), 0.74 (d, ^3^*J* = 9.6 Hz, 1H, H-C(5)). ^13^C NMR (126 MHz, MeOD) δ 150.51 (C20), 108.83 (C10), 89.45 (d,^3^*J* = 12.1 Hz, C(3)), 71.56 (d, ^3^*J* = 10.6 Hz, (C28)), 66.93 (d, ^1^*J* = 162.8 Hz, (C3’)), 64.67 (d, ^1^*J* = 164.8 Hz, C(28’)), 55.77 (C5), 50.41 (C9), 48.79 (C18), 48.01 (C19), 42.40 (C14), 40.77 (C8), 38.69 (C1), 38.33 (C4), 37.51 (C13), 36.90 (C10), 34.30 (C21), 34.05 (C7), 29.58 (C22), 29.49 (C16), 27.19 (C23), 26.97 (C15), 25.20 (C12), 21.92 (C2), 20.60 (C11), 17.98 (C30), 17.89 (C6), 15.34 (C25), 15.32 (C24), 15.22 (C26), 13.85 (C27). ^31^P NMR (121 MHz, MeOD) δ 19.72, 19.07. $\:[{\alpha]}_{D}^{20}=\:+0.11$; $\:[{\alpha]}_{546}^{20}=\:+0.17$; [$\:[{\alpha]}_{436}^{20}=\:+0.33$; $\:[{\alpha]}_{405}^{20}=\:+0.37$; $\:[{\alpha]}_{365}^{20}=\:+0.46$ (c = 1.00, MeOH).

### Cytotoxicity evaluation

Cytotoxicity of betulinic acid, ursolic acid and oleanolic acid derivatives was evaluated using human-derived osteosarcoma cell line MG63 (ATCC, CRL-1427) and mouse-derived preosteoblast cell line MC3T3-E1 (ATCC CRL-2593). Before conducting the experiments, both cell lines were continuously cultured according to the ATCC product sheet instructions. Briefly, both cell lines were expanded in α-MEM medium supplemented with 10% FBS and 1% pen-strep and maintained at 37 °C in a humidified atmosphere with 5% CO_2_.

Each cell line was plated at a concentration of 1 × 10^4^ cells/well in 96-well plate. The plates were incubated for 24 h to allow cell attachment and growth. Bioactive substances were solubilized in methanol prior further dilution in cell culture media. Following the cell incubation period, the culture media was removed and the dilutions of active substance (10, 25 and 50 µM) in cell culture media were added. The culture medium only and its dilutions with methanol solution respective to the bioactive substance concentrations were used as the controls. The final concentration of methanol did not exceed 1%. Cells were treated with bioactive substance solutions for 24 h. After 24 h incubation, relative cell metabolic activity was assessed using CellTiter-Blue^®^ (CTB) analysis (Promega, JAV). Absorbance was measured at 590 nm using microplate reader (Infinite^®^ 200 PRO, Tecan, USA). The relative cell metabolic activity was calculated for each bioactive substance concentration as well as for the controls. All results were presented as the mean ± standard deviation of at least 5 replicates. Statistically significant differences between sample groups are assessed using a one-way ANOVA test and then corrected using the Šídák multiple comparison test. Statistically significant differences were considered to be those with a P-values less than 0.05 (*p* < 0.05). Numerical values of the cell relative metabolic activity are summarized in Table 4 and the corresponding graphical representation can be found in supporting information (Figures S1-S4).

## Electronic supplementary material

Below is the link to the electronic supplementary material.

Supplementary Material 1

## Data Availability

All data generated or analysed during this study are included in this published article (and its Supplementary Information files).
