# Supplementary material for: Synthesis and preliminary cytotoxicity evaluation of water soluble pentacyclic triterpenoid phosphonates
Source: Sci Rep. 2024 Nov 14;14:28031. doi: 10.1038/s41598-024-76816-w (PMC11564732; doi:10.1038/s41598-024-76816-w)
Supplement: Supplementary file 1 — Supplementary Material 1 [file 41598_2024_76816_MOESM1_ESM.pdf]

Supplementary Information

Synthesis and preliminary cytotoxicity evaluation of water soluble pentacyclic triterpenoid phosphonates

Jevgeņija Lugiņina[1], Vladislavs Kroškins[1], Rihards Lācis[1], Elza Fedorovska[1], Öznur Demir[2,3], Arita Dubnika[2,3], Dagnija Loca[2,3] and Māris Turks[1,*]

Affiliation [1] Institute of Chemistry and Chemical Technology, Faculty of Natural Sciences and Technology, Riga Technical University, 3 P.Valdena Street, Riga, LV-1048, Latvia; [2] Institute of Biomaterials and Bioengineering, Faculty of Natural Sciences and Technology, Riga Technical University, 3 Pulka Street, Riga, LV-1048, Latvia; [3] Baltic Biomaterials Centre of Excellence, Headquarters at Riga Technical University, Riga, Latvia; Corresponding author e-mail: maris.turks@rtu.lv

General information

Solvents for the reactions were dried over standard drying agents and freshly distilled prior to use. All purchased chemicals (Fluka, Aldrich) were used as received. All reactions were followed by TLC on E. Merck Kieselgel 60 F_{254} and visualized by using UV lamp. Column chromatography was performed on silica gel (60 Å, 40-63 μm, ROCC). Flash column chromatography was performed on a Büchi Sepacore system (Büchi-Labortechnik GmbH, Essen, Germany) with a Büchi Control Unit C-620, an UV detector Büchi UV photometer C-635, Büchi fraction collector C-660 and two Pump Modules C-605. ^{1}H and ^{13}C NMR spectra were recorded on a Bruker 300 and 500 MHz, in $CDCl_3$ or [D_4]MeOD at 25 °C. Chemical shifts (δ) values are reported in ppm. The residual solvent peaks are used as internal reference ($CDCl_3$) 7.26 ppm, [D_4]MeOD 3.31 ppm for ^{1}H NMR, $CDCl_3$ 77.16 ppm, [D_4]MeOD 49.00 ppm for ^{13}C NMR), s (singlet), d (doublet), t (triplet), q (quartet), m (multiplet); *J* in hertz. ^{1}H and ^{13}C NMR peaks were assigned by analysis of multidimensional NMR (COSY, HSQC, HMBC). For ^{31}P NMR calibration, Ph_3P was used as external reference (-6.00 ppm in $MeOD_{d4}$) in a coaxially inserted tube. High-resolution massspectra (ESI) were performed on Agilent 1290 Infinity series UPLC connected to Agilent 6230 TOF mass spectrometer (calibration at m/z 121.050873 and m/z 922.009798). Optical

rotation was measured at 20 °C on *Anton Paar MCP 500* polarimeter (1-cm cell) using multi-wavelenght analysis (589 nm, 546 nm, 436 nm, 405 nm, 365 nm).

General procedure I for the synthesis of 3-oxo-triterpenic acid esters, process 2a-c → 3a-c

To solution of 3-oxo-triterpenic acid **2a-c** (500 mg, 1.099 mmol, 1 eq.) in anhydrous THF (5 mL) *t*BuOK (185mg, 1.649 mmol, 1 eq.) is added portion wise at -5 °C. The resulting reaction mixture is stirred in ambient temperature for 30 min, then warmed up to room temperature and stirred for additional 60 min. The obtained mixture is re-cooled to -5 °C and a solution of previously prepared (dimethoxyphosphoryl)methyl trifluoromethanesulfonate (629 mg, 2.198 mmol, 2 eq.) in anhydrous THF (5 mL) is added dropwise. Then the resulting reaction mixture is warmed up to room temperature and stirred for 12 h. The reaction mixture is evaporated to dryness, redissolved in EtOAc (70 mL) and washed with brine (3 × 10 mL). Separated organic layer is dried over anhydrous Na_2SO_4. After filtration, the filtrate is concentrated *in vacuo* and purified by silica column chromatography (Hexanes-EtOAc 9:1 → 1:1) to yield carboxylic ester as a white amorphous solid: **3a** (78%, 495 mg); **3b** (61%, 387 mg), **3c** (71%, 450 mg).

3-oxo-(17S)-17-(((dimethoxyphosphoryl)methoxy)carbonyl)-28-norlup-20(29)-ene 3a

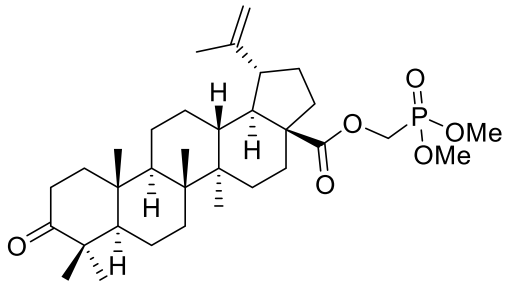

^{1}H NMR (500 MHz, $CDCl_3$) δ 4.73 (s, 1H, H_a-C(29)), 4.60 (s, 1H, H_b-C(29)), 4.48 (dd, ^2J = 14.6, 8.3 Hz, 1H, H_a-C(28’)), 4.39 (dd, ^2J = 14.6, 8.2 Hz, 1H, H_b-C(28’)), 3.81 (d, ^3J = 10.9, 6H, $(OMe)_2$), 2.98 (td, ^3J = 11.1, 4.8 Hz, 1H, H-C(19)), 2.48 (ddd, ^2J = 15.5 Hz, ^3J = 9.8, 7.5 Hz, 1H, H_a-C(2)), 2.39 (ddd, ^2J = 15.5 Hz, ^3J = 7.6, 4.4 Hz, 1H, H_b-C(2)), 2.31 – 2.19 (m, 2H, H_a-C(16), H-C(13)), 1.96 – 1.82 (m, 4H, H_a-C(15), H_a-C(21), H_a-C(22), H_a-C(1)), 1.76 – 1.69 (m, 1H, H_a-C(12)), 1.68 (s, 3H, H_3-C(30)), 1.62 (t, ^3J = 11.4 Hz, 1H, H-C(18)), 1.54 – 1.34 (m, 9H, H_2-C(6), H_a-C(11), H_2-C(7), H_b-C(21), H_b-C(1), H-C(9), H_b-C(16)), 1.34 – 1.14 (m, 4H, H_b-C(11), H_b-C(15), H_b-C(22), H-C(5)), 1.06 (s, 3H, H_3-C(23)), 1.06 – 1.01 (m, 1H, H_b-C(12)), 1.01 (s, 3H, H_3-

C(24)), 0.97 (s, 3H, H_3-C(27)), 0.96 (s, 3H, H_3-C(26)), 0.92 (s, 3H, H_3-C(25)). ^{13}C NMR (126 MHz, $CDCl_3$) δ 218.25 (C3), 174.91 (d, ^3J = 6.4 Hz, (C28)) , 150.34 (C20), 109.96 (C29), 56.90 (C17), 55.18 (d, ^1J = 167.4 Hz), 55.10 (C5), 53.20 (d, ^2J = 6.2 Hz, MeO), 53.18 (d, ^2J = 6.2 Hz, MeO), 50.04 (C9), 49.49 (C18), 47.46 (C4), 46.90 (C19), 42.61 (C14), 40.77 (C10), 39.77 (C1), 38.44 (C13), 37.04 (C21), 37.00 (C10), 34.26 (C2), 33.75 (C7), 32.03 (C16), 30.53 (C22), 29.65 (C15), 26.74 (C23), 25.65 (C12), 21.54 (C11), 21.15 (C24), 19.76 (C30), 19.47 (C6), 16.10 (C25), 15.85 (C26), 14.74 (C27). ^{31}P NMR (121 MHz, $CDCl_3$) δ 21.77. HRMS: [$C_{33}H_{53}O_6P+H^+$] 577.3653; found 577.3626 (4.7 ppm). $[\alpha]_D^{20} = +0.19$; $[\alpha]_{546}^{20} = +0.23$; $[\alpha]_{436}^{20} = +0.50$; $[\alpha]_{405}^{20} = +0.66$; $[\alpha]_{365}^{20} = +1.02$ (c = 1.00, MeOH).

3-oxo-(17S)-17-(((dimethoxyphosphoryl)methoxy)carbonyl)-28-norolean-12(13)-ene 3b

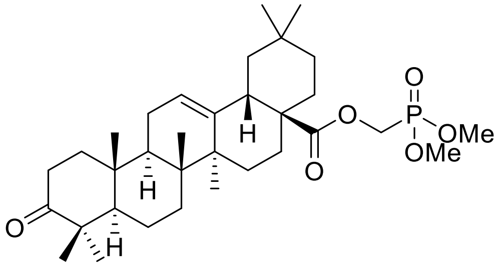

^{1}H NMR (500 MHz, $CDCl_3$): δ 5.32 (t, ^3J = 3.6 Hz, 1H, H-C(12)), 4.42 (dd, ^2J = 14.0, 8.6 Hz, 1H, H_a-C(28`)), 4.32 (dd, $^2J$ = 14.0, 8.6 Hz, 1H, $H_b$-C(28`)), 3.81 (d, ^2J = 10.8 Hz, 6H, $(MeO)_2$), 2.88 (dd, ^3J = 14.0, 4.3 Hz, 1H, H-C(18)), 2.55 (ddd, ^2J = 15.9 Hz, ^3J = 11.2, 7.2 Hz, 1H, H_a-C(2)), 2.36 (ddd, ^2J = 15.9 Hz, ^3J = 6.8, 3.6 Hz, 1H, H_b-C(2)), 2.01 (dt, ^2J = 14.0 Hz, ^3J = 4.3 Hz, 1H, H_a-C(16)), 2.00 - 1.90 (m, 2H, H_2-C(11)), 1.88 (ddd, ^2J = 13.2 Hz, ^3J = 7, 3.6 Hz, 1H, H_a-C(1)), 1.72 (dt, 1H, ^2J = 13.6 Hz, ^3J = 4.1 Hz, H_a-C(22)), 1.69 – 1.53 (m, 5H, H_b-C(16), H_a-C(19), H-C(9), H_a-C(7), H_a-C(15)), 1.52 – 1.47 (m, 3H, H_2-C(6), H_b-C(7)), 1.40 (dt, ^2J = 12.3 Hz, ^3J = 6.0 Hz, 1H, H_b-C(1)), 1.35 – 1.28 (m, 4H, H_b-C(22), H_a-C(21), H-C(5), H_b-C(6)), 1.26 – 1.08 (m, 3H, H_b-C(21), H_b-C(19), H_b-C(15)), 1.14 (s, 3H, H_3-C(27)), 1.08 (s, 3H, H_3-C(23)), 1.04 (s, 6H, H_3-C(24), H_3-C(25)), 0.92 (s, 3H, H_3-C(29)), 0.90 (s, 3H, H_3-C(30)), 0.78 (s, 3H, H_3-C(26)). ^{13}C NMR (126 MHz, $CDCl_3$): δ 217.90 (C(3)), 176.77 (d, ^3J= 8.0 Hz, (C28)), 143.55 (C13), 122.65 (C12), 55.82 (d, ^1J = 168.7 Hz, (C28`)), 55.47 (C5), 53.24 (d, ^2J= 6.4 Hz, $(MeO)_2$), 47.60 (C4), 47.25 (C17), 46.98 (C9), 45.90 (C19), 41.96 (C14), 41.54 (C18), 39.40 (C8), 39.29 (C1), 36.89 (C10), 34.31

(C2), 33.91 (C21), 33.16 (C30), 32.37 (C7), 32.32 (C22), 30.81 (C20), 27.75 (C15), 26.56 (C23), 25.87 (C27), 23.66 (C29), 23.65 (C11), 23.19 (C16), 21.63 (C24), 19.72 (C6), 16.95 (C26), 15.18 (C25). ^{31}P NMR (121 MHz, $CDCl_3$) δ 21.74. HRMS: [$C_{33}H_{53}O_6P+H^+$] 577.3653; found 577.3626 (4.7 ppm). $[\alpha]_D^{20} = +0.66$; $[\alpha]_{546}^{20} = +0.79$; $[\alpha]_{436}^{20} = +1.38$; $[\alpha]_{405}^{20} = +1.70$; $[\alpha]_{365}^{20} = +2.28$ (c = 1.00, MeOH).

3-oxo-(17S)-17-(((dimethoxyphosphoryl)methoxy)carbonyl)-28-norurs-12(13)-ene 3c

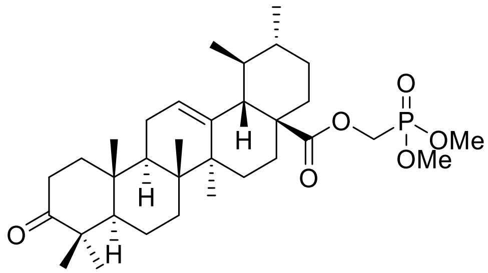

^{1}H NMR (500 MHz, $CDCl_3$) δ 5.29 (d, ^3J = 3.8 Hz, 1H, H-C(12)), 4.33 (d, ^2J = 8.5 Hz, 2H, H_2-C(28')), 3.80 (d, ^3J = 10.8 Hz, 6H, $(OMe)_2$), 2.55 (ddd, ^2J = 16.0 Hz, ^3J = 10.8, 7.5 Hz, 1H, H_a-C(2)), 2.37 (ddd, ^2J = 16.0 Hz, ^3J = 6.9, 3.6 Hz, 1H, H_b-C(2)), 2.25 (d, ^3J = 11.3 Hz, 1H, H-C(18)), 2.04 (td, ^2J = 14.4, ^3J = 6.5 Hz, 1H, H_a-C(16)), 1.99 – 1.92 (m, 2H, H_2-C(11)), 1.90 (ddd, ^2J = 12.0 Hz, ^3J = 7.4, 3.6 Hz, 1H, H_a-C(1)), 1.80 – 1.68 (m, 3H, H_b-C(16), H_a-C(15), H_a-C(21)), 1.65 – 1.55 (m, 2H, H_b-C(21), H-C(9)), 1.55 – 1.39 (m, 5H, H_2-C(6), H_a-C(15), H_a-C(7), H_b-C(1)), 1.39 – 1.23 (m, 4H, H_b-C(22), H_b-C(7), H-C(19, H-C(5)), 1.17 – 1.11 (m, 1H, H_b-C(15)), 1.09 (s, 3H, H_3-C(27)), 1.08 (s, 3H, H_3-C(28)), 1.04 (s, 6H, H_3-C(24), H_3-C(25)), 1.04 – 0.98 (m, 1H, H-C(20)), 0.94 (d, ^3J = 6.3 Hz, 3H, H_3-C(30)), 0.86 (d, ^3J = 6.4 Hz, 3H, H_3-C(29)), 0.79 (s, 3H, H_3-C(26)). ^{13}C NMR (126 MHz, $CDCl_3$) δ 217.95 (C3), 176.62 (d, ^3J = 8.2 Hz, (C28)), 138.02 (C13), 125.83 (C12), 55.81 (d, ^1J = 168.9 Hz, (C28')), 55.13 (C5), 53,24 (d, ^2J = 6.1 Hz, (OMe)), 53.21 (d, ^2J = 6.1 Hz, (OMe)), 53.07 (C18), 48.64 (C17), 47.55 (C4), 46.86 (C9), 42.28 (C14), 39.60 (C8), 39.44 (C1), 39.17 (C19), 38.91 (C20), 36.79 (C10), 36.58 (C21), 34.31 (C2), 32.65 (C7), 30.68 (C22), 28.08 (C15), 26.67 (C23), 24.32 (C16), 23.59 (C27), 23.58 (C11), 21.63 (C24), 21.24 (C30), 19.71 (C6), 17.12 (C29), 17.05 (C26), 15.37 (C25). ^{31}P NMR (121 MHz, $CDCl_3$) δ 21.85. HRMS: [$C_{33}H_{53}O_6P+H^+$] 577.3653; found 577.3623 (5.2 ppm). $[\alpha]_D^{20} = +0.50$; $[\alpha]_{546}^{20} = +0.58$; $[\alpha]_{436}^{20} = +0.97$; $[\alpha]_{405}^{20} = +1.23$; $[\alpha]_{365}^{20} = +1.62$ (c = 1.00, MeOH).

General procedure II for synthesis of 3-hydroxy-triterpenic acid esters, process 3a-c → 4a-c

To solution of 3-oxo-triterpenoic acid ester **3a-c** (200 mg, 0.347, 1 eq.) in MeOH (4 mL) $NaBH_4$ (53 mg, 1.388 mmol, 4 eq.) is added portion wise at 0 °C . The resulting reaction mixture is stirred at ambient temperature for 5 h. Then the reaction mixture is quenched by NH_4Cl saturated aqueous solution (2 mL), evaporated to dryness, redissolved in EtOAc (25 mL) and washed with brine (3 × 10 mL). The combined organic layer is dried over anhydrous Na_2SO_4. After filtration, the filtrate is concentrated *in vacuo* and purified by silica column chromatography (Hexanes-EtOAc 9:1 → 1:1) to yield product as a white amorphous solid: **4a** (99%, 198 mg); **4b** (96%, 193 mg); **4c** (92%, 185 mg).

(17S)-17-(((dimethoxyphosphoryl)methoxy)carbonyl)-3β-hydroxy-28-norlup-20(29)-ene 4a

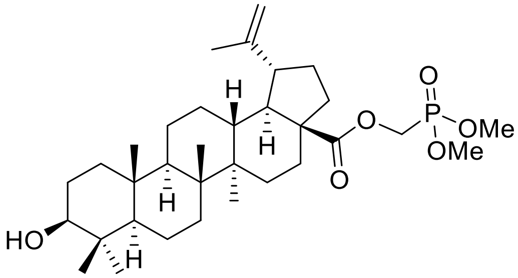

^{1}H NMR (500 MHz, $CDCl_3$) δ 6.02 (bs, 1H, OH), 4.73 (s, 1H, H_a-C(29)), 4.61 (s, 1H, H_b-C(29)), 4.48 (dd, ^2J = 14.7, 8.2 Hz, 1H, H_a-C(28’), 4.39 (dd, ^2J = 14.7, 8.2 Hz, 1H, H_b-C(28’), 3.81 (d, ^3J = 10.8 Hz, 6H, $(OMe)_2$), 3.18 (dd, ^3J = 11.4, 4.7 Hz, 1H, H-C(3)), 2.98 (td, ^3J = 11.2, 4.7 Hz, 1H, H-C(19)), 2.30 – 2.34 (m, 1H, H_a-C(16)), 2.24 – 2.15 (m, 1H, H-C(13)), 1.98 – 1.83 (m, 2H, H_a-C(21), H_a-C(22)), 1.74 – 1.31 (m, 17H, H_a-C(15), H_3-C(C30), H_a-C(12), H-C(18), H_2-C(6), H_a-C(11), H_2-C(7), H_b-C(21), H_a-C(1), H-C(9), H_b-C(16), H_2-C(2)), 1.31 – 1.13 (m, 3H, H_b-C(11), H_b-C(15), H_b-C(22)), 1.08 – 0.99 (m, 1H, H_b-C(12)), 0.96 (s, 6H, H_3-C(27), H_3-C(26)), 0.92 (s, 3H, H_3-C(23)), 0.90 – 0.85 (m, 1H, H_b-C(1)), 0.82 (s, 3H, H_3-C(25)), 0.75 (s, 3H, H_3-C(24)), 0.68 (d, ^3J = 9,4 Hz, 1H, H-C(5)). ^{13}C NMR (126 MHz, $CDCl_3$) δ 174.97 (d, ^3J = 6.4 Hz, (C28)), 150.46 (C20), 109.91 (C29), 79.12 (C3), 56.96 (C17), 55.0 (C5), 55.17 (d, ^1J = 167.4 Hz, C(28’)), 53.20 (d, ^2J = 6.1 Hz, (MeO)), 53.17 (d, ^2J = 6.1 Hz, (MeO)), 50.70 (C9), 49.58 (C18), 46.96 (C19), 42.58 (C14), 40.85 (C8), 39.01 (C4), 38.88 (C1), 38.39 (C13), 37.35 (10), 37.05 (C21), 34.48 (C7),

32.12 (C16), 30.57 (C22), 29.70 (C15), 28.13 (C23), 27.55 (C2), 25.67 (C12), 21.03 (C11), 19.49 (C30), 18.44 (C6), 16.30 (C25), 16.07 (C26), 15.50 (C24), 14.85 (C27). ^{31}P NMR (121 MHz, $CDCl_3$) δ 21.88. HRMS: [$C_{33}H_{55}O_6P+H^+$] 579.3809; found 579.3782 (4.7 ppm). $[\alpha]_D^{20} = -0.01$; $[\alpha]_{546}^{20} = -0.01$; $[\alpha]_{436}^{20} = +0.01$; $[\alpha]_{405}^{20} = +0.02$; $[\alpha]_{365}^{20} = +0.02$ (c = 1.00, MeOH).

(17S)-17-(((dimethoxyphosphoryl)methoxy)carbonyl)-3β-hydroxy -28-norolean-12(13)-ene 4b

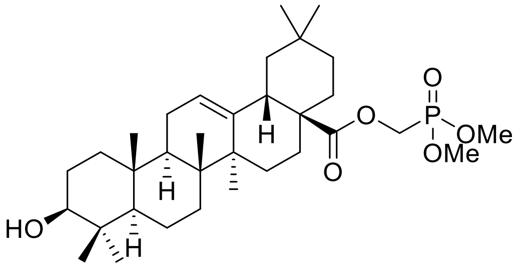

^{1}H NMR (500 MHz, $CDCl_3$) δ 5.30 (t, ^3J = 3.6 Hz, 1H, H-C(13)), 4.41 (dd, ^2J = 14.6, 8.7 Hz, 1H, H_a-C(28')), 4.31 (dd, ^2J = 14.6, 8.3 Hz, 1H, H_b-C(28')), 3.80 (d, ^3J = 10.9 Hz, 6H, $(OMe)_2$), 3.25 – 3.17 (m, 1H, H-C(3)), 2.90 – 2.81 (m, 1H, H-C(18)), 2.07 – 1.95 (m, 1H, H_a-C(16)), 1.91 – 1.85 (m, 2H, H_2-C(11)), 1.77 – 1.16 (m, 16H, H_2-C(6), H_b-C(16), H_2-C(2), H_a-C(15), H_2-C(22), H_2-C(7), H_2-C(21), H-C(9), H_a-C(1), H_2-C(19)), 1.13 (s, 3H, H_3-C(27)), 1.09 (d, ^2J = 14.0 Hz, 1H, H_b-C(15)), 0.98 (s, 3H, H_3-C(23)), 0.98 – 0.92 (m, 1H, H_b-C(1)), 0.92 (s, 3H, H_3-C(29)), 0.90 (s, 6H, H_3-C(30), H_3-C(25)), 0.78 (s, 3H, H_3-C(24)), 0.77 – 0.72 (m, 1H, H-C(5)), 0.72 (s, 3H, H_3-C(26)). ^{13}C NMR (126 MHz, $CDCl_3$) δ 176.81 (d, ^3J = 7.8 Hz, (C28)), 143.49 (C12), 122.89 (C12), 79.16 (C3), 55.92 (d, ^1J = 169.5 Hz, (C28')), 55.13 (C5)), 53.23 (d, ^2J = 6.3 Hz, (OMe)), 53.23 (d, ^2J = 6.3 Hz, (OMe)), 47.73 (C9), 47.24 (C17), 45.97 (C19), 41.85 (C14), 41.48 (C18), 39.43 (C8), 38.90 (C4), 38.60 (C1), 37.18 (C10), 33.93 (C21), 33.18 (C30), 32.86 (C7), 32.38 (C22), 30.81 (C20), 28.25 (C23), 27.77 (C15), 27.34 (C2), 25.99 (C27), 23.69 (C29), 23.57 (C11), 23.21 (C16), 18.48 (C6), 17.02 (C26), 15.72 (C24), 15.48 (C25). ^{31}P NMR (121 MHz, $CDCl_3$) δ 21.77. HRMS: [$C_{33}H_{55}O_6P+NH_4^+$] 596.4075; found 596.4042 (5.5 ppm). $[\alpha]_D^{20} = +0.38$; $[\alpha]_{546}^{20} = +0.46$; $[\alpha]_{436}^{20} = +0.79$; $[\alpha]_{405}^{20} = +0.97$; $[\alpha]_{365}^{20} = +1.27$ (c = 1.00, MeOH).

(17S)-17-(((dimethoxyphosphoryl)methoxy)carbonyl)-3β-hydroxy-28-norurs-12(13)-ene 4c

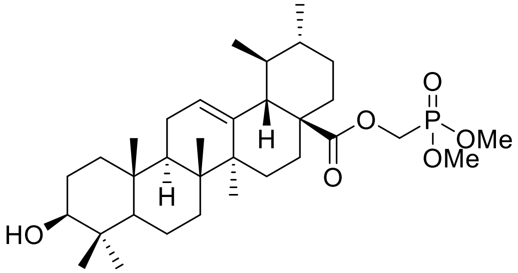

^{1}H NMR (500 MHz, $CDCl_3$) δ 5.26 (bs, 1H, H-C(13)), 4.33 (d, ^{2}J = 8.4 Hz, 2H, H_2-C(28’)), 3.80 (d, ^{3}J = 10.8 Hz, 6H, $(OMe)_2$), 3.21 (dd, ^{3}J = 11.3, 4.6 Hz, 1H, H-C(3)), 2.23 (d, ^{3}J = 11.3 Hz, 1H, H-C(18)), 2.10 – 1.99 (m, 1H, H_a-C(16)), 1.95 – 1.84 (m, 2H, H_2-C(11)), 1.79 – 1.68 (m, 3H, H_b-C(16), H_a-C(15), H_a-C(21)), 1.67 – 1.22 (m, 12H, H_b-C(21), H_a-C(1), H_2-C(2), H_2-C(6), H_2-C(7), H_2-C(22), H-C(9), H-C(19)), 1.13 – 1.08 (m, 1H, H_b-C(15)), 1.08 (s, 3H, H_3-C(27)), 1.04 – 0.99 (m, 1H, H-C(20)), 0.99 (s, 3H, H_3-C(23)), 0.99 – 0.94 (m, 1H, H_b-C(1)), 0.94 (d, ^{3}J = 6,3 Hz, 3H, H_3-C(30)), 0.92 (s, 3H, H_3-C(25)), 0.86 (d, ^{3}J = 6.6 Hz, 3H, H_3-C(29)), 0.78 (s, 3H, H_3-C(24)), 0.73 (s, 3H, H_3-C(26)), 0.73 – 0.69 (m, 1H, H-C(5)). ^{13}C NMR (126 MHz, $CDCl_3$) δ 176.66 (d, ^{3}J = 8.3 Hz, (C28)), 137.93 (C12), 126.08 (C13), 79.18 (C3), 55.81 (d, ^{1}J = 168.9 Hz, (C28’)), 55.36 (C5), 53.25 (d, ^{2}J = 6.4 Hz, (MeO)), 53.25 (d, ^{2}J = 6.4 Hz, (MeO)), 53.03 (C18), 48.63 (C17), 47.67 (C9), 42.18 (C14), 39.65 (C8), 39.19 (C19), 38.95 (C20), 38.90 (C4), 38.77 (C1), 37.11 (C10), 36.64 (C21), 33.15 (C7), 30.72 (C22), 28.28 (C23), 28.12 (C25), 27.37 (C2), 24.37 (C16), 23.70 (C27), 23.45 (C11), 21.28 (C30), 18.46 (C6), 17.13 (C26), 17.09 (C29), 15.76 (C24), 15.62 (C25). ^{31}P NMR (121 MHz, $CDCl_3$) δ 21.77. HRMS: [$C_{33}H_{55}O_6P+H^+$] 579.3809; found 579.3781 (4.8 ppm). $[\alpha]_D^{20} = +0.36$; $[\alpha]_{546}^{20} = +0.43$; $[\alpha]_{436}^{20} = +0.76$; $[\alpha]_{405}^{20} = +0.92$; $[\alpha]_{365}^{20} = +1.20$ (c = 1.00, MeOH).

General procedure III for demethylation of phosphonic esters, processes 3a-b → 7a-c and 4a-c → 8a-c

To solution of 3-oxo-triterpenic acid ester **3a-c** or 3-hydroxy-triterpenoic acid ester **4a-c** (0.4 mmol, 1 eq.) in anhydrous DCM (5 mL) TMSI (171 µL 1.2 mmol, 3 eq.) is added dropwise at -40 °C and the resulting reaction mixture is stirred at -40 °C for 5 h. Then MeOH (2.5 mL) is added dropwise at -40 °C. The obtained mixture is stirred for additional 30 min at the same temperature and solution of $NaHCO_3$ (101mg, 1.2 mmol, 3 eq.) in H_2O (4 mL) is added dropwise at -40 °C. The resulting reaction mixture is warmed up to room temperature and the organic solvents are evaporated *in vacuo*. The obtained aqueous suspension is centrifuged and the supernatant is removed and discarded. The precipitate is re-suspended in deionized water (1 mL) and the centrifugation – supernatant removal procedure is repeated additional two times (in total: washing with water 3 × 1mL). The obtained precipitated is dried at ambient temperature *in vacuo*: **7a** (97%, 230 mg); **7b** (90%, 214 mg); **7c** (51%, 121 mg); **8a** (91%, 217 mg); **8b** (78%, 186 mg); **8c** (93%, 222 mg).

Sodium (3-oxo-(17R)-17-28-norlup-20(29)-en)-2-oxoethyl-phosphonate 7a

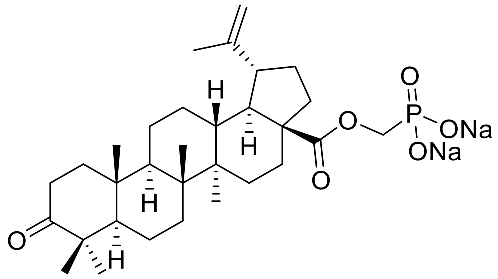

^{1}H NMR (500 MHz, MeOD$_{d4}$) δ 4.72 (s, 1H, H$_a$-C(29)), 4.58 (s, 1H, H$_b$-C(29)), 4.16 (dd, ^2J = 13.1, 8.7 Hz, 1H, H$_a$-C(28')), 3.90 (dd, ^2J = 13.1, 8.4 Hz, 1H, H$_b$-C(29)), 3.02 (td, ^3J = 11.2, 4.4 Hz, 1H, H-C(19)), 2.58 – 2.36 (m, 4H, H$_2$-C(2), H-C(13), H$_a$-C(16)), 2.24 (dd, ^3J = 11.7, 8.1 Hz, 1H, H$_a$-C(21)), 2.00 – 1.86 (m, 2H, H$_a$-C(21), H$_b$-C(22)), 1.76 (d, ^3J = 13.0 Hz, 1H, H$_a$-C(12)), 1.69 (s, 3H, H$_3$-C(30)), 1.63 (t, ^3J = 11.3 Hz, 1H, H-C(18)), 1.57 – 1.27 (m, 13H, H$_2$-C(6), H$_2$-C(11), H$_2$-C(7), H$_a$-C(15), H$_b$-C(22), H$_b$-C(16), H$_b$-C(1), H-C(9), H$_b$-C(21), H-C(5)), 1.23 – 1.15 (m, 1H, H$_b$-C(22)), 1.13 – 1.06 (m, 1H, H$_b$-C(12)), 1.06 (s, 3H, H$_3$-C(23)), 1.02 (s, 3H, H$_3$-C(24)), 1.01 (s, 3H, H$_3$-C(27)), 1.00 (s, 3H, H$_3$-C(26)), 0.95 (s, 3H, H$_3$-C(25)). ^{13}C NMR (126 MHz,

MeOD$_{d4}$) δ 221.02 (C3), 176.99 (d, ^3J = 8.4 Hz, (C28)), 151.94 (C20), 110.22 (C29), 59.92 (d, ^1J = 162.6 Hz, (C28')), 57.99 (C17), 56.11 (C5), 51.23 (C9), 50.68 (C18), 48.31 (C19), 43.61 (C14), 41.87 (C8), 40.70 (C4), 39.60 (C11), 38.06 (C13), 37.79 (C10), 35.05 (C21), 34.74 (C7), 34.72 (C16), 32.92 (C2), 31.61 (C22), 30.88 (C15), 27.17 (C23), 26.89 (C12), 22.62 (C11), 21.43 (C24), 20.76 (C6), 19.55 (C30), 16.54 (C26), 16.35 (C25), 15.03 (C27). ^{31}P NMR (121 MHz, MeOD$_{d4}$) δ 14.20. HRMS: [$C_{31}H_{49}O_6P$-H^+] 547.3194; found 547.3198 (0.7 ppm). $[\alpha]_D^{20} = +0.16$; $[\alpha]_{546}^{20} = +0.19$; $[\alpha]_{436}^{20} = +0.38$; $[\alpha]_{405}^{20} = +0.51$; $[\alpha]_{365}^{20} = +0.80$ (c = 1.00, MeOH).

Sodium (3-oxo-(17R)-17-28-norolean-12(13)-en)-2-oxoethyl-phosphonate 7b

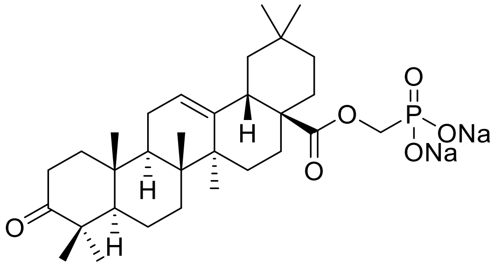

^{1}H NMR (500 MHz, MeOD$_{d4}$) δ 5.30 (d, ^3J = 3.7 Hz, 1H, H-C(13)), 4.21 – 4.09 (m, 2H, H_2-C(28')), 2.92 (dd, ^3J = 14.1, 4.5 Hz, 1H, H-C(18)), 2.57 (ddd, ^2J = 16.1 Hz, ^3J = 10.8, 7.4 Hz, 1H, H_a-C(2)), 2.37 (ddd, ^2J = 16.1 Hz, ^3J = 7.1, 3.6 Hz, 1H, H_b-C(2)), 2.09 – 1.86 (m, 4H, H_a-C(16), H_2-C(11), H_a-C(1)), 1.84 – 1.60 (m, 6H, H_b-C(16), H_a-C(15), H_2-C(22), H_a-C(19), H-C(9)), 1.59 – 1.32 (m, 7H, H_2-C(6), H_2-C(7), H_a-C(21), H_b-C(1), H-C(5)), 1.21 (d, ^2J = 13.2 Hz, 1H, H_b-C(21)), 1.18 (s, 3H, H_3-C(27)), 1.16 – 1.09 (m, 2H, H_b-C(15), H_b-C(19)), 1.08 (s, 6H, H_3-C(23), H_3-C(25)), 1.05 (s, 3H, H_3-C(24)), 0.95 (s, 3H, H_3-C(29)), 0.91 (s, 3H, H_3-C(30)), 0.83 (s, 3H, H_3-C(25)). ^{13}C NMR (126 MHz, MeOD$_{d4}$) δ 220.52 (C3), 180.23 (d, ^3J = 9.0 Hz, (C28)), 145.38 (C12), 123.35 (C13), 60.43 (d, ^1J = 162.8 Hz, (C28')), 56.60 (C5), 48.83 (C4), 48.53 (C17), 48.26 (C9), 47.32 (C19), 42.98 (C14), 42.93 (C18), 40.56 (C8), 40.27 (C1), 37.92 (C10), 35.11 (C2), 35.00 (C21), 33.64 (C30), 33.38 (C7), 33.10 (C22), 31.58 (C20), 29.14 (C15), 26.96 (C23), 26.30 (C27), 24.59 (C11), 24.15 (C29), 23.78 (C16), 21.90 (C24), 20.73 (C6), 17.54 (C26), 15.53 (C25). ^{31}P NMR (121 MHz, MeOD$_{d4}$) δ 11.54. HRMS: [$C_{31}H_{49}O_6P$-H^+] 547.3194; found 547.3194 (0 ppm). $[\alpha]_D^{20} = +0.47$; $[\alpha]_{546}^{20} = +0.56$; $[\alpha]_{436}^{20} = +0.98$; $[\alpha]_{405}^{20} = +1.22$; $[\alpha]_{365}^{20} = +1.65$ (c = 1.00, MeOH).

Sodium (3-oxo-(17R)-17-28-norurs-12(13)-en)-2-oxoethyl-phosphonate 7c

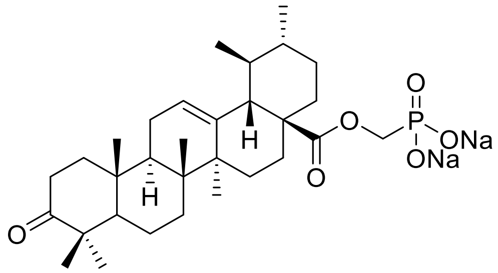

^{1}H NMR (500 MHz, MeOD$_{d4}$) δ 5.30 (d, ^3J = 3.7 Hz, 1H, H-C(12)), 4.08 (d, ^2J = 13.2 Hz, 1H, H$_a$-C(28')), 4.00 (d, ^2J = 13.2 Hz, 1H, H$_b$-C(28')), 2.57 (ddd, ^2J = 16.0 Hz, ^3J = 10.7, 7.4 Hz, 1H, H$_a$-C(2)), 2.39 (ddd, ^2J = 16.0 Hz, ^3J = 7.1, 3.7 Hz, 1H, H$_b$-C(2)), 2.32 (d, ^3J = 11,3 Hz, 1H, H-C(18)), 2.10 – 1.90 (m, 5H, H$_2$-C(11), H$_a$-C(16), H$_a$-C(15), H$_a$-C(1)), 1.90 – 1.81 (m, 2H, H$_b$-C(16), H$_a$-C(21)), 1.72 (dt, ^2J = 13.8 Hz, ^3J = 4.0 Hz, 1H, H$_b$-C(21)), 1.67 (dd, ^3J = 11.0, 5.7 Hz, 1H, H-C(9)), 1.62 – 1.44 (m, 5H, H$_2$-C(6), H$_a$-C(22), H$_a$-C(7), H$_b$-C(1)), 1.44 – 1.28 (m, 4H, H$_b$-C(22), H$_b$-C(7), H-C(5), H-C(19)), 1.12 (s, 3H, H$_3$-C(27)), 1.12 – 1.08 (m, 1H, H$_b$-C(15)), 1.08 (s, 6H, H$_3$-C(23), H$_3$-C(25)), 1.05 (s, 3H, H$_3$-C(24)), 1.03 – 0.99 (m, 1H, H-C(20)), 0.95 (d, ^3J = 6.2 Hz, 3H, H$_3$-C(30)), 0.89 (d, ^3J = 6.4 Hz, 3H, H$_3$-C(29)), 0.86 (s, 3H, H$_3$-C(26)). ^{13}C NMR (126 MHz, MeOD$_{d4}$) δ 220.64 (C3), 179.92 (d, ^3J = 8.8 Hz, (C28)), 139.68 (C13), 126.80 (C12), 62.93 (d, ^1J = 153.5 Hz, (C28')), 56.50 (C5), 54.32 (C18), 49.49 (C17), 48.15 (C9), 43.30 (C14), 40.80 (C8), 40.46 (C19), 40.41 (C1), 40.23 (C20), 37.82 (C10), 37.44 (C21), 35.14 (C7), 33.68 (C2), 31.84 (C22), 29.39 (C15), 27.07 (C23), 25.07 (C16), 24.51 (C11), 24.07 (C27), 21.92 (C24), 21.60 (C30), 20.72 (C6), 17.67 (C29), 17.63 (C26), 15.72 (C25). ^{31}P NMR (121 MHz, MeOD$_{d4}$) δ 11.53. HRMS: [$C_{31}H_{49}O_6P$-H^+] 547.3194; found 547.3195 (0.2 ppm). $[\alpha]_D^{20} = +0.49$; $[\alpha]_{546}^{20} = +0.58$; $[\alpha]_{436}^{20} = +0.97$; $[\alpha]_{405}^{20} = +1.23$; $[\alpha]_{365}^{20} = +1.62$ (c = 1.00, MeOH).

Sodium (3β-hydroxy-(17R)-17-28-norlup-20(29)-en)-2-oxoethyl-phosphonate 8a

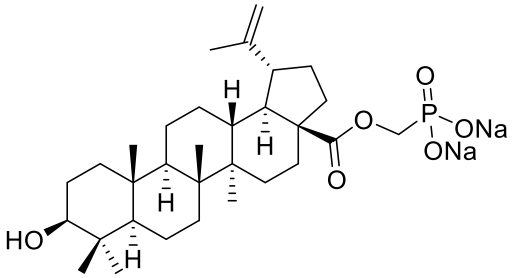

^{1}H NMR (500 MHz, MeOD$_{d4}$) δ 4.62 (s, 1H, H_a-C(29)), 4.48 (s, 1H, H_b-C(29)), 4.08 (dd, ^{2}J = 13.5, 8.9 Hz, 1H, H_a-C(28')), 3.95 (dd, ^{2}J = 13.5, 8.9 Hz, 1H, H_b-C(28')), 3.02 (dd, ^{3}J = 11.4, 4.6 Hz, 1H, H-C(3)), 2.93 (td, ^{3}J = 11.3, 4.5 Hz, 1H, H-C(19)), 2.32 (d, ^{2}J = 12.3 Hz, 1H, H_a-C(16)), 2.27 – 2.19 (m, 1H, H-C(13)), 1.99 (dd, ^{2}J = 12.1 Hz, ^{3}J = 8.1 Hz, 1H, H_a-C(21)), 1.88 – 1.80 (m, 1H, H_a-C(22)), 1.66 - 1.57 (m, 1H, H_a-C(12)), 1.59 (s, 3H, H_3-C(30)), 1.57 – 1.11 (m, 15H, H_2-C(6), H_2-C(11), H_2-C(7), H_2-C(2), H_b-C(22), H_b-C(21), H_b-C(16), H_a-C(1), H-C(9), H-C(18), H_a-C(15)), 1.11 – 0.91 (m, 2H, H_b-C(15), H_b-C(12)), 0.89 (s, 3H, H_3-C(27)), 0.87 – 0.82 (m, 7H, H_3-C(23), H-C(1), H_3-C(26)), 0.76 (s, 3H, H_3-C(25)), 0.65 (s, 3H, H_3-C(24)), 0.63 – 0.57 (m, 1H, H-C(5)). ^{13}C NMR (126 MHz, MeOD$_{d4}$) δ 177.50 (d, ^{3}J = 8.8 Hz, (C28)), 152.07 (C20), 110.11 (C29), 79.69 (C3), 61,13 (d, ^{1}J = 159.5 Hz, (C28')), 57.95 (C17), 56.90 (C5), 52.05 (C9), 50.81 (C18), 48.32 (C19), 43.52 (C14), 41.95 (C8), 40.11 (C4), 39.96 (C1), 39.42 (C13), 38.33 (C10), 37.83 (C21), 35.55 (C7), 32.99 (C16), 31.67 (C22), 30.95 (C15), 28.61 (C23), 28.05 (C2), 26.90 (C12), 22.08 (C11), 19.57 (C30), 19.45 (C6), 16.74 (C25), 16.62 (C26), 16.11 (C24), 15.11 (C27). ^{31}P NMR (121 MHz, MeOD$_{d4}$) δ 11.47. HRMS: [$C_{31}H_{51}O_6P$-H^+] 549.3350; found 549.3351 (0.2 ppm). $[\alpha]_D^{20} = -0.07$; $[\alpha]_{546}^{20} = -0.08$; $[\alpha]_{436}^{20} = -0.10$; $[\alpha]_{405}^{20} = -0.10$; $[\alpha]_{365}^{20} = -0.11$ (c = 1.00, MeOH).

Sodium (3β-hydroxy-(17R)-17-28-norurs-12(13)-en)-2-oxoethyl-phosphonate 8b

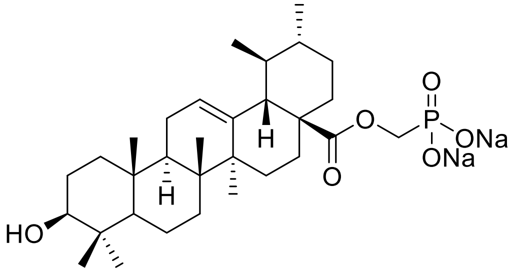

^{1}H NMR (500 MHz, MeOD*d4*) δ 5.27 (d, ^{3}J = 3.9 Hz, 1H, H-C(13)), 4.06 (dd, ^{2}J = 10.3, 5.1 Hz, 1H, H_a-C(28')), 4.02 (dd, ^{2}J = 10.3, 5.1 Hz, 1H, H_b-C(28')), 3.15 (dd, ^{3}J = 11.4, 4.6 Hz, 1H, H, H-C(3)), 2.31 (d, ^{3}J = 11.3 Hz, 1H, H-C(18)), 2.04 (td, ^{2}J = 13.3 Hz, ^{3}J = 4.3 Hz, 1H, H_a-C(16)), 1.96 – 1.85 (m, 3H, H_2-C(11), H_a-C(15)), 1.84 – 1.73 (m, 2H, H_b-C(16), H_a-C(21)), 1.73 – 1.61 (m, 3H, H_a-C(2), H_b-C(21), H_a-C(1)), 1.61 – 1.46 (m, 5H, H_a-C(6), H_b-C(2), H_a-C(22), H_a-C(7), H-C(9)), 1.45 – 1.26 (m, 4H, H_b-C(6), H_b-C(22), H_b-C(7), H-C(19)), 1.11 (s, 3H, H_3-C(27)), 1.07 (d, ^{2}J = 13.4 Hz, 1H, H_b-C(15)), 1.04 – 0.98 (m, 2H, H-C(20), H_b-C(1)), 0.97 (s, 3H, H_3-C(23)), 0.96 (d, ^{3}J = 6.3 Hz, 3H, H_3-C(30)), 0.95 (s, 3H, H_3-C(25)), 0.89 (d, ^{3}J = 6.4 Hz, 3H, H_3-C(25)), 0.79 (s, 3H, H_3-C(24)), 0.78 (s, 3H, H_3-C(26)), 0.74 (d, ^{3}J = 11.4 Hz, 1H, H-C(5)). ^{13}C NMR (126 MHz, MeOD*d4*) δ 179.47 (d, ^{3}J = 9.2 Hz, C(28)), 139.44 (C13), 127.13 (C12), 79.72 (C3), 61.63 (d, ^{1}J = 158.8 Hz, C(28')), 56.75 (C5), 54.19 (C18), 49.49 (C17), 49.03 (C9), 43.12 (C14), 40.83 (C8), 40.39 (C19), 40.21 (C20), 40.01 (C1), 39.84 (C4), 38.09 (C10), 37.51 (C21), 34.20 (C7), 31.77 (C22), 29.29 (C15), 28.76 (C23), 27.90 (C2), 25.09 (C16), 24.36 (C11), 24.18 (C27), 21.59 (C30), 19.47 (C6), 17.70 (C24), 17.62 (C29), 16.37 (C26), 16.03 (C25). ^{31}P NMR (121 MHz, MeOD*d4*) δ 12.17. HRMS: [$C_{31}H_{51}O_6P$-H^+] 549.3350; found 549.3349 (0.2 ppm). $[\alpha]_D^{20} = +0.33$; $[\alpha]_{546}^{20} = +0.40$; $[\alpha]_{436}^{20} = +0.71$; $[\alpha]_{405}^{20} = +0.88$; $[\alpha]_{365}^{20} = +1.12$ (c = 1.00, MeOH).

Sodium (3β-hydroxy-(17R)-17-28-norolean-12(13)-en)-2-oxoethyl-phosphonate 8c

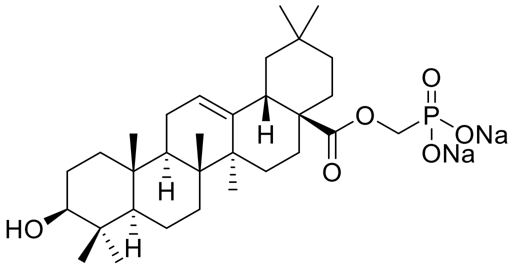

^{1}H NMR (500 MHz, MeOD$_{d4}$) δ 5.25 (t, ^3J = 3.6 Hz, 1H, H-C(13)), 4.16 (dd, ^2J = 13.2, 8.8 Hz, 1H, H$_a$-C(28')), 3.96 (dd, ^2J = 13.3, 8.6 Hz, 1H, H$_b$-C(28')), 3.14 (dd, ^3J = 11.4, 4.5 Hz, 1H, C(3)), 2.88 (dd, ^3J = 14.2, 4.4 Hz, 1H, H-C(18)), 1.99 (dt, ^2J = 13.8 Hz, ^3J = 7,7 Hz, 1H, H$_a$-C(16)), 1.96 – 1.79 (m, 3H, H$_b$-C(16), H-C(11)), 1.79 – 1.27 (m, 13H, H$_2$-C(6), H$_2$-C(2), H$_a$-C(15), H$_2$-C(22), H$_2$-C(7), H$_a$-C(21), H$_a$-C(1), H$_a$-C(19), H-C(9)), 1.19 (d, ^2J = 14.9 Hz, 1H, H$_b$-C(21)), 1.15 (s, 3H, H$_3$-C(27)), 1.13 – 1.00 (m, 2H, H$_b$-C(19), H$_b$-C(15)), 1.00 – 0.95 (m, 4H, H$_3$-C(23), H$_b$-C(1)), 0.94 (s, 6H, H$_3$-C(29), H$_3$-C(25)), 0.90 (s, 3H, H$_3$-C(30)), 0.78 (s, 6H, H$_3$-C(26), H$_3$-C(24)), 0.77 – 0.72 (m, 1H, H-C(5)). ^{13}C NMR (126 MHz, MeOD$_{d4}$) δ 180.03 (d, ^3J = 8.9 Hz, C(C28)), 145.27 (C12), 123.59 (C13), 79.77 (C3), 62.76 (d, ^1J = 154.7 Hz, (C28')), 56.80 (C5), 49.17 (C9), 48.11 (C17), 47.34 (C19), 42.85 (C18), 40.60 (C14), 39.88 (C8), 39.85 (C1), 38.17 (C4), 38.17 (C10), 34.99 (C21), 33.94 (C7), 33.64 (C30), 33.17 (C22), 31.58 (C20), 29.11 (C15), 28.74 (C23), 27.88 (C2), 26.41 (C27), 24.53 (C11), 24.16 (C29), 23.80 (C16), 19.50 (C6), 17.67 (C26), 16.30 (C24), 15.91 (C25). ^{31}P NMR (121 MHz, MeOD$_{d4}$) δ 11.59. HRMS: [$C_{31}H_{51}O_6P$-H^+] 549.3350; found 549.3348 (0.4 ppm). $[\alpha]_D^{20} = +0.31$; $[\alpha]_{546}^{20} = +0.43$; $[\alpha]_{436}^{20} = +0.76$; $[\alpha]_{405}^{20} = +0.91$; $[\alpha]_{365}^{20} = +1.21$ (c = 1.00, MeOH).

Synthesis of a mixture of products 9, 10 and 11

To solution of betulin (**1a**) (500 mg, 1.131 mmol, 1 eq.) in anhydrous THF (5mL) freshly prepared 1M LDA solution in THF (2.37 mL, 2.37 mmol, 2.1 eq.) is added dropwise at -78 °C. The resulting reaction mixture is warmed up to 0 °C and stirred at ambient temperature for 40 min. Then the solution of previously prepared (dimethoxyphosphoryl)methyl trifluoromethanesulfonate (712 mg, 2.49 mmol, 2.2 eq.) in anhydrous THF (3 mL) is added dropwise to the suspension of lithium alkoxide at 0 °C. The obtained reaction mixture is warmed up to room temperature and stirred for 3 h. Then the reaction mixture is quenched by MeOH (2 mL), evaporated to dryness, redissolved in EtOAc (50 mL) and subsequently washed with H_2O (30 mL) and brine (2 x 30 mL). The combined organic layer is dried over anhydrous Na_2SO_4. After filtration, the filtrate is concentrated *in vacuo* and purified by silica column chromatography (Hexanes-EtOAc 4:1 - 1:9) to yield bis-ether **9** as a white amorphous solid (29%, 226 mg). R*f* = 0.31 (100% EtOAc). Side product **11** was isolated with preparative HPLC on C18 reverse phase column by gradient A/B (60/40) → A/B 0/100)*. However, the presence of monoester **10** was detected by HPLC and NMR, yet the product 10 was not isolated in pure form.

* A: 95 parts of 0.1% aqueous solution of trifluoroacetic acid and 5 parts of acetonitrile;
B: acetonitrile.

(3S)-3,28-di((dimethoxyphosphoryl)methyloxy)-lup-20(29)ene 9

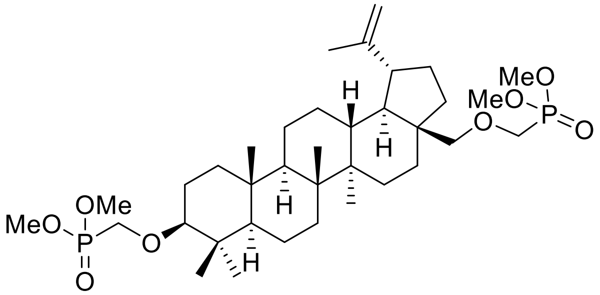

^{1}H NMR (500 MHz, $CDCl_3$) δ 4.67 (s, 1H, H_a-C(29)), 4.57 (s, 1H, H_b-C(29)), 3.99 (dd, ^2J = 13.6 Hz, ^3J = 8.7 Hz, 1H, H_a-C(28')), 3.87 – 3.76 (m, 14H, H_2-C(3'), (H_3-CO)$_4$), 3.70 (d, ^2J = 8.7 Hz, 1H, H_a-C(28)), 3.68 (dd, ^2J = 13.6 Hz, ^3J = 9.7 Hz, 1H, H_b-C(28')), 3.24 (d, ^2J = 8.7 Hz, 1H, H_b-C(28)), 2.83 (dd, ^3J = 11.8, 4.3 Hz, 1H, H-C(3)), 2.38 (td, ^3J = 10.8, 5.5 Hz, 1H, H-C(19)), 2.01 – 1.86 (m, 3H, H_a-C(16), H_a-C(21), H_a-C(22)), 1.78 – 1.68 (m, 3H, H_a-C(1), H_a-C(15), H_a-C(2)),

1.67 (s, 3H, H_3-C(30)), 1.65 – 1.57 (m, 2H, H_a-C(12), H-C(13)), 1.56 – 1.43 (m, 3H, H_a-C(6), H_b-C(2), H-C(18)), 1.43 – 1.33 (m, 5H, H_a-C(11), H_b-C(6), H_b-C(16), H_2-C(7)), 1.28 – 1.13 (m, 3H, H_b-C(11), H_b-C(22), H-C(9)), 1.08 – 0.99 (m, 6H, H_3-C(26), H_b-C(12), H_b-C(15), H_b-C(21)), 0.98 (s, 3H, H_3-C(23)), 0.95 (s, 3H, H_3-C(27)), 0.85 – 0.78 (m, 4H, H_3-C(25)), 0.76 (s, 3H, H_3-C(26)), 0.67 (d, ^3J = 9.5 Hz, 1H, H-C(5)). ^{13}C NMR (126 MHz, $CDCl_3$) δ 150.61 (C20), 109.82 (C29), 90.08 (d, ^3J = 12.2 Hz, (C3)), 72.41 (d, ^3J = 9.5 Hz (C28)), 65.40 (d, ^1J =164.5 Hz, (C3')), 63.18 (d, ^1J = 166.5 Hz, (C28')), 55.81 (C5), 53.29 (d, ^2J = 6.6 Hz, (MeO)) 53.14 (d, ^2J = 6.8 Hz, (MeO)), 53.12 (d, ^2J = 6.5 Hz, (MeO)), 53.09 (d, ^2J = 6.9 Hz, (MeO)), 50.46 (C9), 48.98 (C18), 48.03 (C19), 47.56 (C17), 42.81 (C14), 41.06 (C8), 39.10 (C4), 38.52 (C1), 37.66 (C13), 37.26 (C10), 34.65 (C21), 34.31 (C7), 29.96 (C22), 29.86 (C16), 28.15 (C23), 27.20 (C15), 25.30 (C12), 22.35 (C2), 20.97 (C11), 19.25 (C30), 18.27 (C6), 16.25 (C24), 16.20 (C25), 16.10 (C26), 14.89 (C27). ^{31}P NMR (121 MHz, $CDCl_3$) δ 23.96, 23.73. HRMS: [$C_{36}H_{64}O_8P_2$+H^+] 687.4149; found 687.4141 (1.1 ppm). $[\alpha]_D^{20} = +0.21$; $[\alpha]_{546}^{20} = +0.26$; $[\alpha]_{436}^{20} = +0.47$; $[\alpha]_{405}^{20} = +0.57$; $[\alpha]_{365}^{20} = +0.74$ (c = 1.00, MeOH).

(((3S)-3-((dimethoxyphosphoryl)methyloxy)-28-lup-20(29)enyloxy)(methoxy)phosphoryl)methyl trifluoromethanesulfonate 11

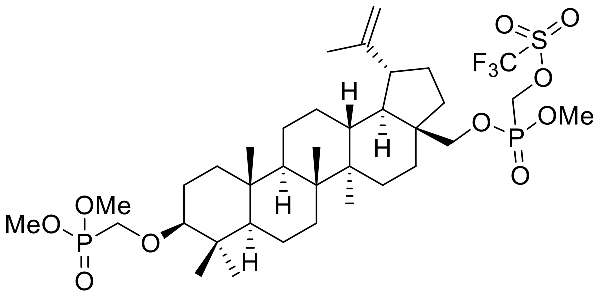

^{1}H NMR (500 MHz, $CDCl_3$) δ 4.68 (s, 1H, H_a-C(29)), 4.59 (s, 1H, H_b-C(29)), 4.27 (dd, ^2J = 9.3 Hz, ^3J = 5.2 Hz, 1H, H_a-C(28)), 4.00 (dd, ^2J = 13.7 Hz, ^3J = 8.8 Hz, 1H, H-C(3)), 3.88 – 3.78 (m, 9H, $(H_3\text{-COP})_2$, H_2-C(28'), H_b-C(28)), 3.70 (dd, ^2J = 13.7 Hz, ^3J = 9.7 Hz, 1H, H-C(3')), 3.47 (m, 3H, OMe), 2.83 (dd, ^3J = 11.8, 4.3 Hz, 1H, H-C(3)), 2.38 (ddd, ^3J = 10.6, 10.1, 5.6 Hz, 1H, H-C(19)), 2.01 – 1.86 (m, 3H, H_a-C(16), H_a-C(21), H_a-C(22)), 1.80 – 1.69 (m, 3H, H_a-C(2), H_a-C(15), H_a-C(1)), 1.66 (s, 3H, H_3-C(30)), 1.66 – 1.57 (m, 3H, H_a-C(12), H-C(13), H-C(18)), 1.57 – 1.32 (m, 7H, H_2-C(6), H_a-C(11), H_b-C(2), H_b-C(22), H-C(7)), 1.32 – 1.13 (m, 3H, H_b-C(11), H_b-

C(16)), 1.13 – 1.03 (m, 3H, H_b-C(12), H_b-C(15), H_b-C(22)), 1.02 (s, 3H, H_3-C(26)), 0.98 (s, 3H, H_3-C(23)), 0.96 (s, 3H, H_3-C(27)), 0.82 (s, 3H, H_3-C(25)), 0.82 – 0.75 (m, 1H, H_b-C(1)), 0.75 (s, 3H, H_3-C(24)), 0.67 (d, ^3J = 9.6 Hz, 1H, H-C(5)). ^{13}C NMR (126 MHz, $CDCl_3$) δ 150.13 (C20), 110.09 (C29), 90.11 (d, ^3J = 12.4 Hz, (H-C(3)), 66.33 (d, ^1J = 166.44, H-C(3')), 65.34 (d,^2J = 7.6 Hz, C(28)), 63.10 (d, ^1J = 167.3 Hz, H-C(28')), 61.56 (d, ^2J = 13.1 Hz, MeO-P(H_2C(28))), 55.81 (C5), 53.40 (d, ^2J = 6.5 Hz, (MeO)) , 53.37 (d, ^2J = 6.6 Hz, (MeO)), 50.44 (C9), 48.76 (C18), 47.82 (C19), 47.37 (d, ^3J = 6.6 Hz, (C17)), 42.84 (C14), 41.04 (C8), 39.10 (C4), 38.52 (C1), 37.75 (C13), 37.26 (C10), 34.27 (C21), 34.20 (C7), 29.59 (C22), 29.30 (C16), 28.13 (C23), 26.95 (C15), 25.30 (C12), 22.33 (C2), 20.94 (C11), 19.24 (C30), 18.25 (C6), 16.23 (C24), 16.19 (C25), 16.08 (C26), 14.87 (C27). ^{31}P NMR (121 MHz, $CDCl_3$) δ 24.02, 22.69. R*f* = 0.43 (100% EtOAc).

Sodium (lup-20(29)-en-(3S)-3,28-diylbis(oxymethylene))bis(phosphonate) 12, demethylation process 9 → 12

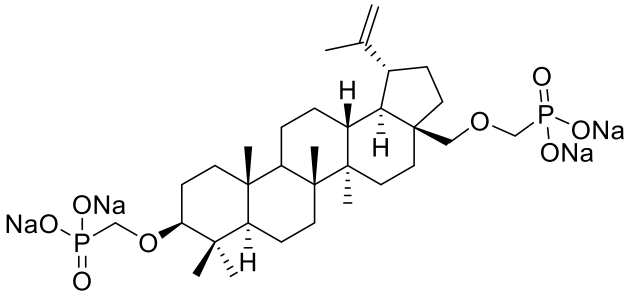

To a solution of compound **9** (275 mg, 0.4 mmol, 1 eq.) in anhydrous DCM (5 mL) TMSI (342 µL 2.4 mmol, 6 eq.) is added dropwise at -40 °C and the resulting reaction mixture is stirred at -40 °C for 5 h. Then MeOH (2 mL) is added dropwise at -40 °C. The obtained reaction mixture is stirred for additional 30 min and solution of $NaHCO_3$ (202 mg, 2.4 mmol, 6 eq.) in H_2O (6 mL) is added dropwise at -40 °C, and the resulting mixture is warmed up to room temperature. The resulting reaction mixture is warmed up to room temperature and the organic solvents are evaporated *in vacuo*. The obtained aqueous suspension is centrifuged and the supernatant is removed and discarded. The precipitate is re-suspended in deionized water (1 mL) and the centrifugation – supernatant removal procedure is repeated additional two times (in total: washing with water 3 × 1mL). The obtained precipitate is then dried in vacuo to yield product **12** as a yellowish amorphous solid (78%, 225 mg).

^{1}H NMR (500 MHz, MeOD) δ 4.69 (s, 1H, H_a-C(29)), 4.57 (s, 1H, H_b-C(29)), 3.85 (dd, ^{2}J = 13.3, 9.1 Hz, 1H, H_a-C(28’), 3.74 – 3.68 (m, 3H, H_a-C(28), H_2-C(3’)), 3.55 (dd, ^{2}J = 13.3, 10.2 Hz, 1H, H_b-C(28’)), 3.32(d, ^{2}J = 11.4 Hz, 1H, H_b-C(28)), 2.89 (dd, ^{3}J = 11.7, 4.3 Hz, 1H, H-C(3)), 2.46 (td, ^{3}J = 11.0, 5.9 Hz, 1H, H-C(19)), 2.08 – 1.98 (m, 3H, H, H_a-C(16), H_a-C(21), H_a-C(22)), 1.90 – 1.70 (m, 5H, H_a-C(15), H_a-C(1), H-C(13), H_a-C(12), H_a-C(2)), 1.69 (s, 3H, H_3-C(30)), 1.62 – 1.39 (m, 7H, H_2-C(6), H_a-C(11), H_b-C(2), H-C(18), H_2-C(7)), 1.39 – 1.10 (m, 4H, H_b-C(11), H_b-C(22), H_b-C(16), H-C(9)), 1.09 (s, 3H, H_3-C(26)), 1.09 – 1.03 (m, 1H, H_b-C(12)), 1.03 (s, 3H, H_3-C(23)), 1.02 – 0.95 (m, 5H, H_3-C(27), H_b-C(21), H_b-C(15)), 0.95 – 0.89 (m, 1H, H_b-C(1)), 0.88 (s, 3H, H_3-C(25)), 0.80 (s, 3H, H_3-C(24)), 0.74 (d, ^{3}J = 9.6 Hz, 1H, H-C(5)). ^{13}C NMR (126 MHz, MeOD) δ 150.51 (C20), 108.83 (C10), 89.45 (d,^{3}J = 12.1 Hz, C(3)), 71.56 (d, ^{3}J = 10.6 Hz, (C28)), 66.93 (d, ^{1}J = 162.8 Hz, (C3’)), 64.67 (d, ^{1}J = 164.8 Hz, C(28’)), 55.77 (C5), 50.41 (C9), 48.79 (C18), 48.01 (C19), 42.40 (C14) , 40.77 (C8), 38.69 (C1), 38.33 (C4), 37.51 (C13), 36.90 (C10), 34.30 (C21), 34.05 (C7), 29.58 (C22), 29.49 (C16), 27.19 (C23), 26.97 (C15), 25.20 (C12), 21.92 (C2), 20.60 (C11), 17.98 (C30), 17.89 (C6), 15.34 (C25), 15.32 (C24), 15.22 (C26), 13.85 (C27). ^{31}P NMR (121 MHz, MeOD) δ 19.72, 19.07. $[\alpha]_D^{20} = +0.11$; $[\alpha]_{546}^{20} = +0.17$; $[\alpha]_{436}^{20} = +0.33$; $[\alpha]_{405}^{20} = +0.37$; $[\alpha]_{365}^{20} = +0.46$ (c = 1.00, MeOH).

NMR SPECTRA

^{1}H NMR (500 MHz, $CDCl_3$) spectrum of 3-oxo-(17S)-17-(((dimethoxyphosphoryl)methoxy)carbonyl)-28-norlup-20(29)ene 3a

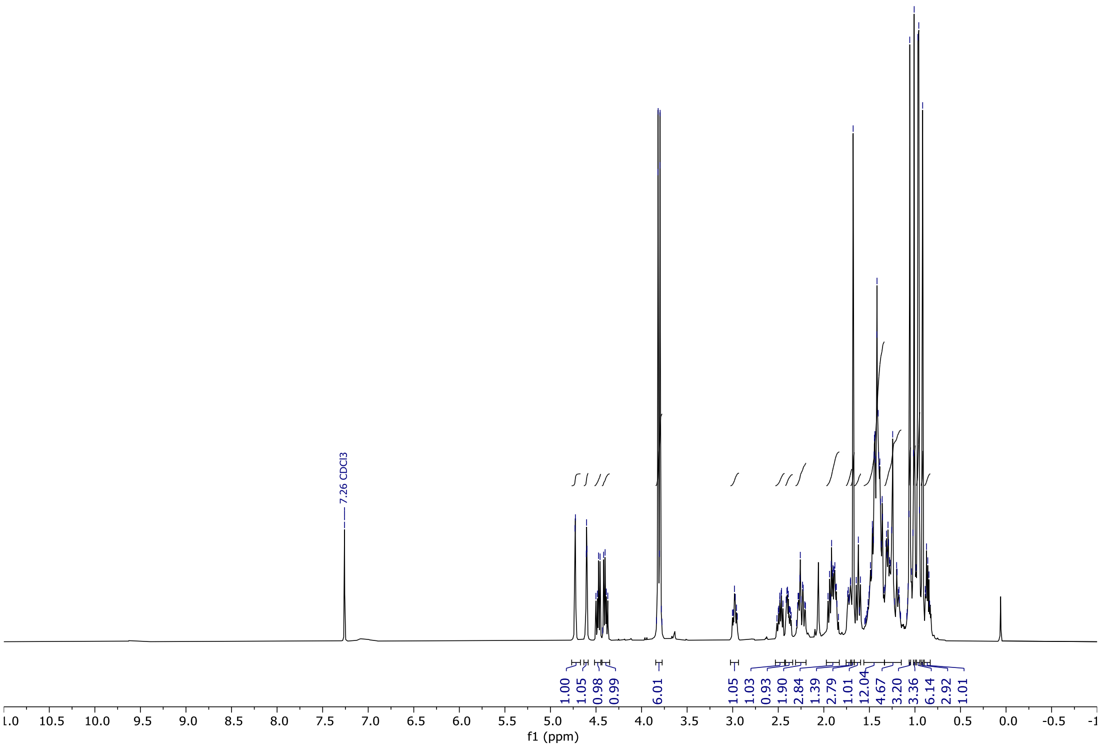

^{13}C NMR (126 MHz, $CDCl_3$) spectrum of 3-oxo-(17S)-17-(((dimethoxyphosphoryl)methoxy)carbonyl)-28-norlup-20(29)ene 3a

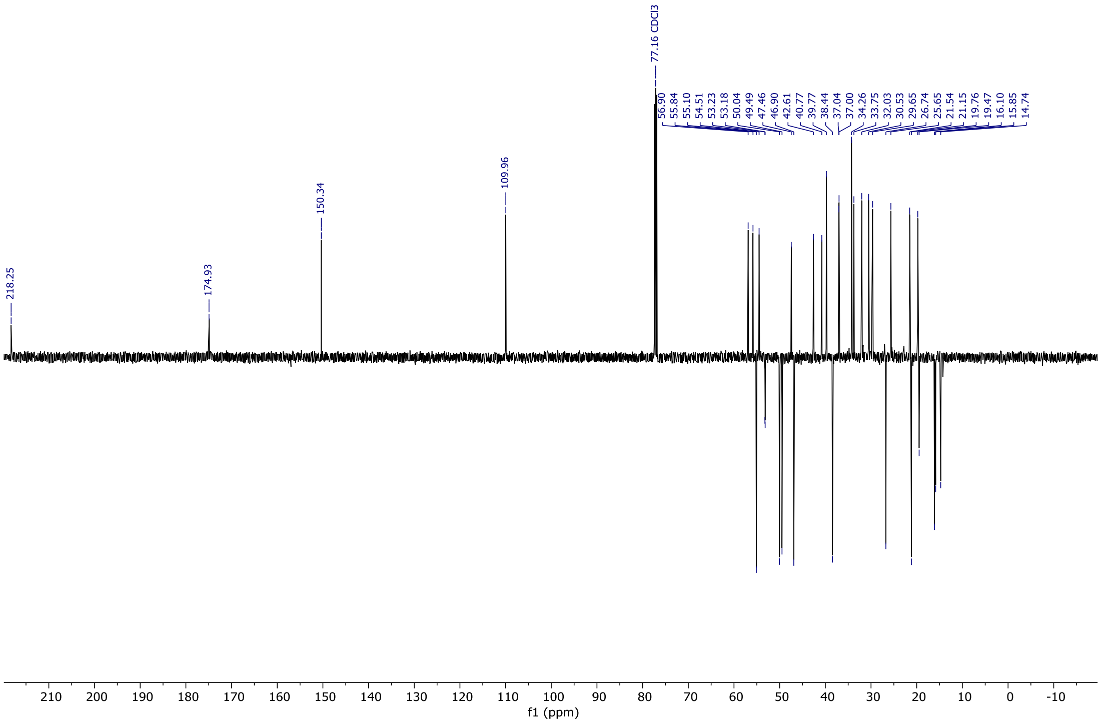

^{1}H NMR (500 MHz, $CDCl_3$) spectrum of 3-oxo-(17S)-17-(((dimethoxyphosphoryl)methoxy)carbonyl)-28-norolean-12(13)-ene 3b

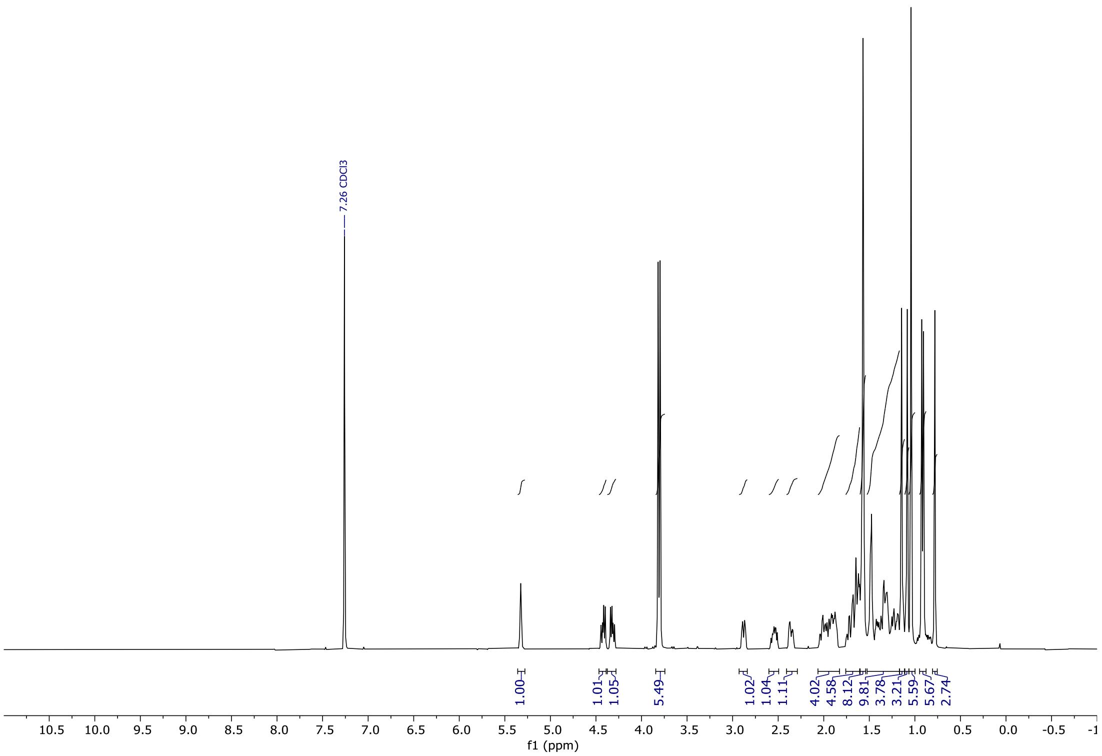

^{13}C NMR (126 MHz, $CDCl_3$) spectrum of 3-oxo-(17S)-17-(((dimethoxyphosphoryl)methoxy)carbonyl)-28-norolean-12(13)-ene 3b

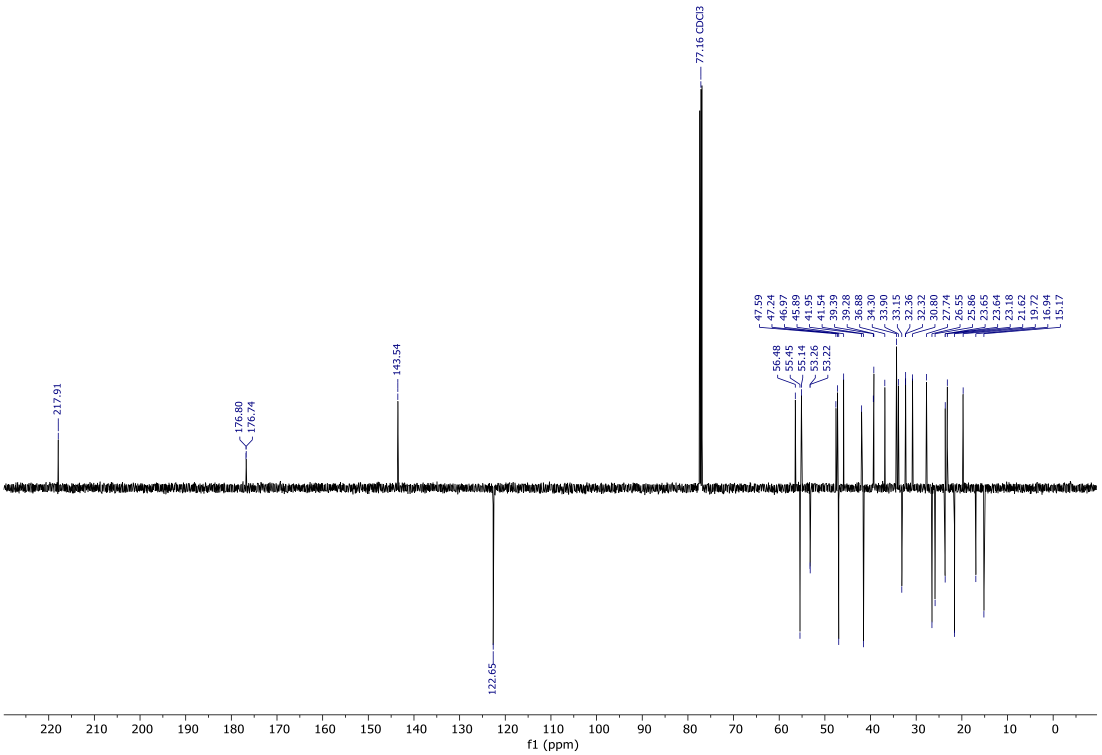

^{1}H NMR (500 MHz, $CDCl_3$) spectrum of 3-oxo-(17S)-17-(((dimethoxyphosphoryl)methoxy)carbonyl)-28-norurs-12(13)-ene 3c

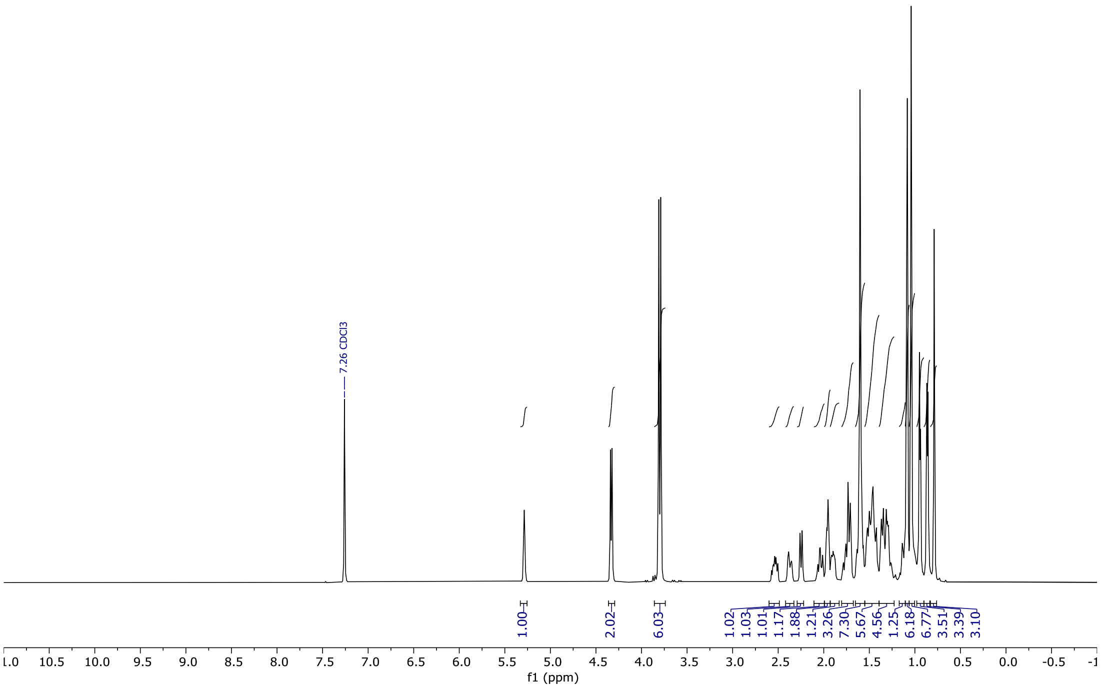

^{13}C NMR (126 MHz, $CDCl_3$) spectrum of 3-oxo-(17S)-17-(((dimethoxyphosphoryl)methoxy)carbonyl)-28-norurs-12(13)-ene 3c

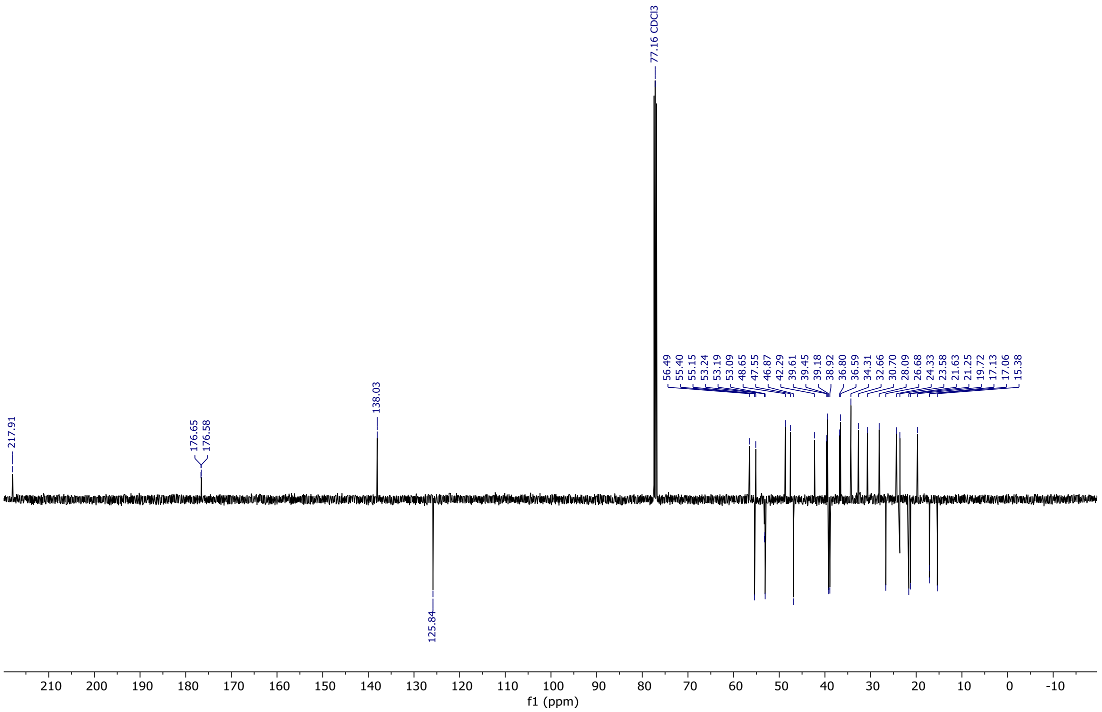

^{1}H NMR (500 MHz, CDCl$_3$) spectrum of (17S)-17-(((dimethoxyphosphoryl)methoxy)carbonyl)-3β-hydroxy-28-norlup-20(29)ene 4a

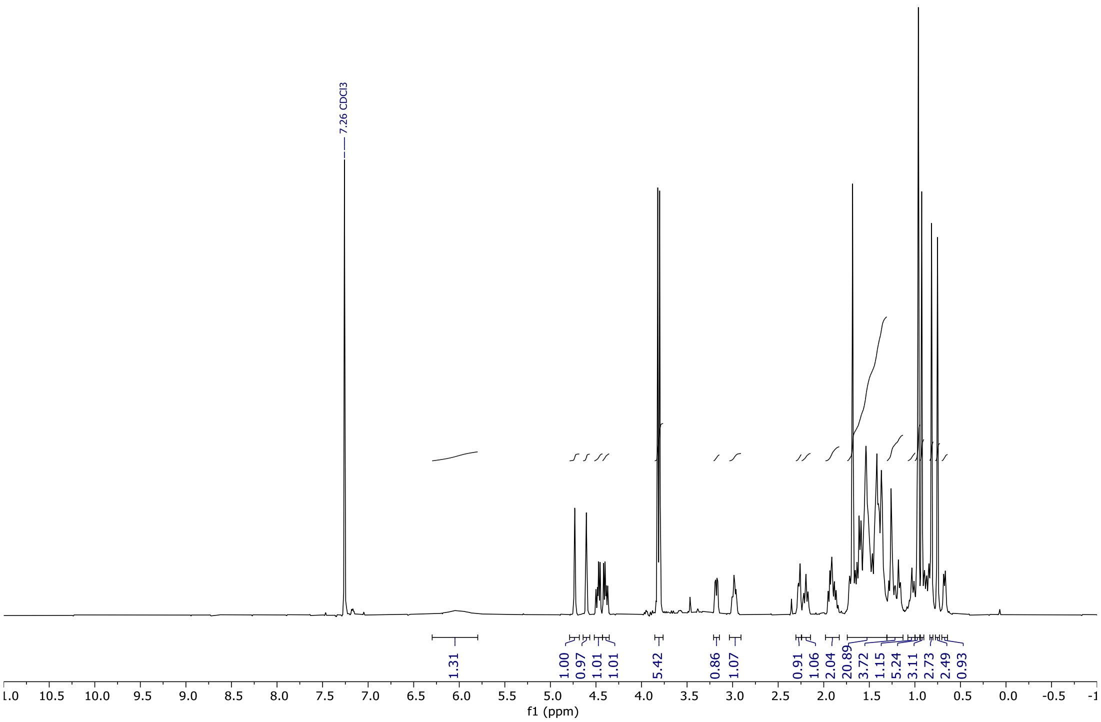

^{13}C NMR (126 MHz, CDCl$_3$) spectrum of (17S)-17-(((dimethoxyphosphoryl)methoxy)carbonyl)-3β-hydroxy-28-norlup-20(29)ene 4a

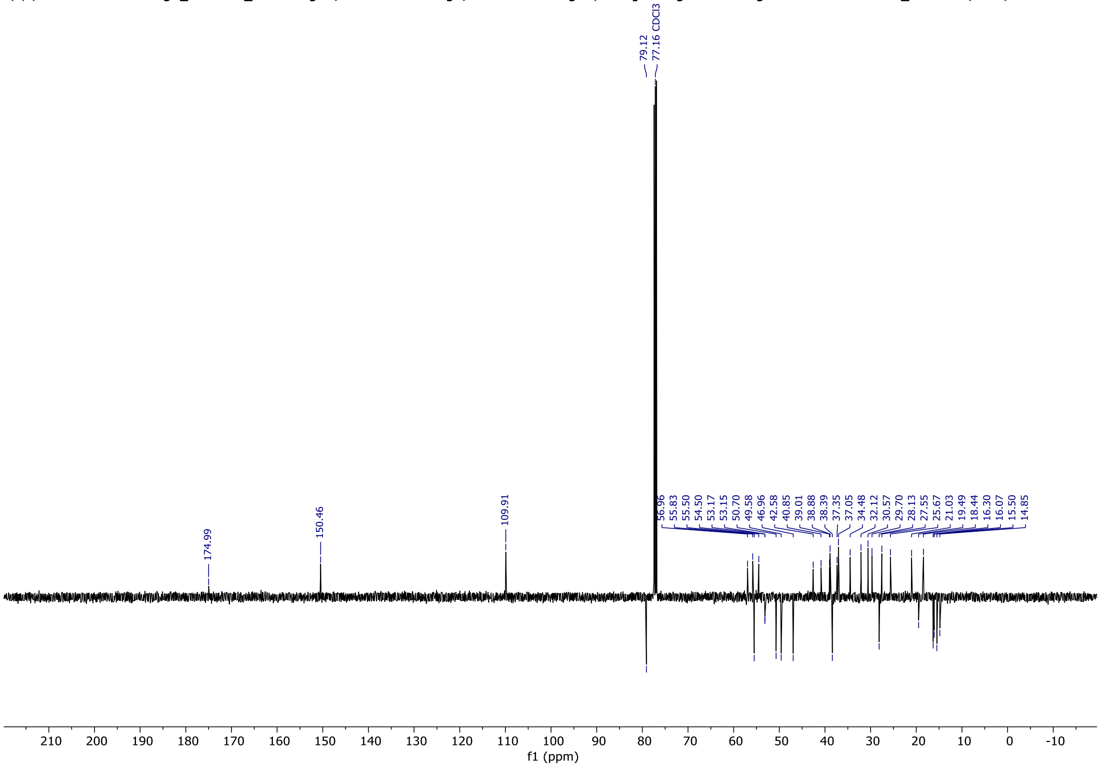

^{1}H NMR (500 MHz, $CDCl_3$) spectrum of (17S)-17-(((dimethoxyphosphoryl)methoxy)carbonyl)-3β-hydroxy -28-norolean-12(13)-ene 4b

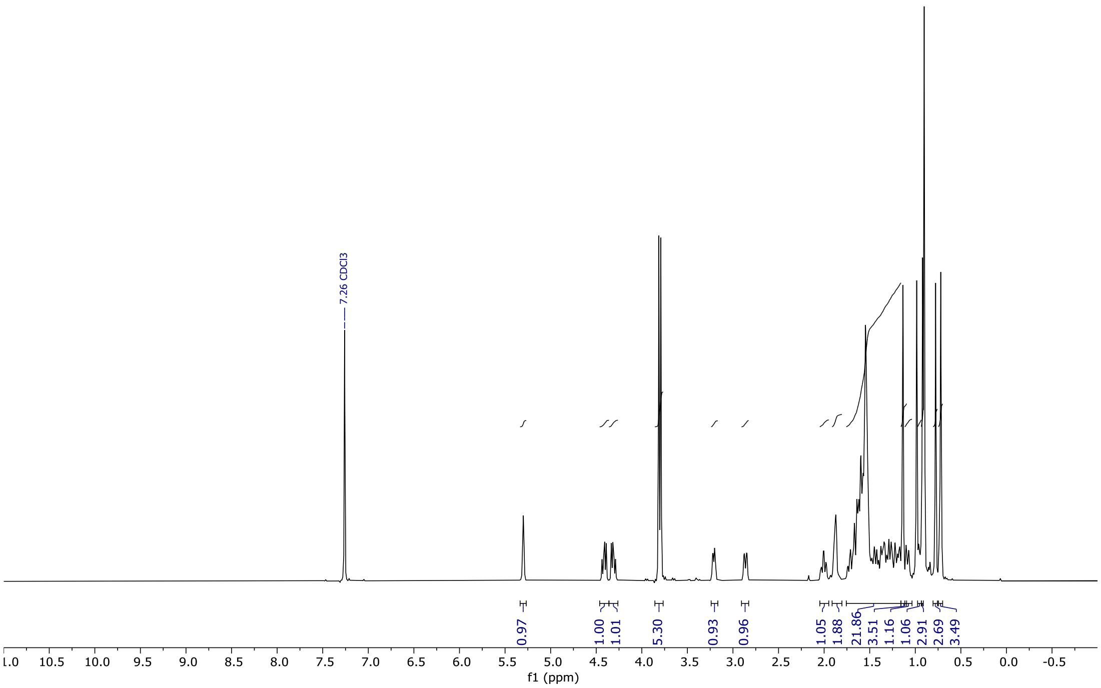

^{13}C NMR (126 MHz, $CDCl_3$) spectrum of (17S)-17-(((dimethoxyphosphoryl)methoxy)carbonyl)-3β-hydroxy -28-norolean-12(13)-ene 4b

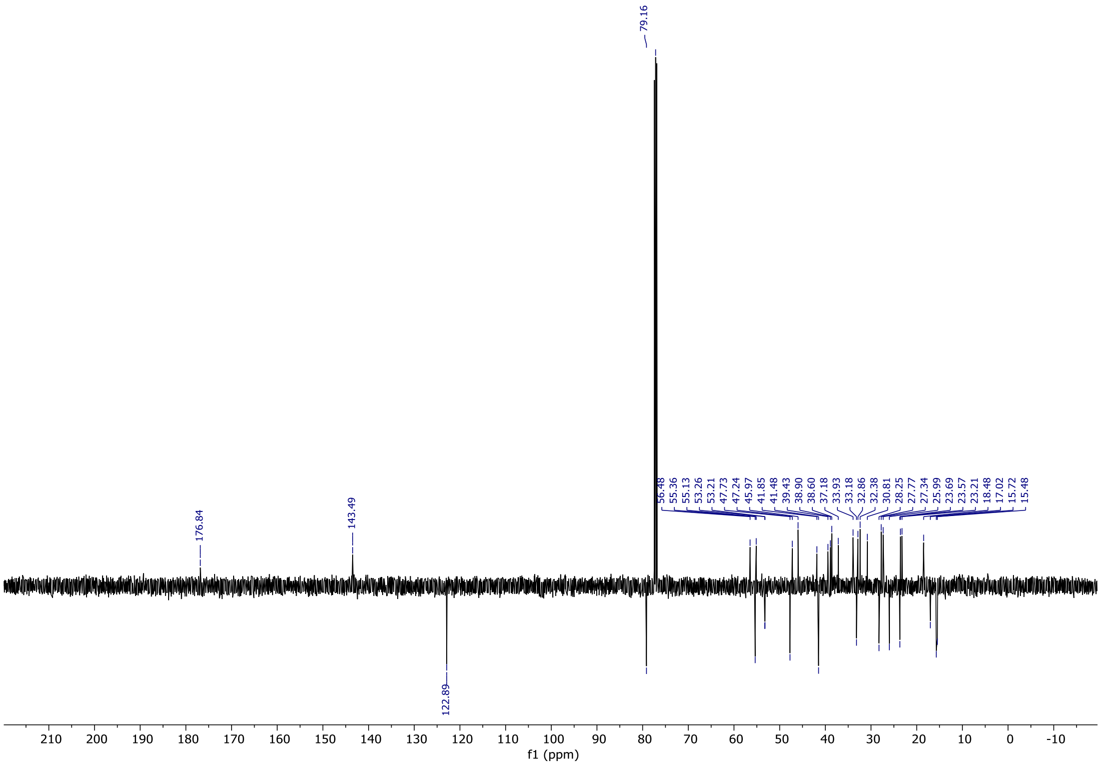

^{1}H NMR (500 MHz, $CDCl_3$) spectrum of (17S)-17-(((dimethoxyphosphoryl)methoxy)carbonyl)-3β-hydroxy -28-norurs-12(13)-ene 4c

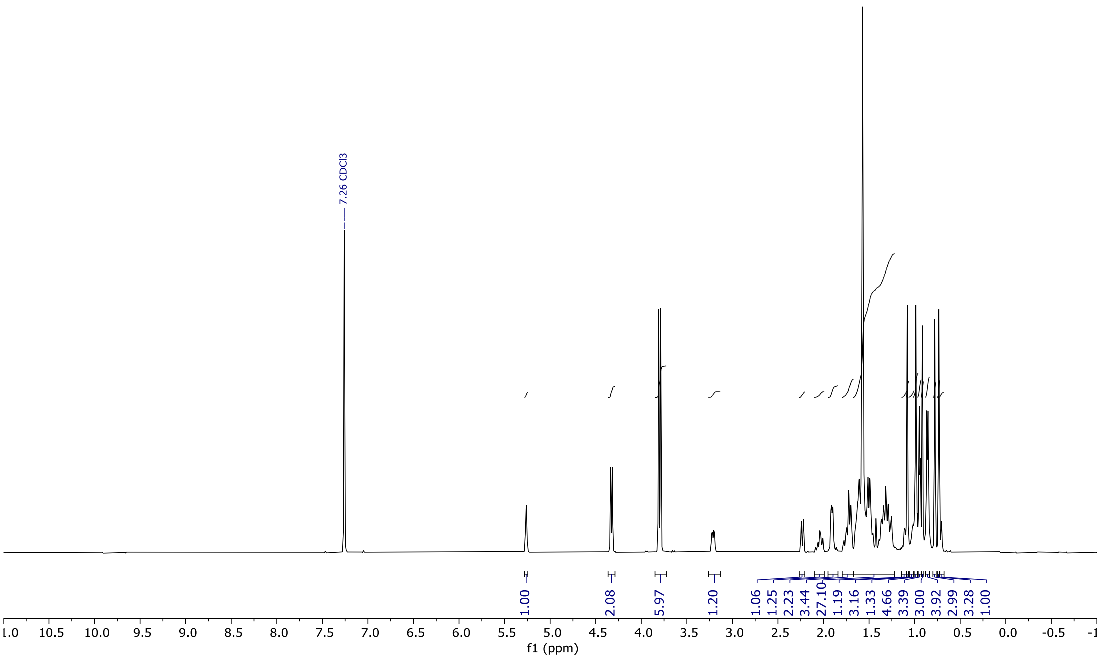

^{13}C NMR (126 MHz, $CDCl_3$) spectrum of (17S)-17-(((dimethoxyphosphoryl)methoxy)carbonyl)-3β-hydroxy -28-norurs-12(13)-ene 4c

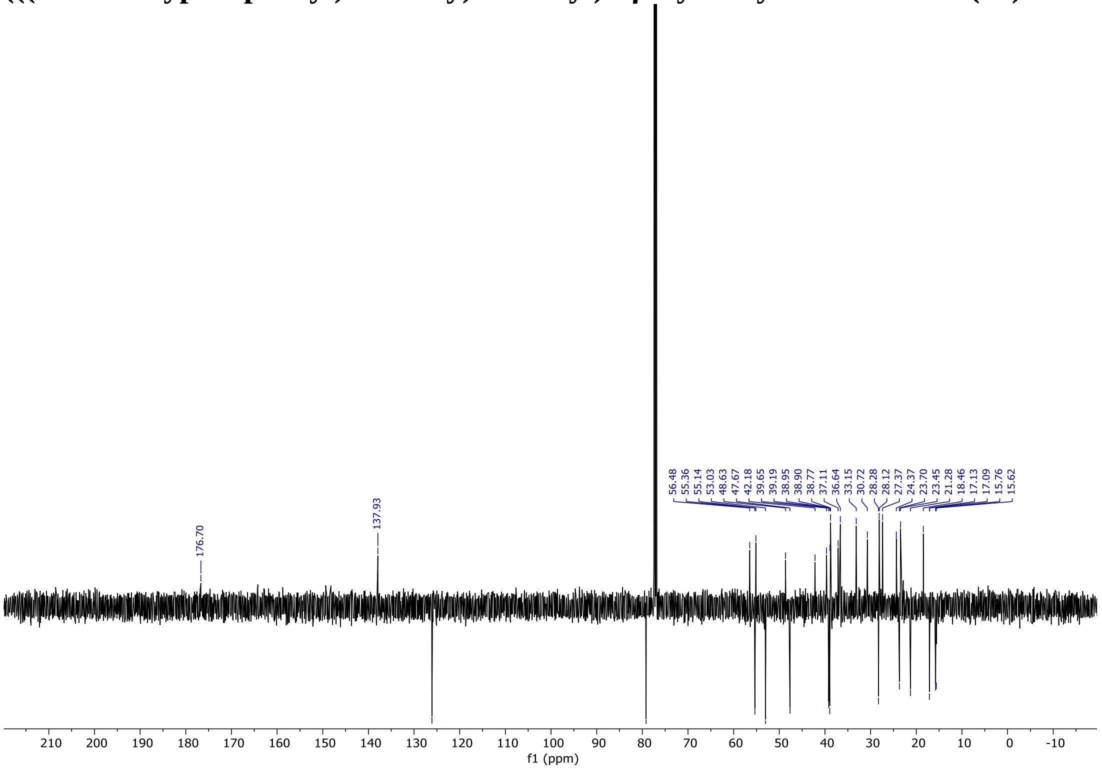

^{1}H NMR (500 MHz, MeOD$_{d4}$)) spectrum of sodium (3-oxo-(17R)-17-28-norlup-20(29)-en)-2-oxoethyl-phosphonate 7a

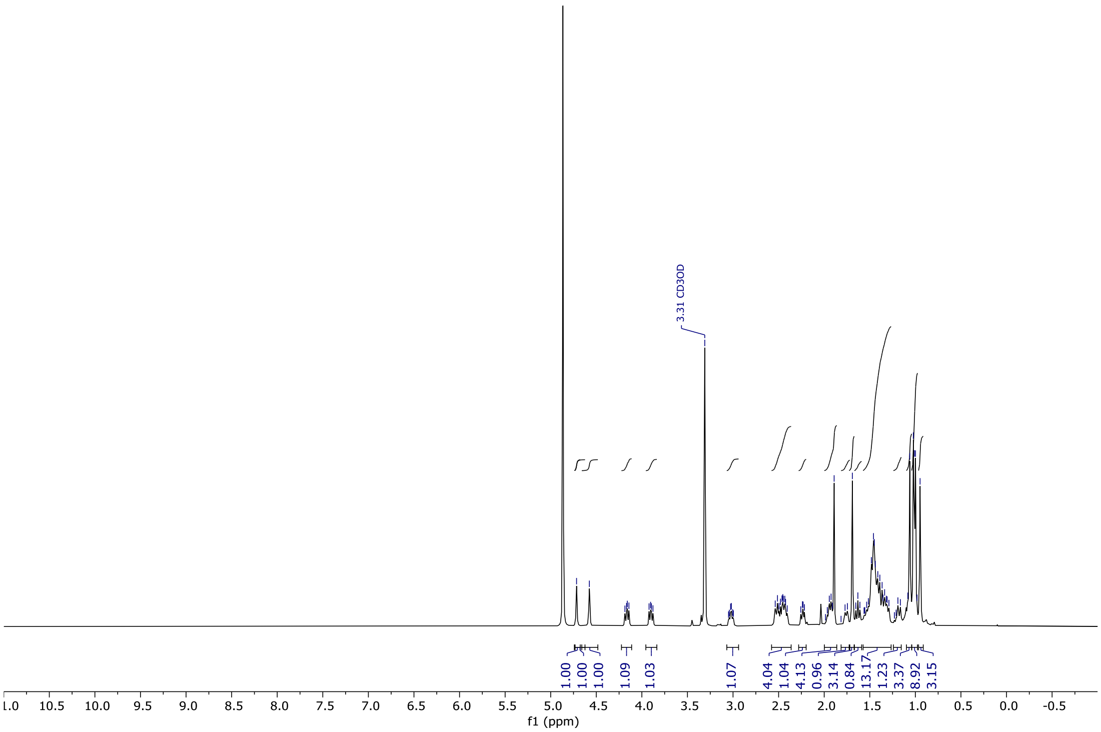

^{13}C NMR (126 MHz, MeOD$_{d4}$) spectrum of sodium (3-oxo-(17R)-17-28-norlup-20(29)-en)-2-oxoethyl-phosphonate 7a

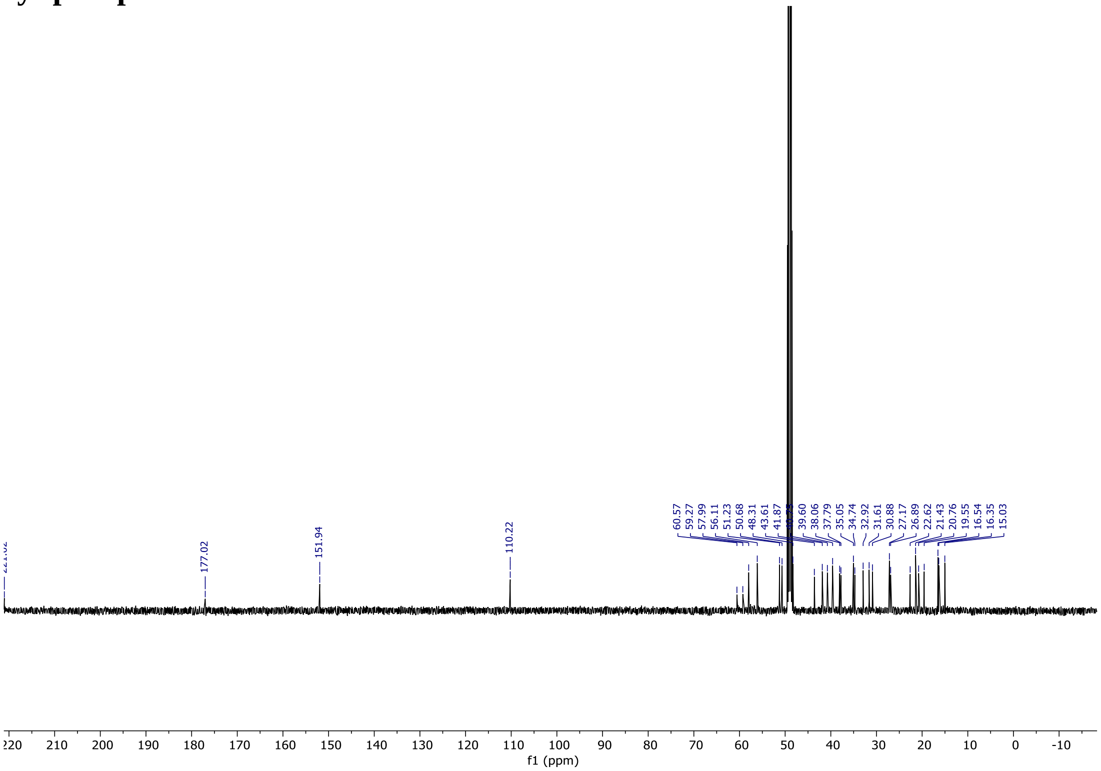

^{1}H NMR (500 MHz, MeOD$_{d4}$) spectrum of sodium (3-oxo-(17R)-17-28-norolean-12(13)-en)-2-oxoethyl-phosphonate 7b

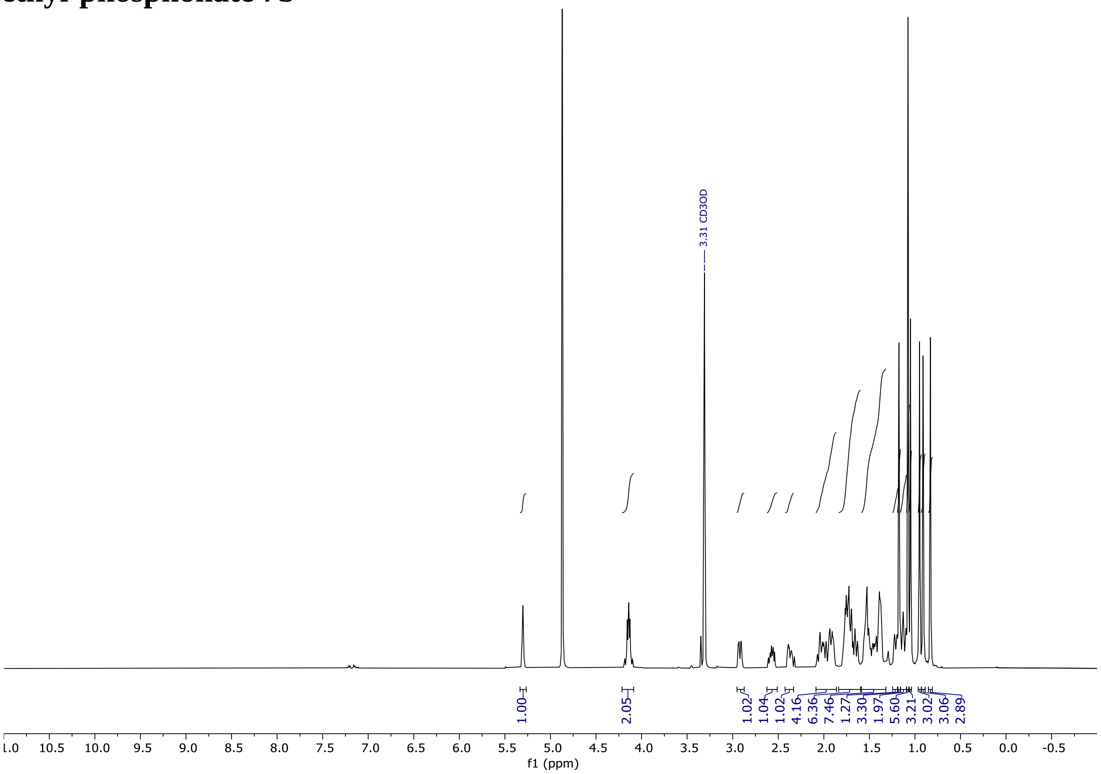

^{13}C NMR (126 MHz, MeOD$_{d4}$) spectrum of sodium (3-oxo-(17R)-17-28-norolean-12(13)-en)-2-oxoethyl-phosphonate 7b

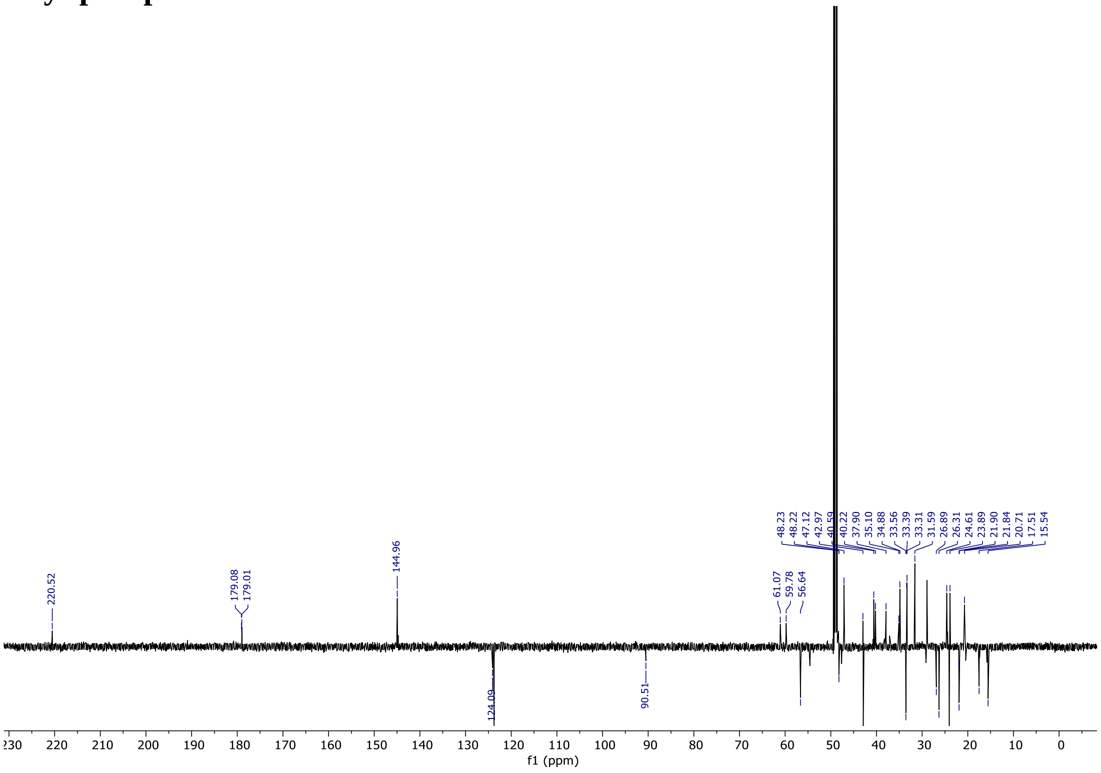

^{1}H NMR (500 MHz, MeOD$_{d4}$) spectrum of sodium (3-oxo-(17R)-17-28-norurs-12(13)-en)-2-oxoethyl-phosphonate 7c

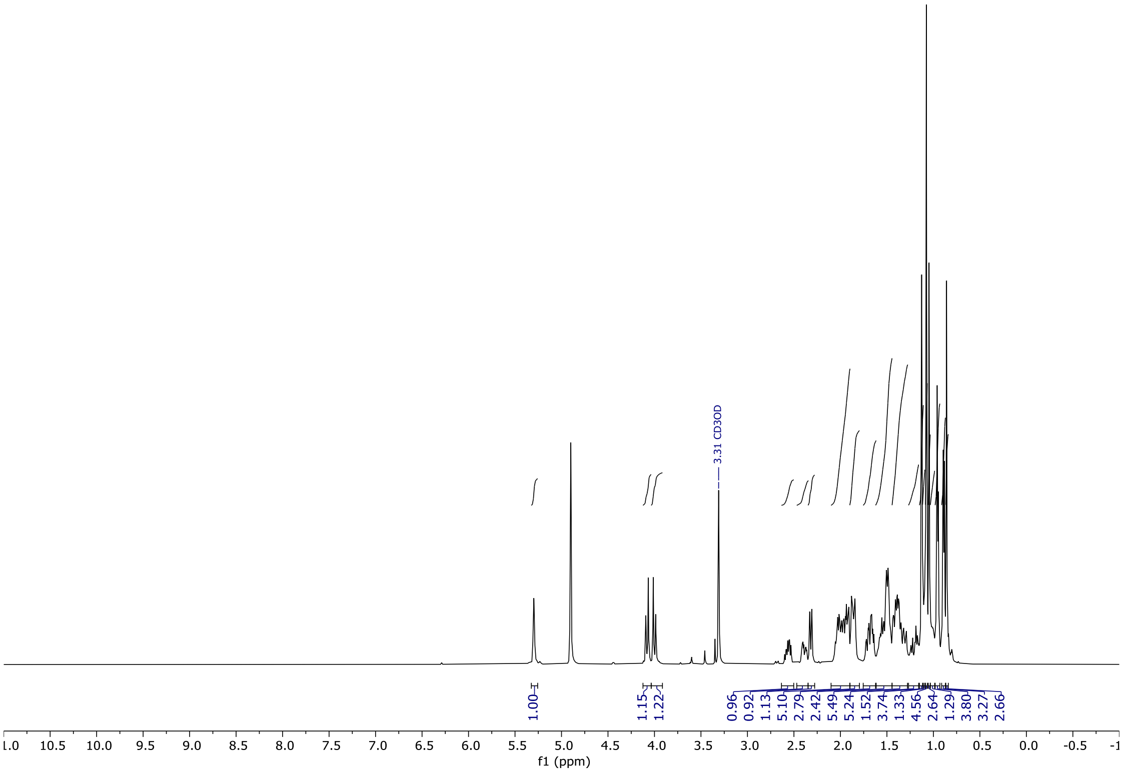

^{13}C NMR (126 MHz, MeOD$_{d4}$) spectrum of sodium (3-oxo-(17R)-17-28-norurs-12(13)-en)-2-oxoethyl-phosphonate 7c

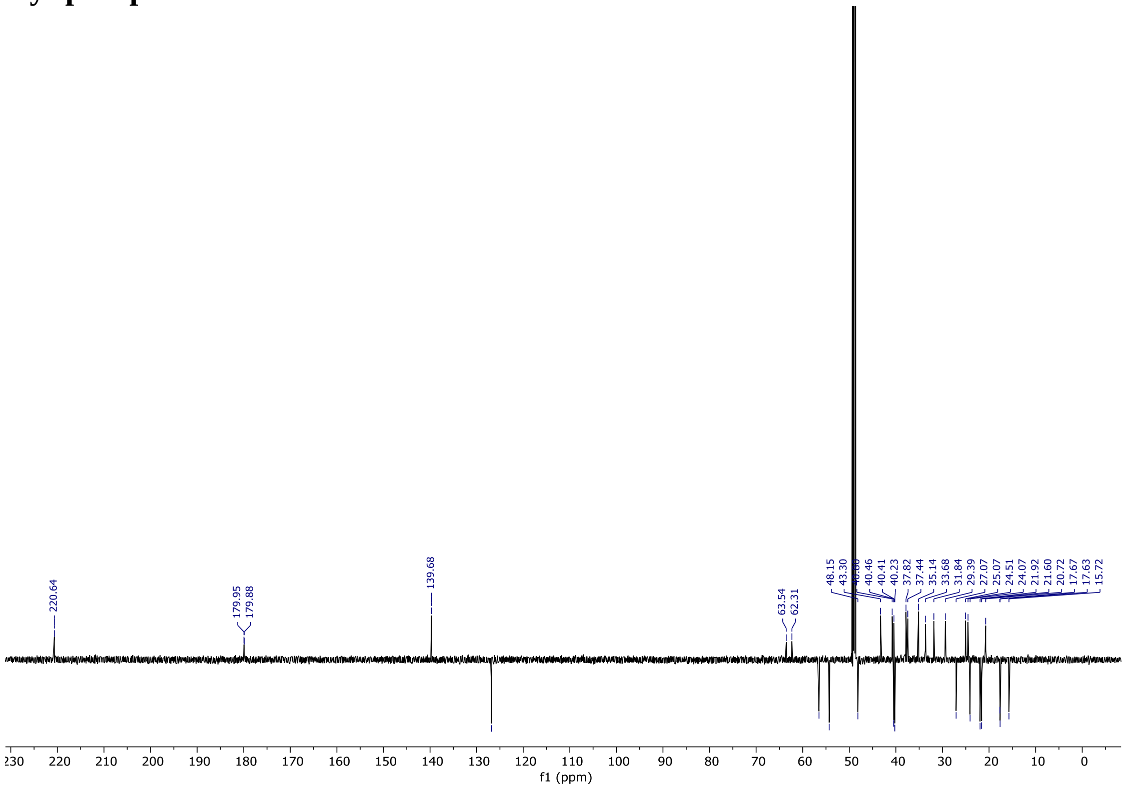

^{1}H NMR (500 MHz, MeOD$_{d4}$) spectrum of sodium (3β-hydroxy-(17R)-17-28-norlup-20(29)-en)-2-oxoethyl-phosphonate 8a

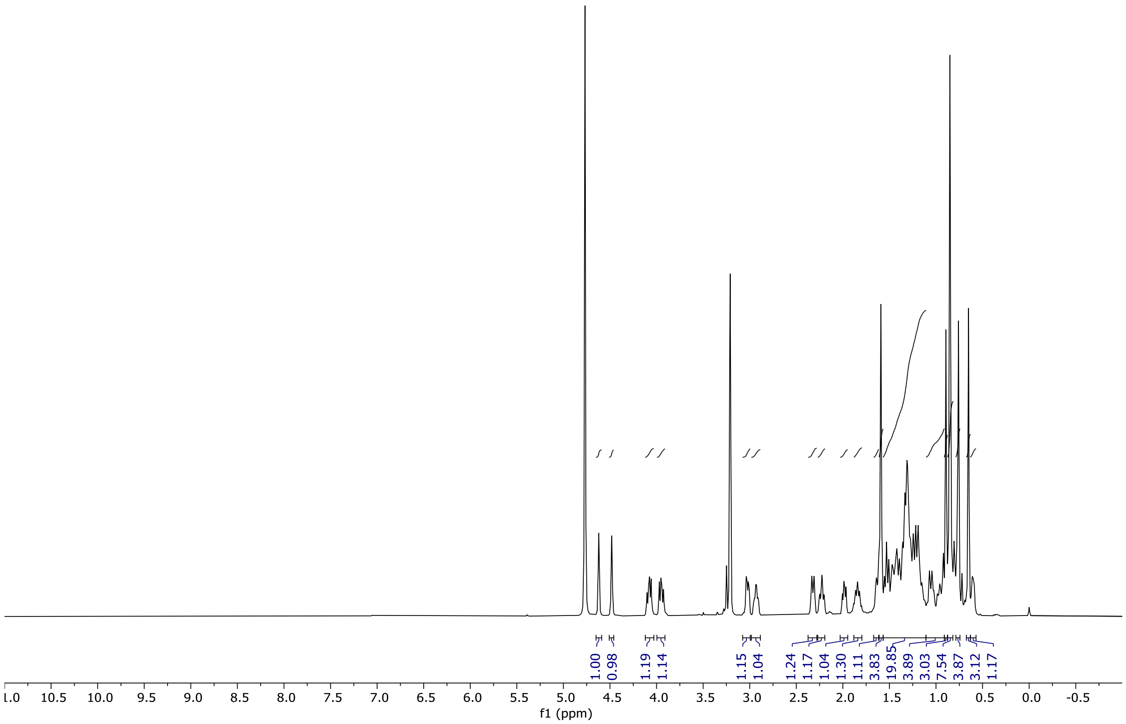

^{13}C NMR (126 MHz, MeOD$_{d4}$) spectrum of sodium (3β-hydroxy-(17R)-17-28-norlup-20(29)-en)-2-oxoethyl-phosphonate 8a

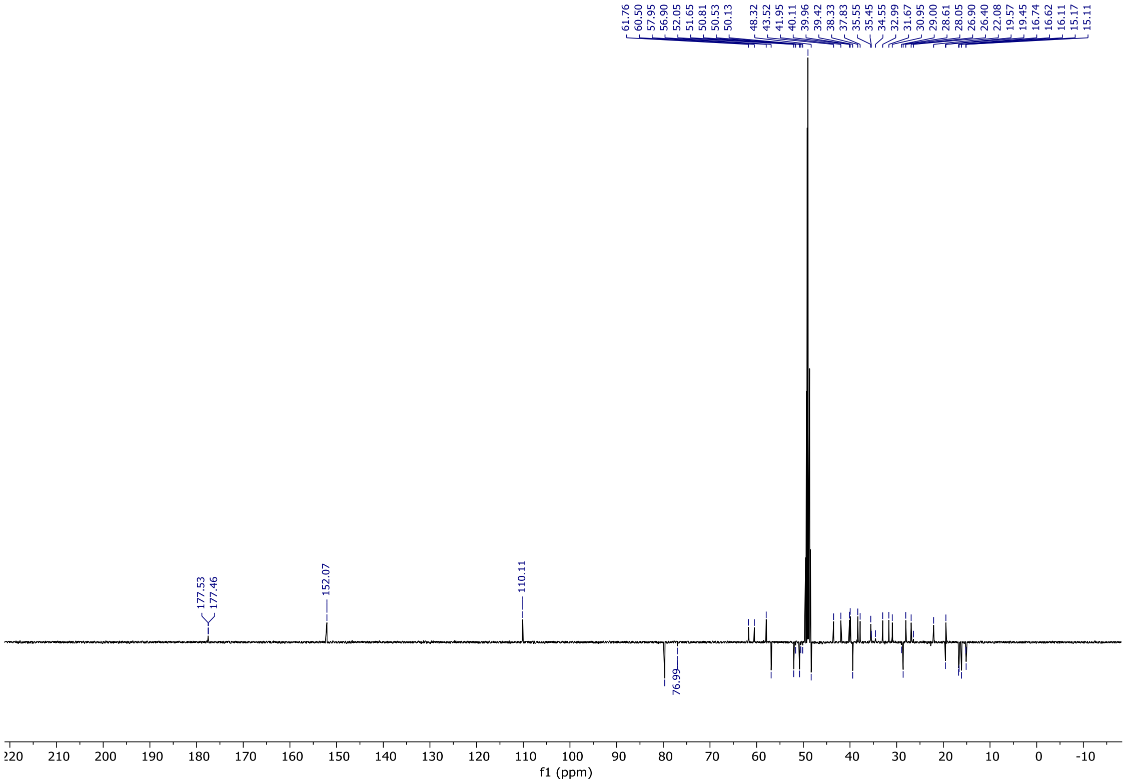

^{1}H NMR (500 MHz, MeOD$_{d4}$) spectrum of sodium (3β-hydroxy-(17R)-17-28-norolean-12(13)-en)-2-oxoethyl-phosphonate 8b

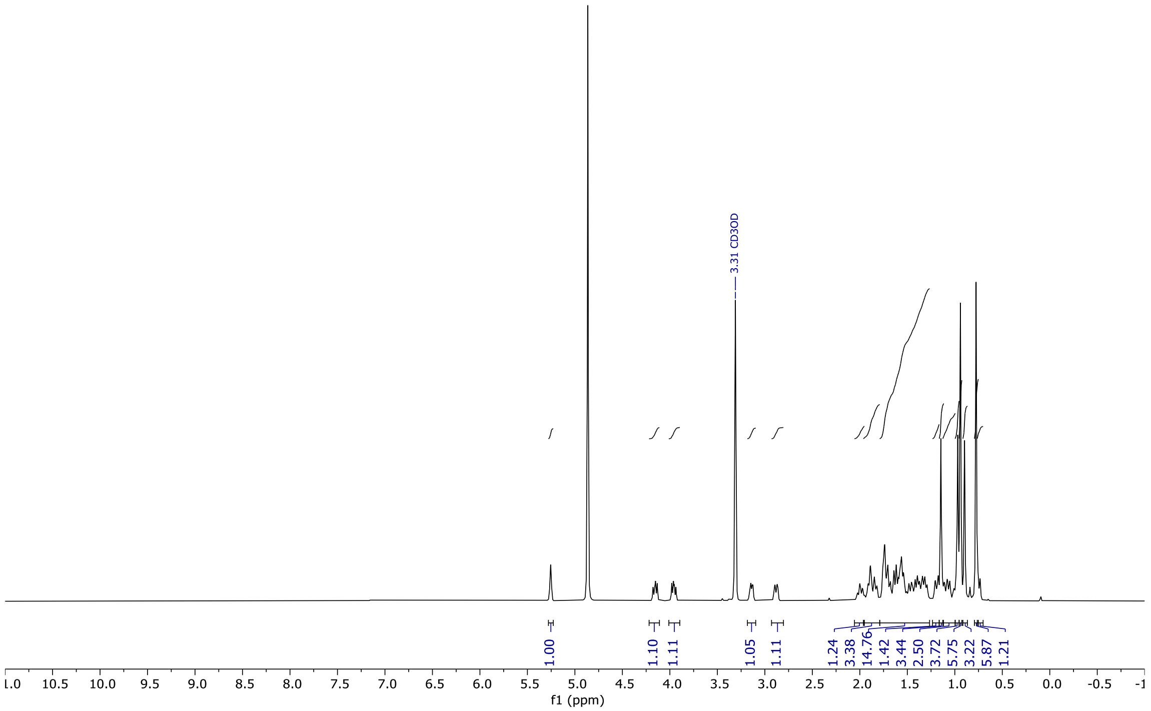

^{13}C NMR (126 MHz, MeOD$_{d4}$) spectrum of sodium (3β-hydroxy-(17R)-17-28-norolean-12(13)-en)-2-oxoethyl-phosphonate 8b

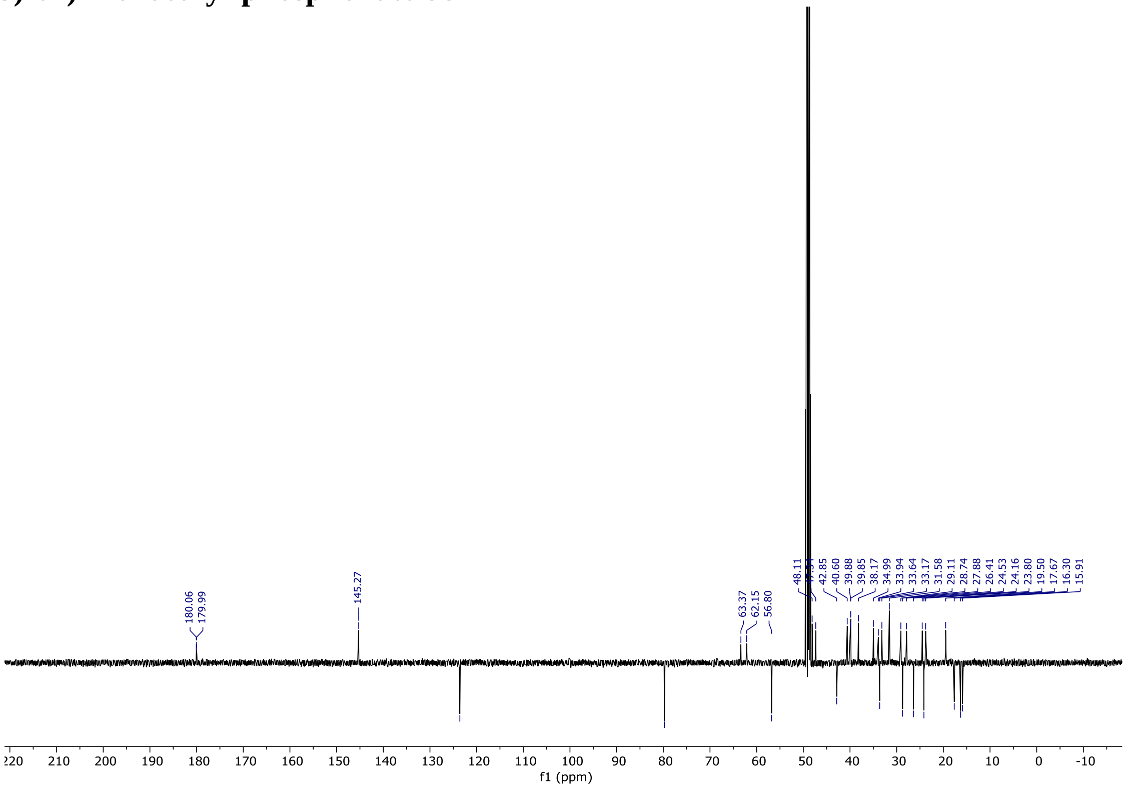

^{13}C NMR (126 MHz, MeOD$_{d4}$) spectrum of sodium (3β-hydroxy-(17R)-17-28-norurs-12(13)-en)-2-oxoethyl-phosphonate 8c

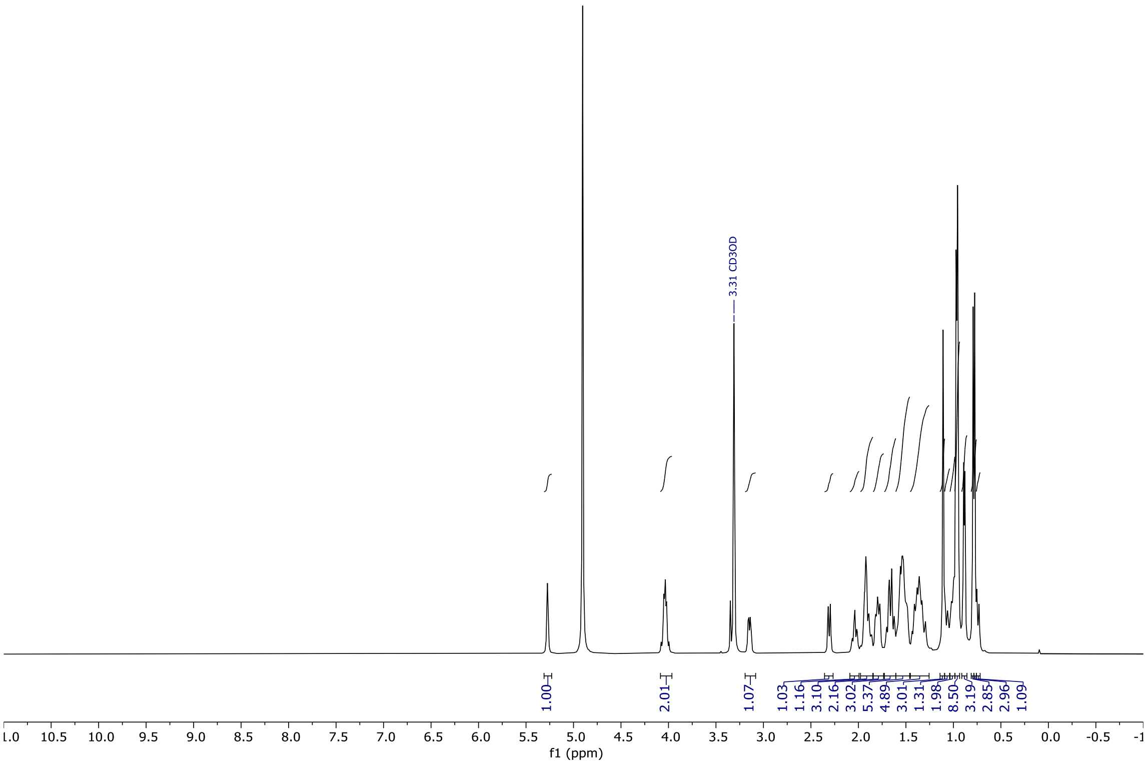

^{13}C NMR (126 MHz, MeOD$_{d4}$) spectrum of sodium (3β-hydroxy-(17R)-17-28-norurs-12(13)-en)-2-oxoethyl-phosphonate 8c

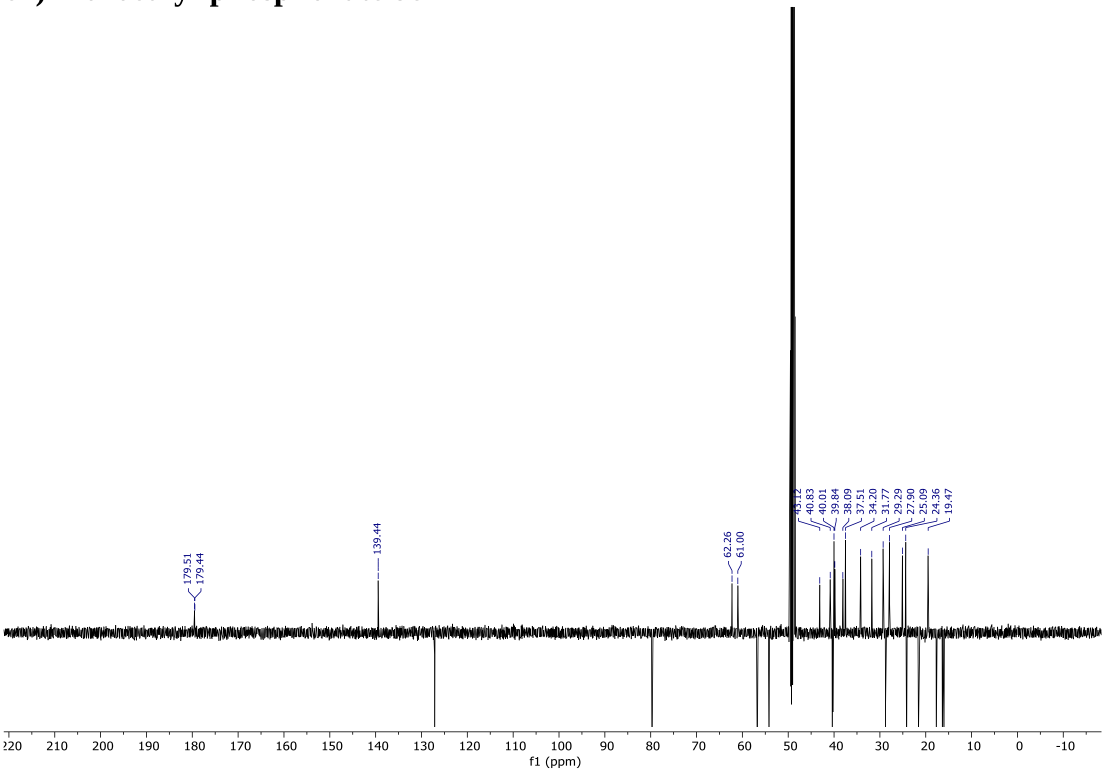

^{1}H NMR (500 MHz, CDCl$_3$) spectrum of 3(S)-3,28-di((dimethoxyphosphoryl)methyloxy)-lup-20(29)ene 9

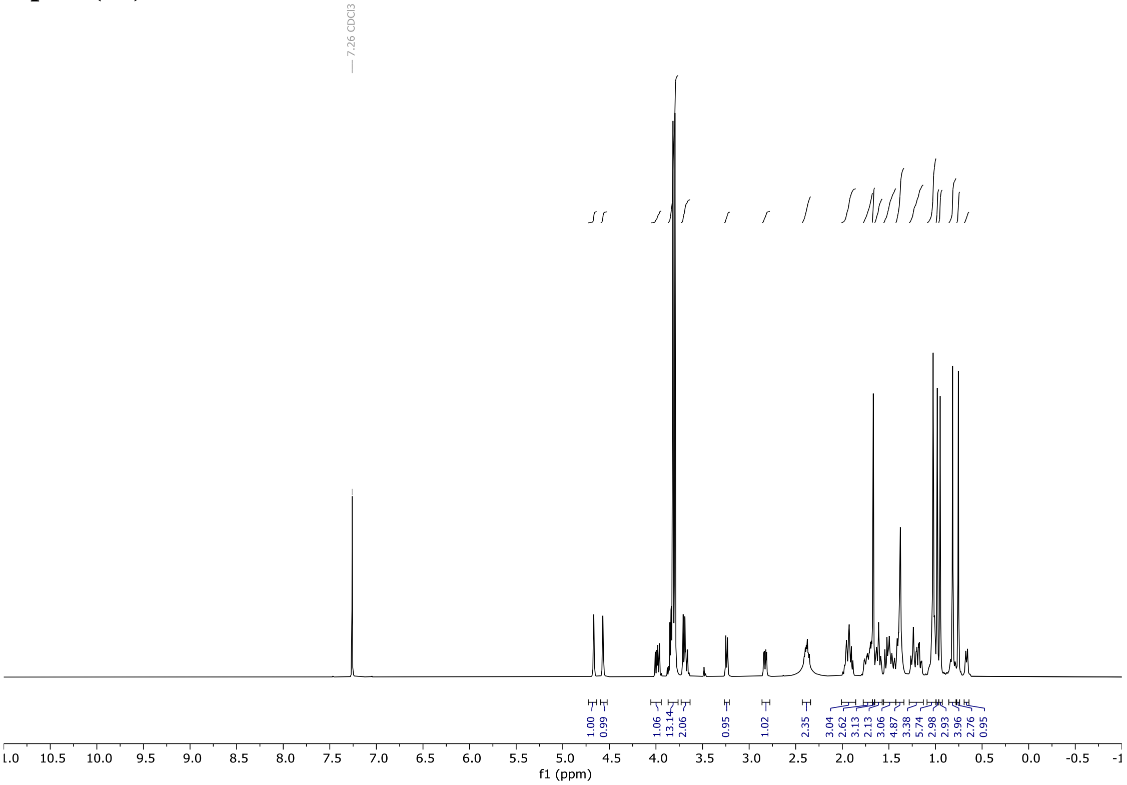

^{13}C NMR (126 MHz, CDCl$_3$) spectrum of 3(S)-3,28-di((dimethoxyphosphoryl)methyloxy)-lup-20(29)ene 9

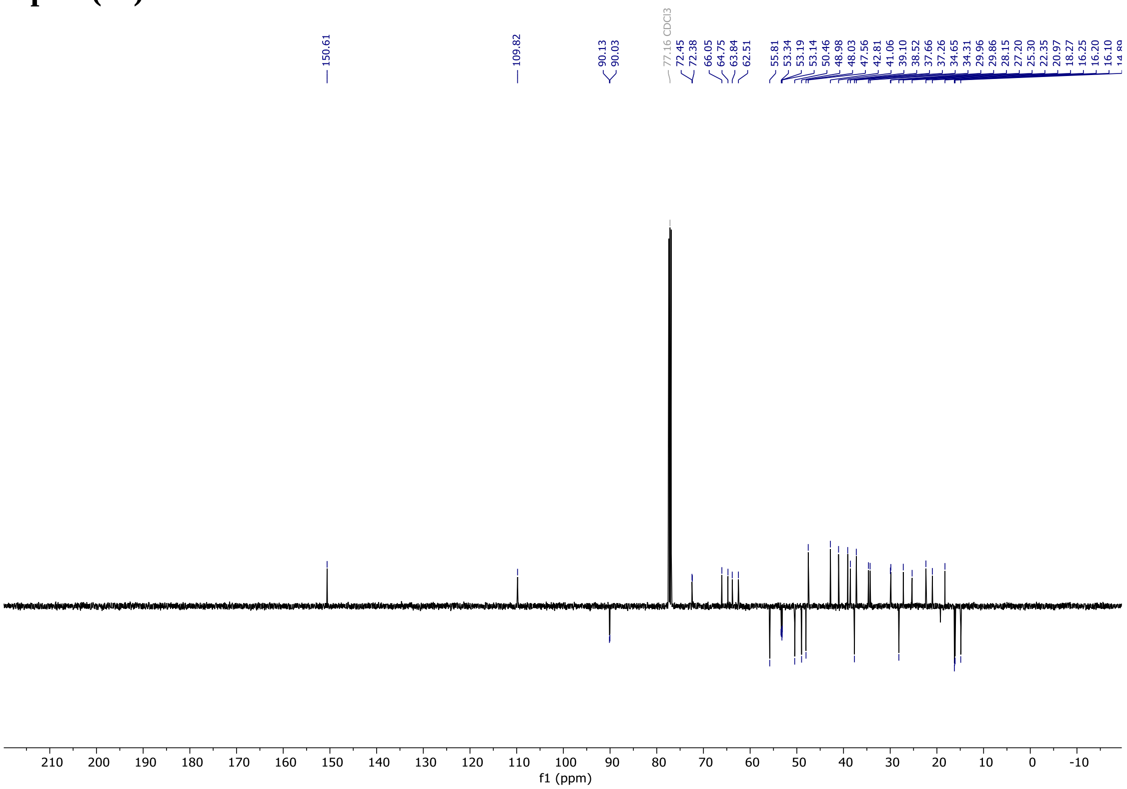

^{1}H NMR (500 MHz, $CDCl_3$) spectrum of ((3(S)-3-((dimethoxyphosphoryl)methyloxy)-28-lup-20(29)enyloxy)(methoxy)phosphoryl)methyl trifluoromethanesulfonate 11

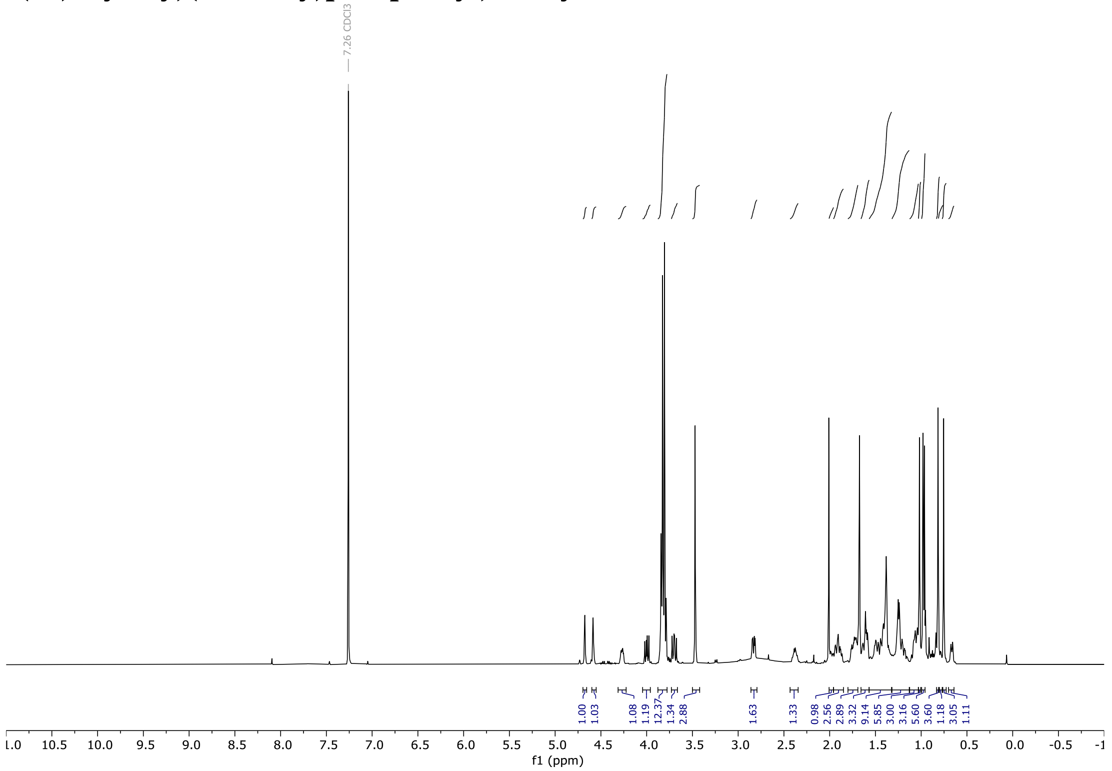

^{13}C NMR (126 MHz, $CDCl_3$) spectrum of ((3(S)-3-((dimethoxyphosphoryl)methyloxy)-28-lup-20(29)enyloxy)(methoxy)phosphoryl)methyl trifluoromethanesulfonate 11

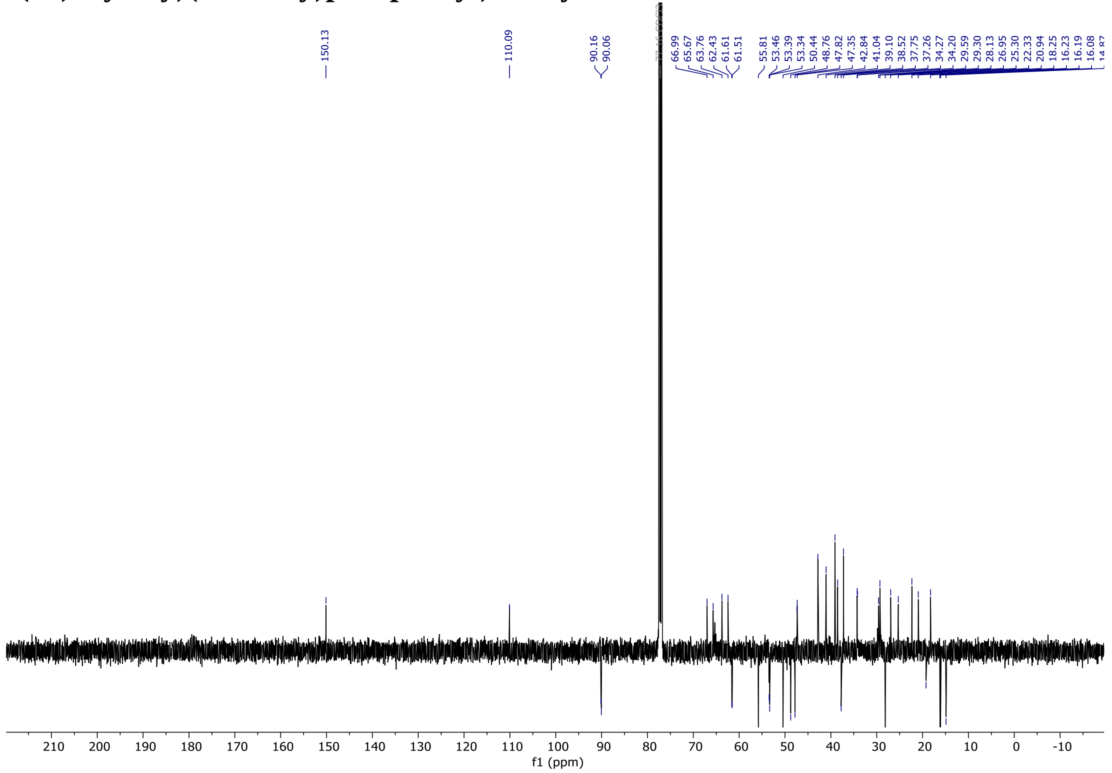

^{1}H NMR (500 MHz, MeOD$_{d4}$) spectrum of sodium (lup-20(29)-en-3(S)-3,28 diylbis(oxymethylene))bis(phosphonate) 12

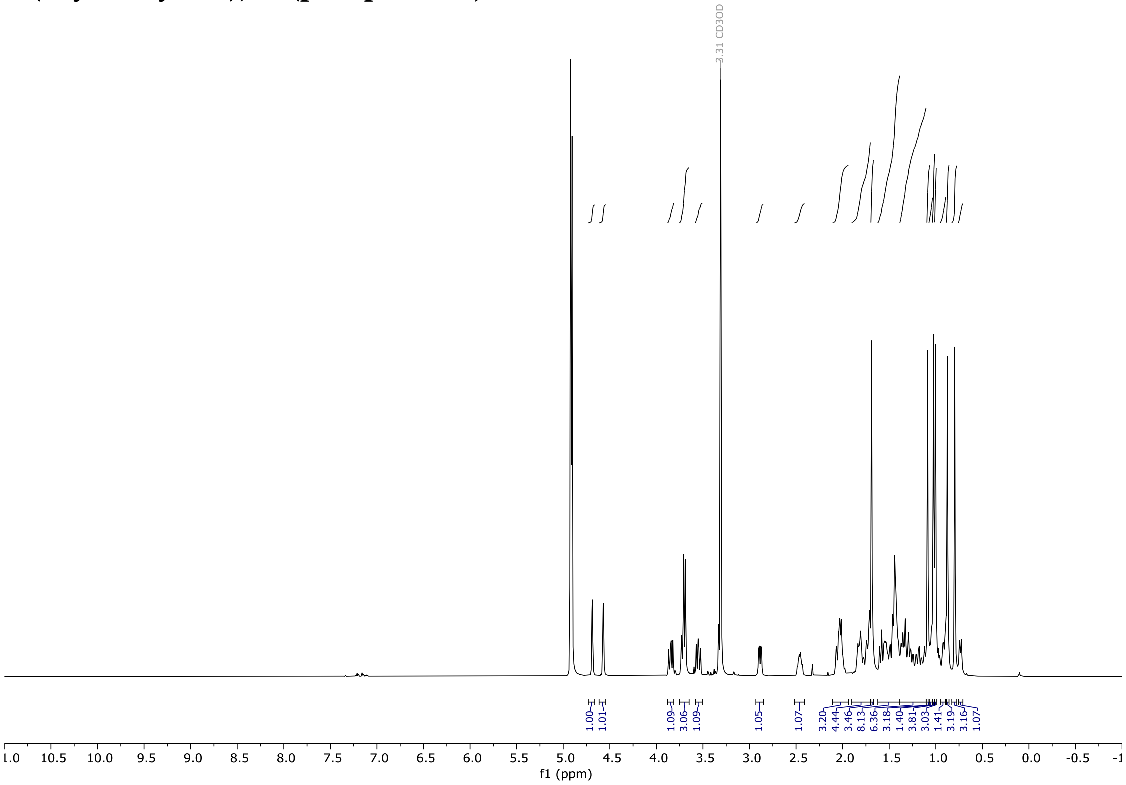

^{13}C NMR (126 MHz, MeOD$_{d4}$) spectrum of sodium (lup-20(29)-en-3(S)-3,28 diylbis(oxymethylene))bis(phosphonate) 12

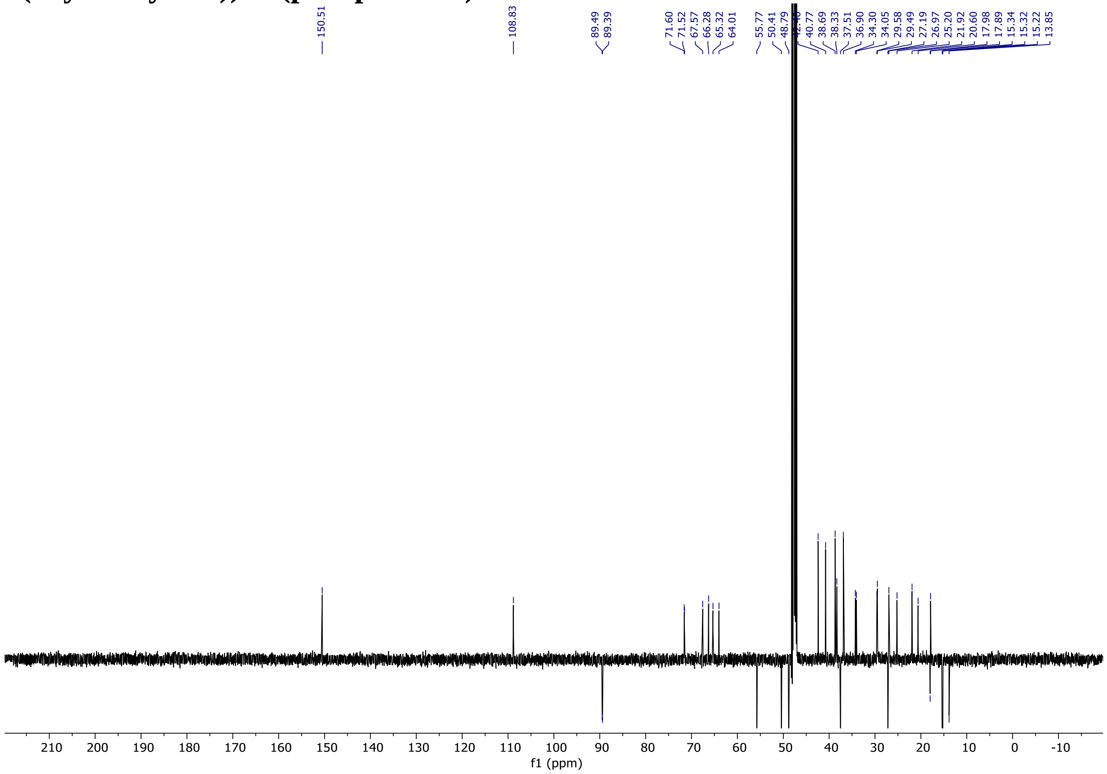

^{31}P NMR (121 MHz, $CDCl_3$) spectrum of 3-oxo-(17S)-17-(((dimethoxyphosphoryl)methoxy)carbonyl)-28-norlup-20(29)ene 3a

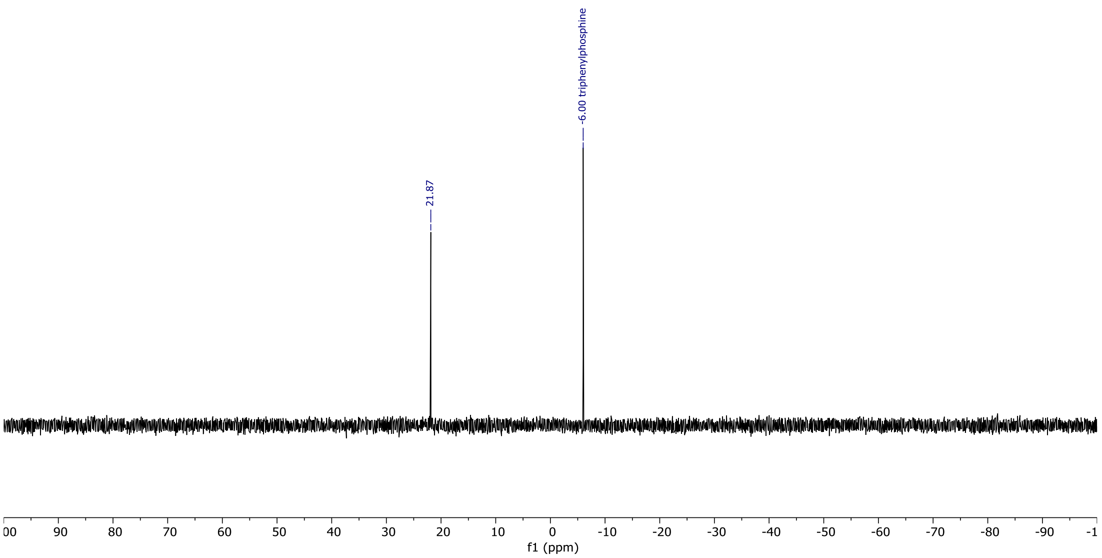

^{31}P NMR (121 MHz, $CDCl_3$) spectrum of 3-oxo-(17S)-17-(((dimethoxyphosphoryl)methoxy)carbonyl)-28-norolean-12(13)-ene 3b

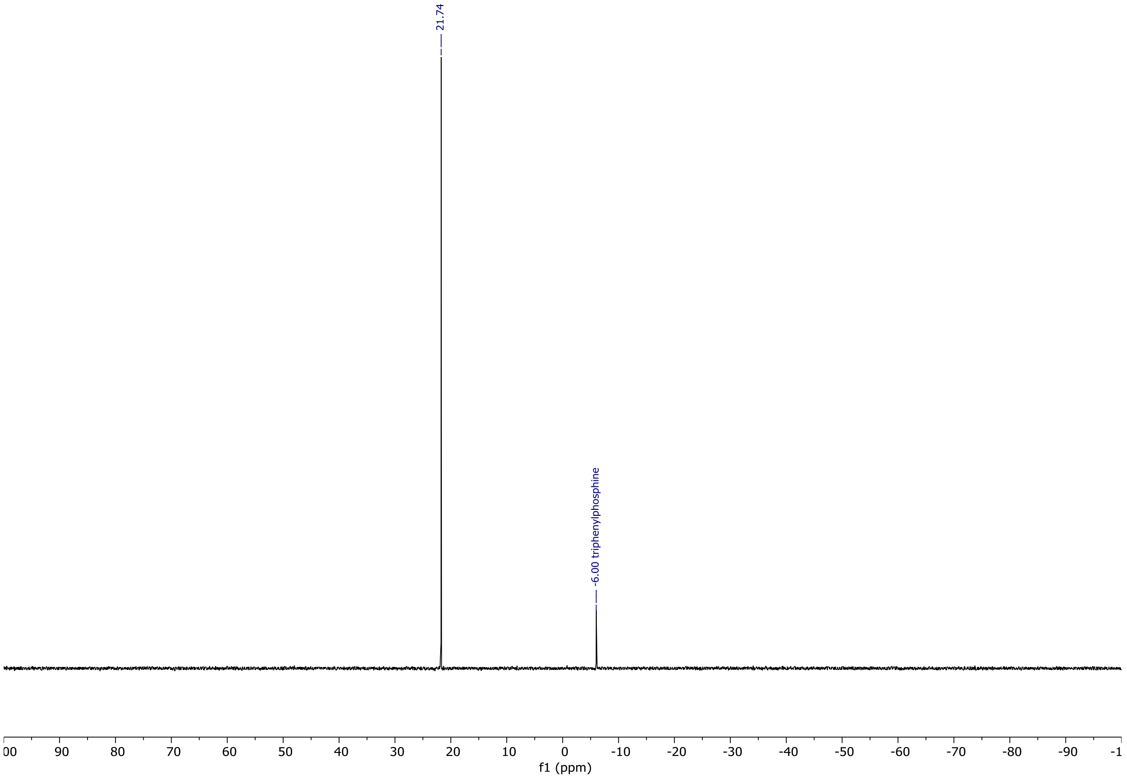

^{31}P NMR (121 MHz, $CDCl_3$) spectrum of 3-oxo-(17S)-17-(((dimethoxyphosphoryl)methoxy)carbonyl)-28-norurs-12(13)-ene 3c

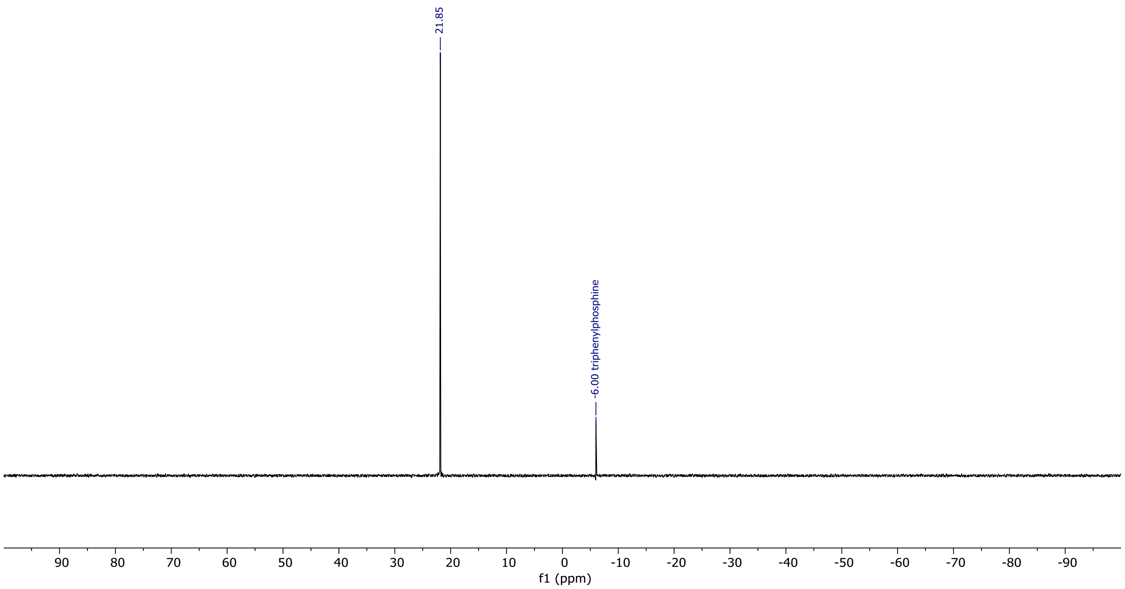

^{31}P NMR (121 MHz, $CDCl_3$) spectrum of (17S)-17-(((dimethoxyphosphoryl)methoxy)carbonyl)-3β-hydroxy-28-norlup-20(29)ene 4a

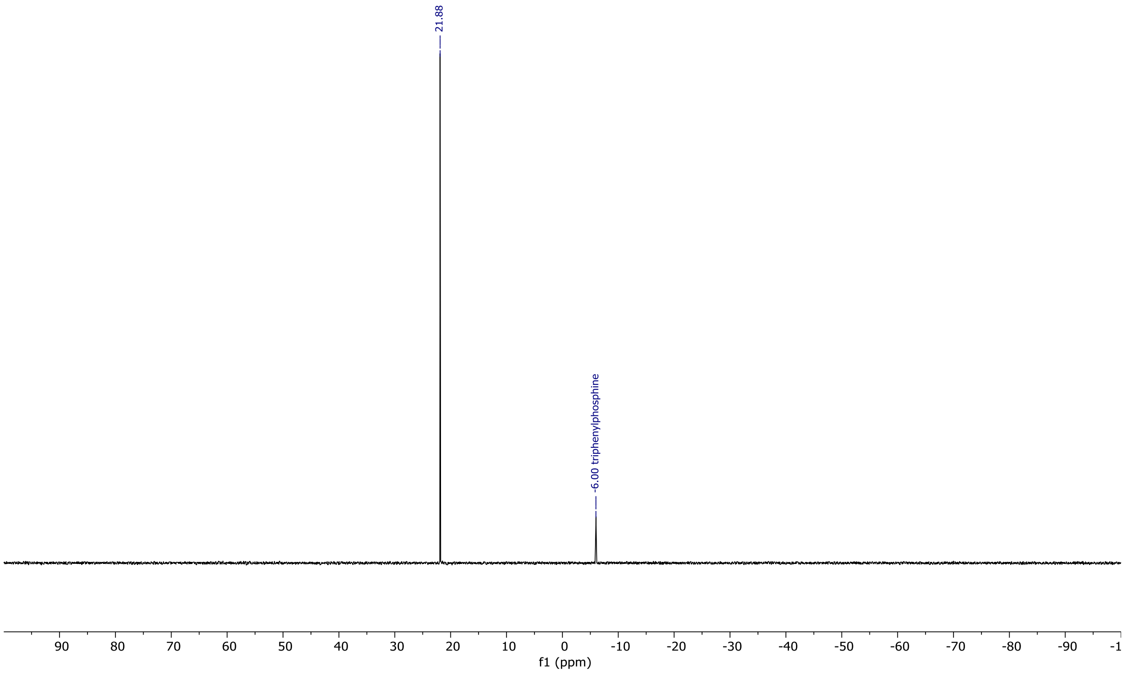

^{31}P NMR (121 MHz, CDCl$_3$) spectrum of (17S)-17-(((dimethoxyphosphoryl)methoxy)carbonyl)-3β-hydroxy -28-norolean-12(13)-ene 4b

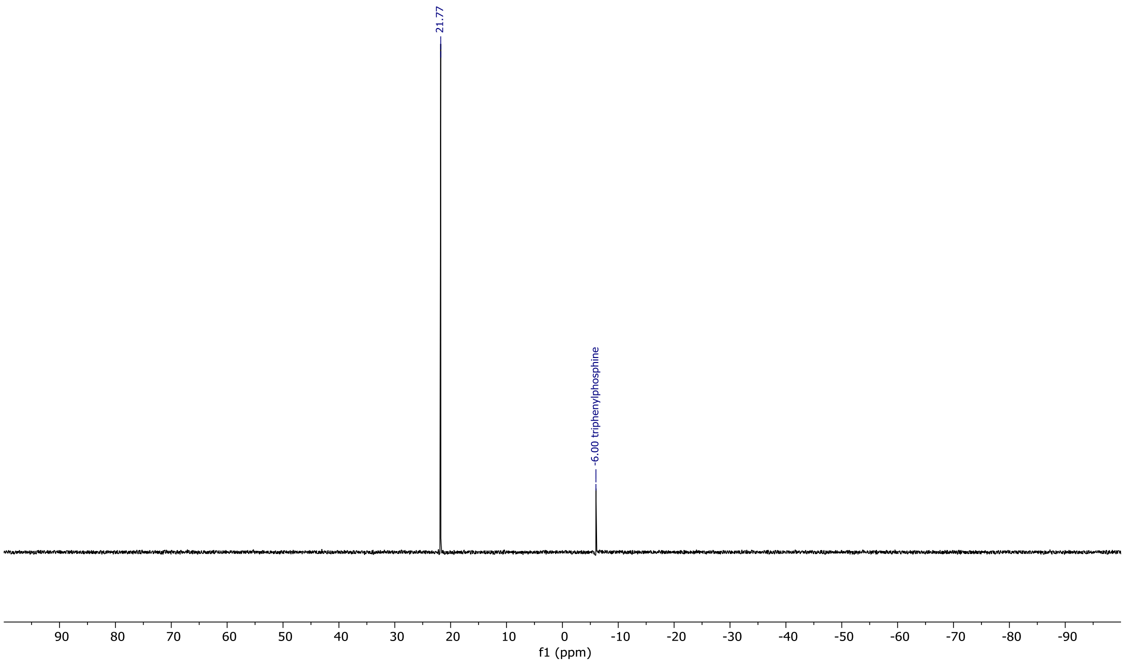

^{31}P NMR (121 MHz, CDCl$_3$) spectrum of (17S)-17-(((dimethoxyphosphoryl)methoxy)carbonyl)-3β-hydroxy -28-norurs-12(13)-ene 4c

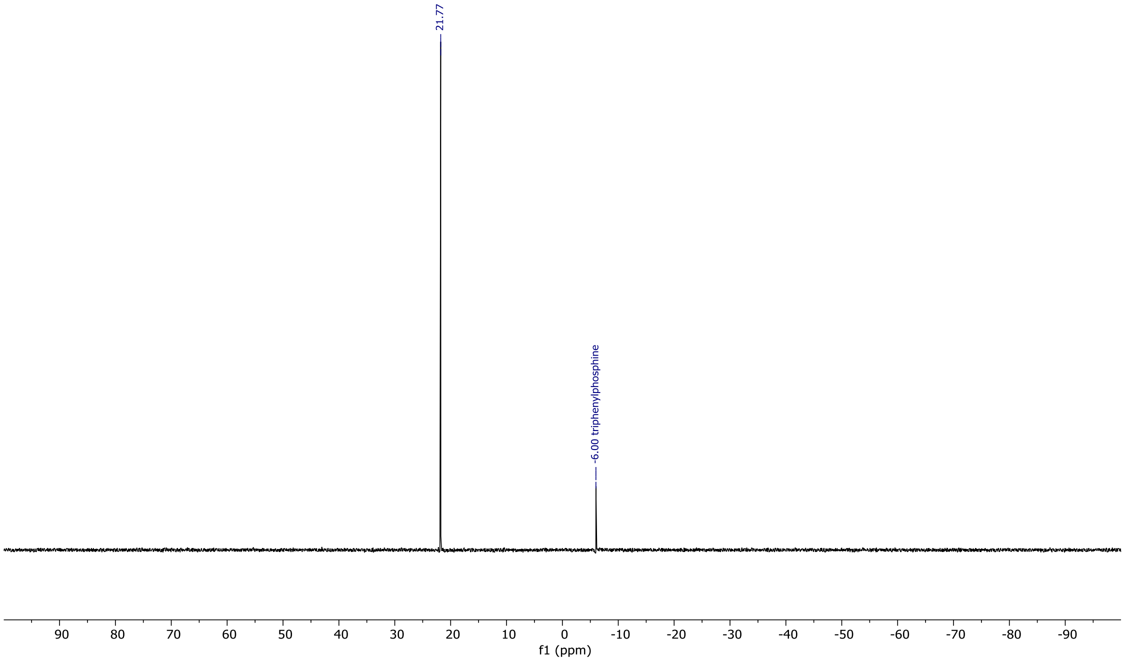

^{31}P NMR (121 MHz, MeOD$_{d4}$) spectrum of sodium (3-oxo-(17R)-17-28-norlup-20(29)-en)-2-oxoethyl-phosphonate 7a

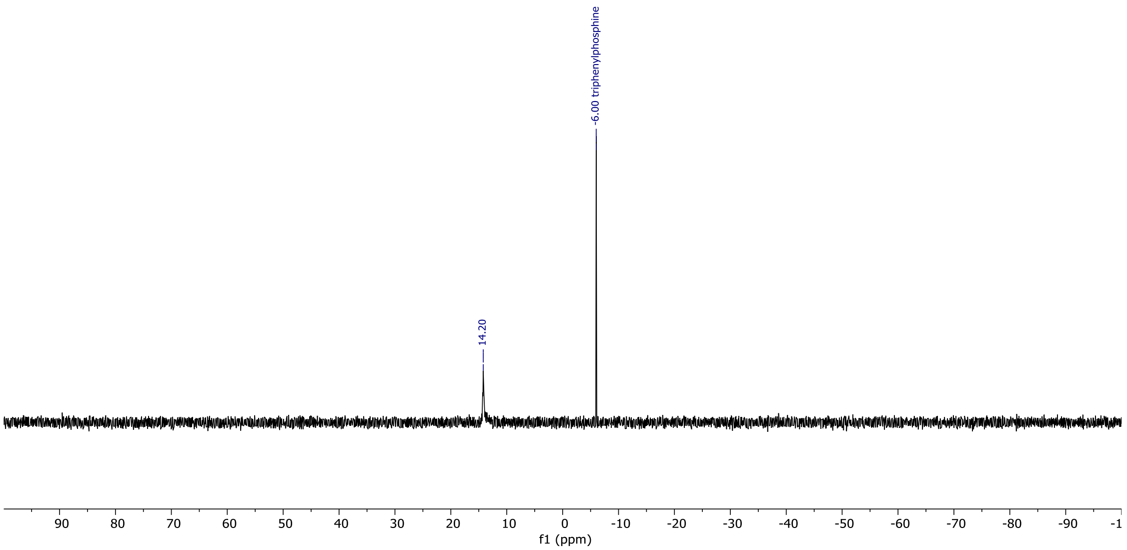

^{31}P NMR (121 MHz, MeOD$_{d4}$) spectrum of sodium (3-oxo-(17R)-17-28-norolean-12(13)-en)-2-oxoethyl-phosphonate 7b

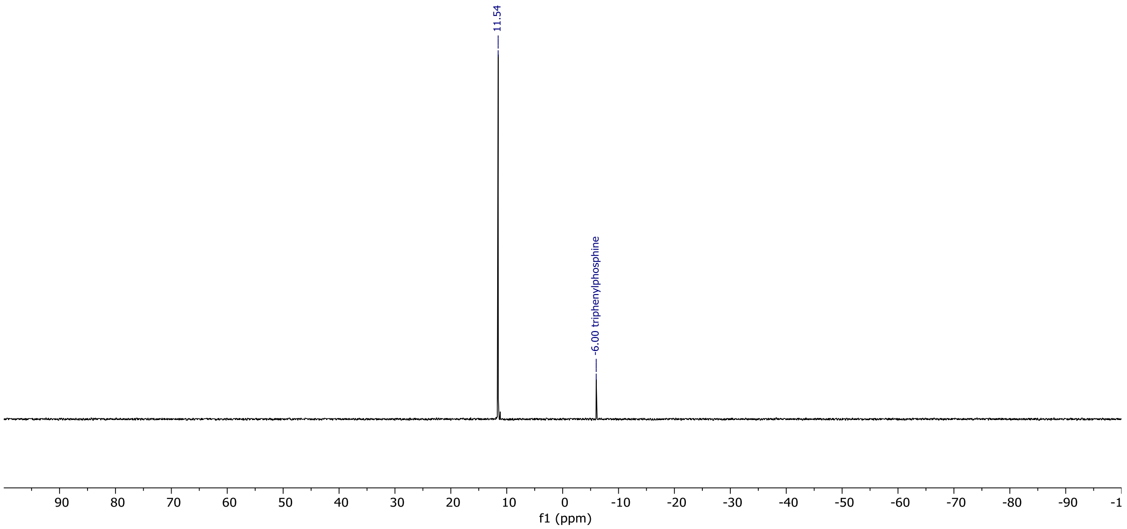

^{31}P NMR (121 MHz, MeOD*$_{d4}$*) spectrum of sodium (3-oxo-(17R)-17-28-norurs-12(13)-en)-2-oxoethyl-phosphonate 7c

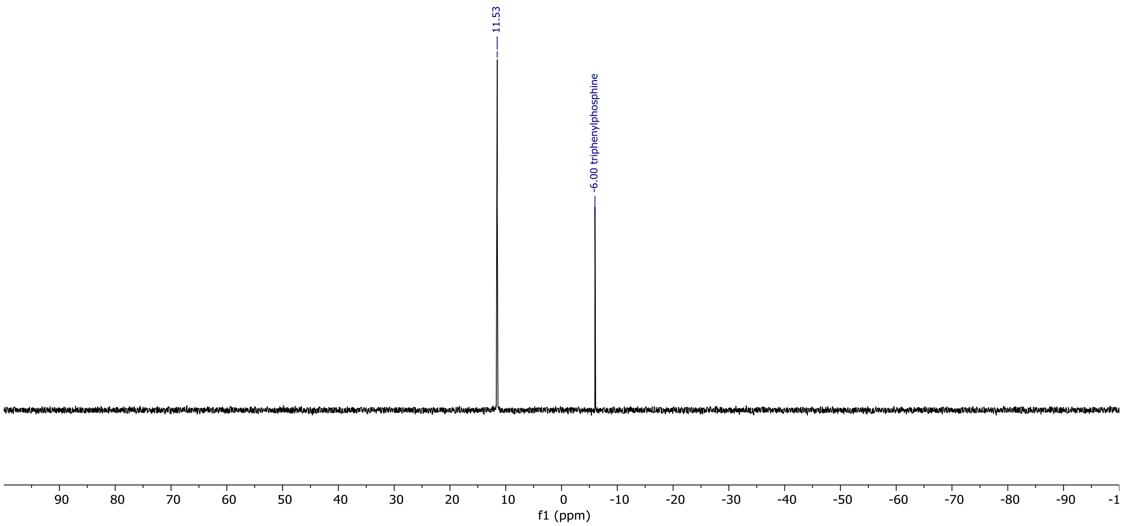

^{31}P NMR (121 MHz, MeOD*$_{d4}$*) spectrum of sodium (3β-hydroxy-(17R)-17-28-norlup-20(29)-en)-2-oxoethyl-phosphonate 8a

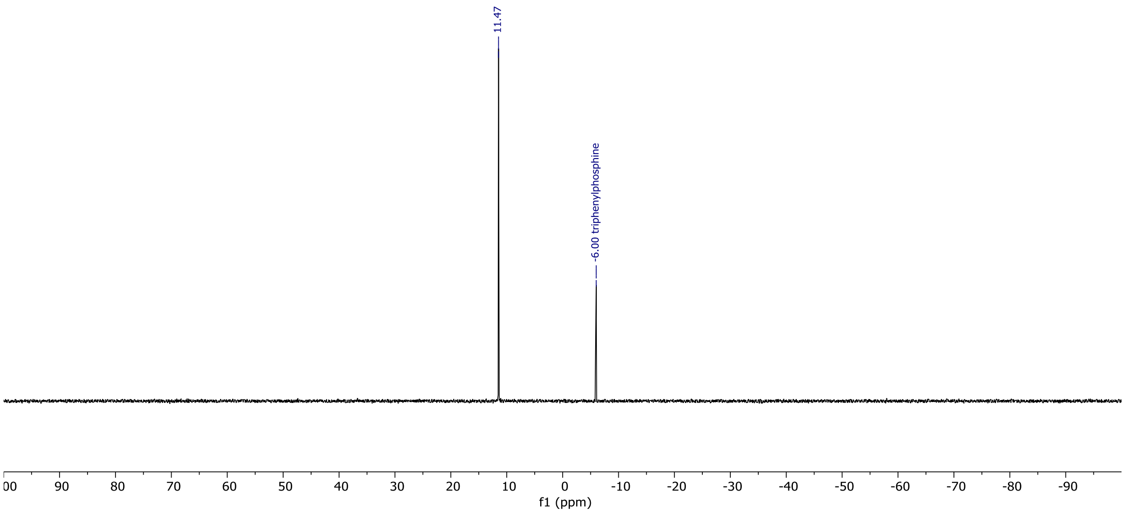

^{31}**P NMR (121 MHz, MeOD$_{d4}$) spectrum of sodium (3β-hydroxy-(17R)-17-28-norolean-12(13)-en)-2-oxoethyl-phosphonate 8b**

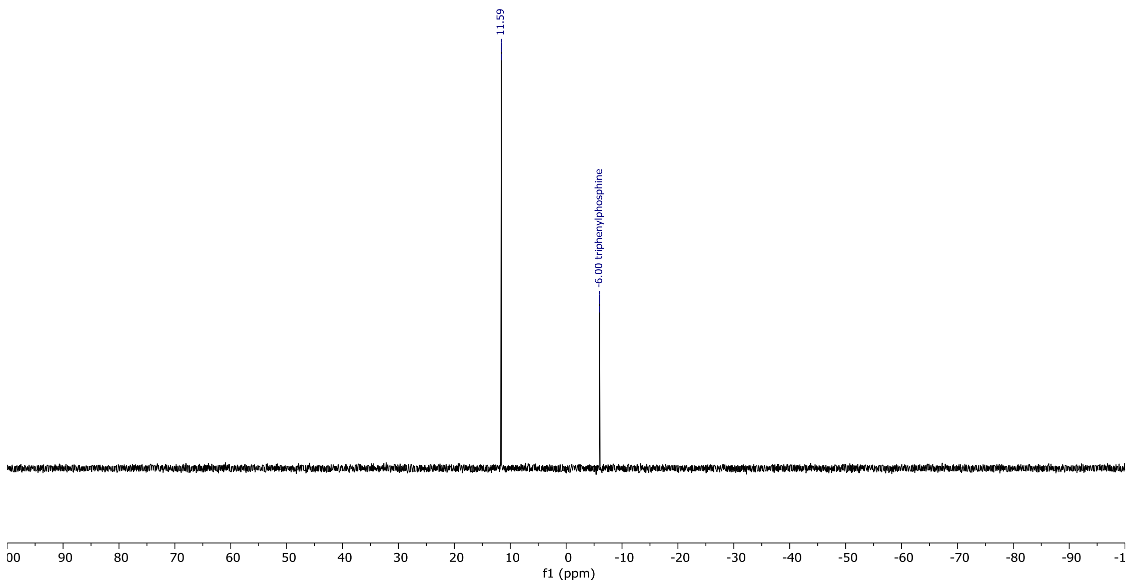

^{31}**P NMR (121 MHz, MeOD$_{d4}$) spectrum of sodium (3β-hydroxy-(17R)-17-28-norurs-12(13)-en)-2-oxoethyl-phosphonate 8c**

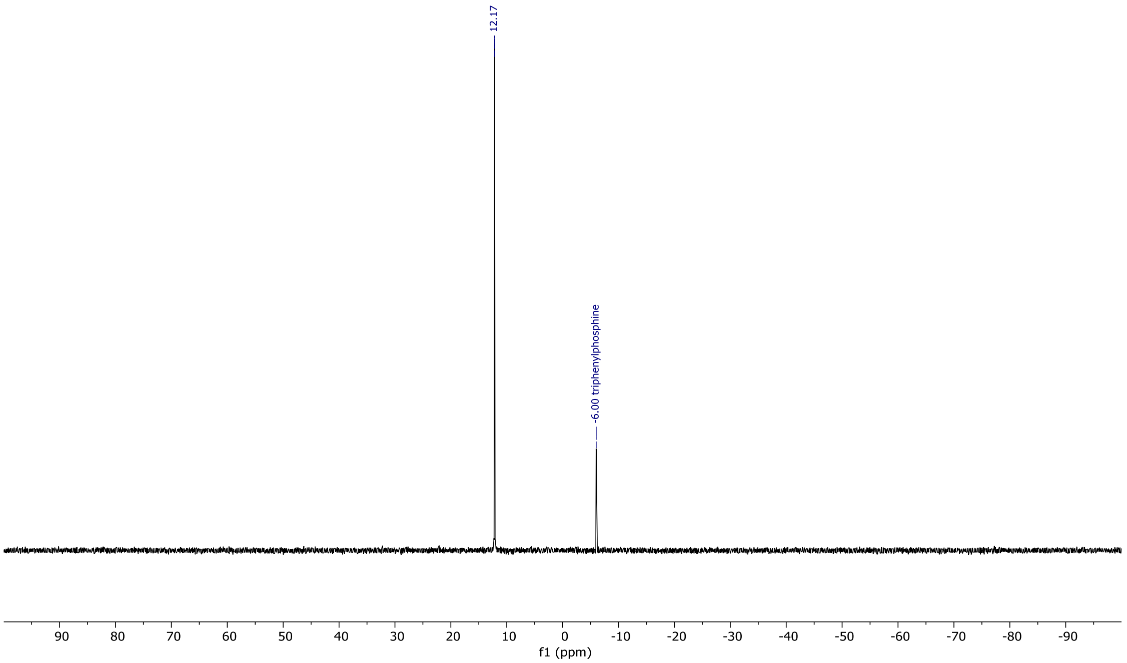

^{31}P NMR (121 MHz, $CDCl_3$) spectrum of 3(S)-3,28-di((dimethoxyphosphoryl)methyloxy)-lup-20(29)ene 9

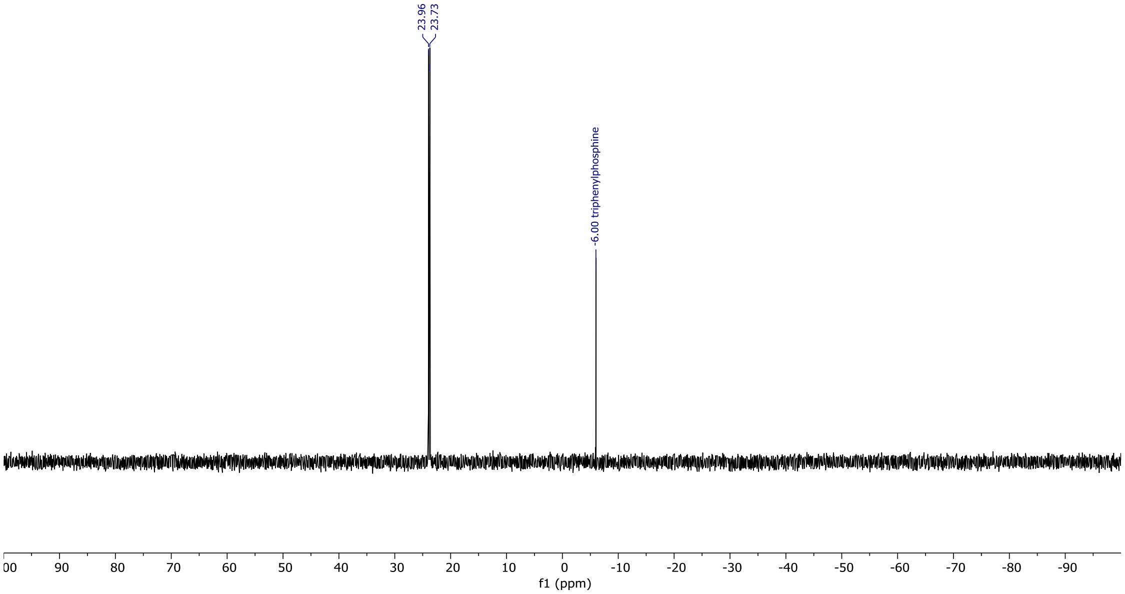

^{31}P NMR (121 MHz, $CDCl_3$) spectrum of ((3(S)-3-((dimethoxyphosphoryl)methyloxy)-28-lup-20(29)enyloxy)(methoxy)phosphoryl)methyl trifluoromethanesulfonate 11

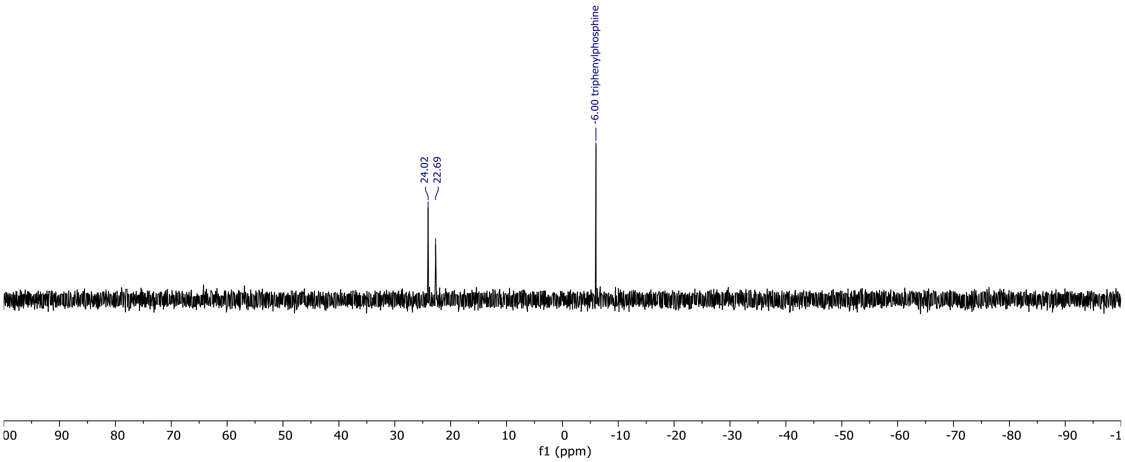

^{31}P NMR (121 MHz, MeOD$_{d4}$) spectrum of sodium (lup-20(29)-en-3(S)-3,28-diylbis(oxymethylene))bis(phosphonate) 12

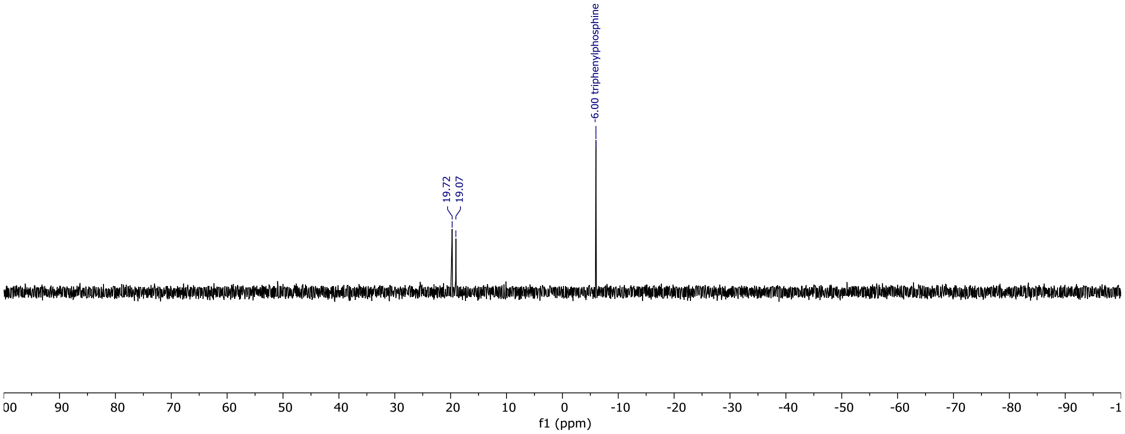

Water solubility determination

To suspension of PCT phosphonate sample (10 mg) in D_2O (500 µL) 0.8% solution of NaOD in D_2O was added dropwise until pH = 8-8.5 (<100 µL). Obtained suspension was sonified in ultrasonic bath for 10 min. Undissolved particles were separated by centrifugation. Clear D_2O solution was carefully transferred to NMR tube equipped with external standard vial.

Formula (1) was used to determine the solubility of PCT sample in the D_2O.

$$Sol_{PCT} = \frac{I_{PCT}}{I_{st}} \times \frac{N_{st}}{N_{PCT}} \times \frac{m_{st}}{M_{st}} \times \frac{M_{PCT}}{V} \times W_{st} \qquad (1)$$

where:

Sol_{PCT} - solubility of PCT sample in D_2O at room temperature (mg/mL)

I_{PCT} - integral value of PCT signal of choice

I_{st} - integral value of external standard signal of choice

N_{PCT} - number of integrated protons of PCT

N_{st} - number of integrated protons of external standard

V - volume of used D_2O (mL)

m_{st} - mass of external standard (mg)

M_{bet} - molecular weight of PCT (g•mol^{-1})

M_{st} - molecular weight of external standard - potassium hydrogen phthalate (204.22 g•mol^{-1})

W_{st} - assay of the external standard (99.5 %)

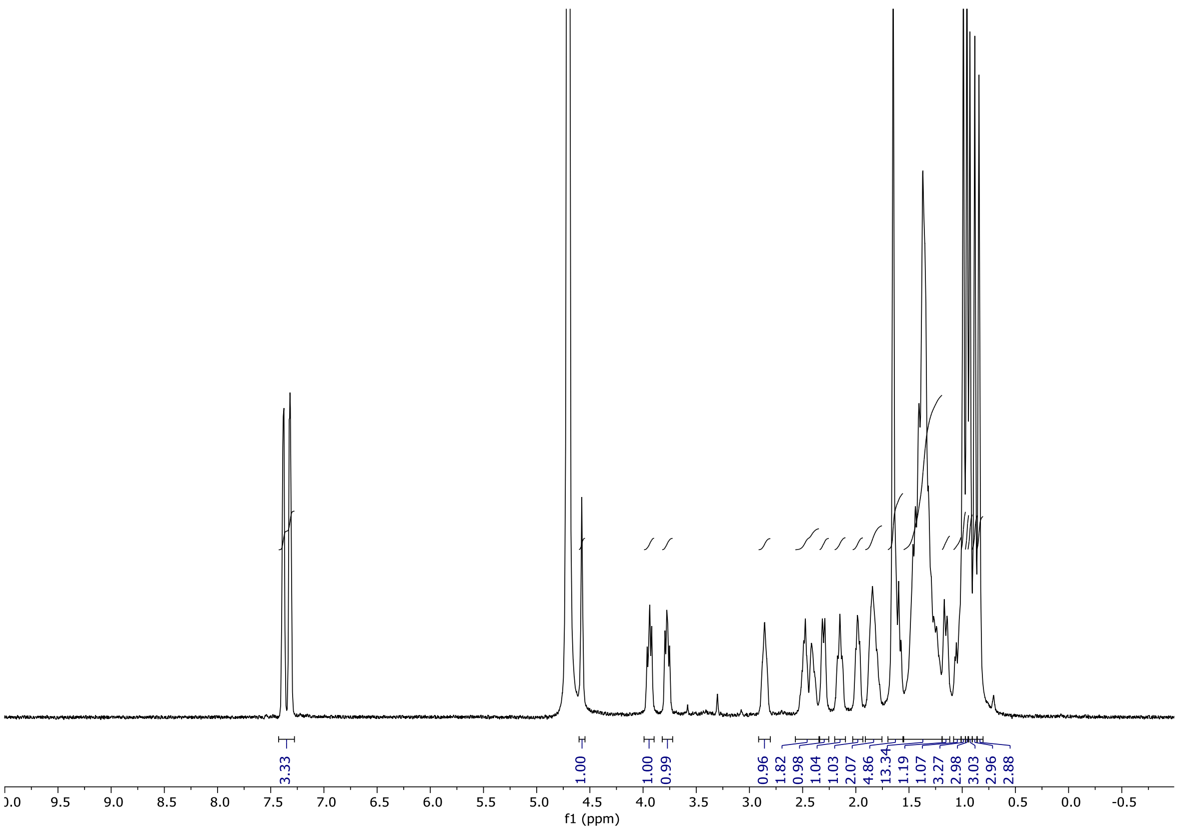

^{1}H NMR (500 MHz, D_2O)) spectrum of sodium (3-oxo-(17R)-17-28-norlup-20(29)-en)-2-oxoethyl-phosphonate 7a and potassium hydrogen phthalate

$$Sol_{PCT} = \frac{1}{3.33} \times \frac{4}{1} \times \frac{1.9}{204.22} \times \frac{592.66}{0.595} \times 0.995 = 11.07\ mg/mL$$

where:

I_{PCT} - integral value of PCT signal of choice (1)

I_{st} - integral value of external standard signal of choice (3.33)

N_{PCT} - number of integrated protons of PCT (1)

N_{st} - number of integrated protons of external standard (4)

V - volume of used D_2O (500 µL + 95 µL from NaOD solution = 595 µL)

m_{st} - mass of external standard (119 µL from 32 mg/2mL solution, 119/2000 ·32 = 1.9 mg)

M_{bet} - molecular weight of PCT (MW of **7a** = 592.66 g•mol^{-1})

M_{st} - molecular weight of external standard - potassium hydrogen phthalate (204.22 g•mol^{-1})

W_{st} - assay of the external standard (99.5%)

CYTOTOXICITY EVALUATION

Cytotoxicity of betulinic acid, ursolic acid and oleanolic acid derivatives was evaluated using human-derived osteosarcoma cell line MG63 (ATCC, CRL-1427) and mouse-derived preosteoblast cell line MC3T3-E1 (ATCC CRL-2593). Before conducting the experiments, both cell lines were continuously cultured according to the ATCC product sheet instructions. Briefly, both cell lines were expanded in α-MEM medium supplemented with 10 % FBS and 1 % pen-strep and maintained at 37 °C in a humidified atmosphere with 5 % CO_2.

Each cell line was plated at a concentration of $1x10^4$ cells/well in 96-well plate. The plates were incubated for 24 hours to allow cell attachment and growth. Bioactive substances were solubilized in methanol prior further dilution in cell culture media. Following the cell incubation period, the culture media was removed and the dilutions of active substance (10, 25 and 50 μM) in cell culture media were added. The culture medium only and its dilutions with methanol solution respective to the bioactive substance concentrations were used as the controls. The final concentration of methanol did not exceed 1%. Cells were treated with bioactive substance solutions for 24 h. After 24 h incubation, relative cell metabolic activity was assessed using CellTiter-Blue® (CTB) analysis (Promega, JAV). Absorbance was measured at 590 nm using microplate reader (Infinite® 200 PRO, Tecan, USA). The relative cell metabolic activity was calculated for each bioactive substance concentration as well as for the controls. All results were presented as the mean ± standard deviation of at least 5 replicates. Statistically significant differences between sample groups are assessed using a one-way ANOVA test and then corrected using the Šídák multiple comparison test. Statistically significant differences were considered to be those with a P-values less than 0.05 ($p < 0.05$).

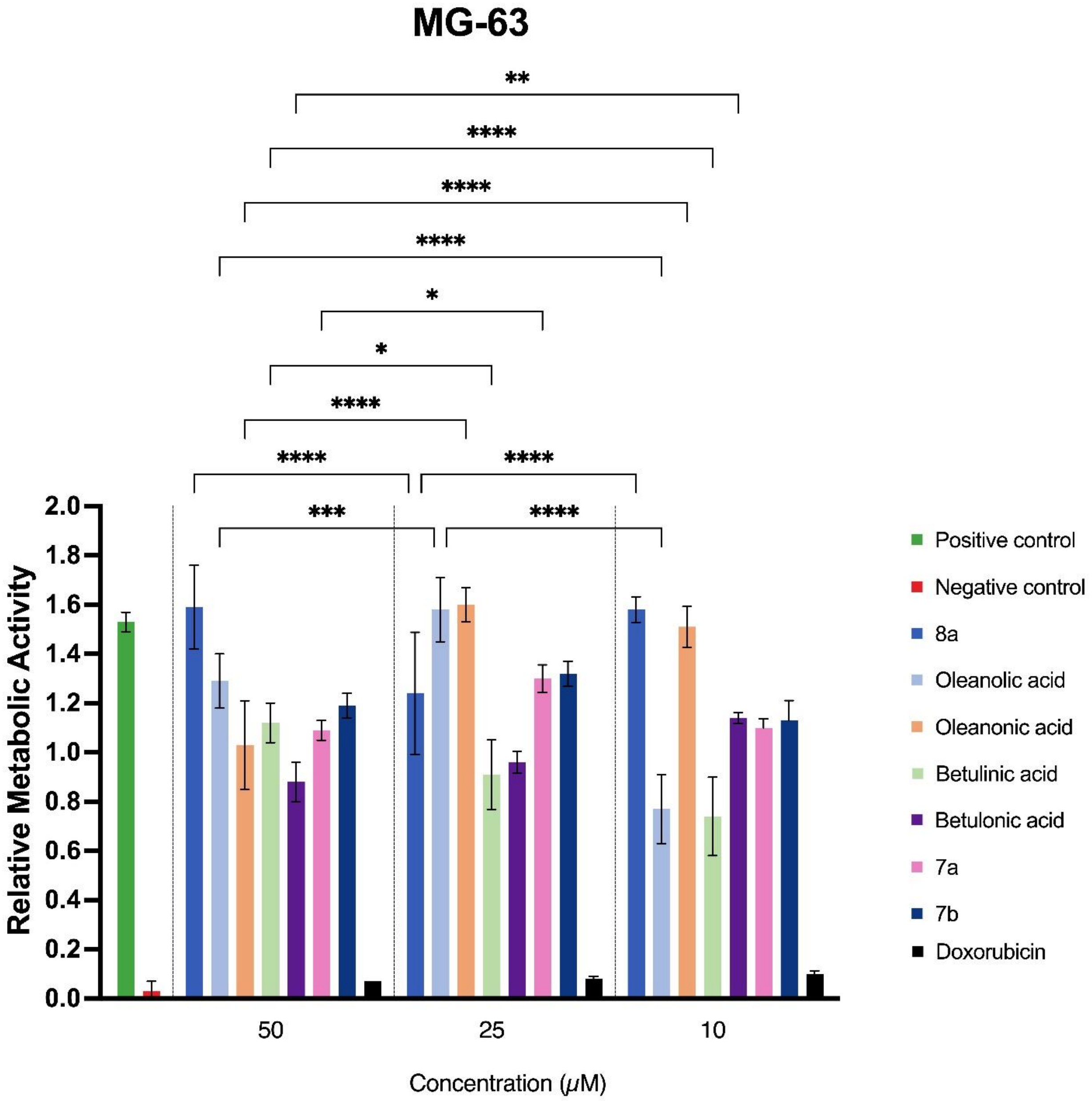

Figure S1. Relative metabolic activity of MG-63 cells in the presence of **7a,b** and **8a** at various concentrations and their comparison with their relative metabolic activity in the presence of oleanolic, oleanonic and betulinic, betulonic acids, and doxorubicin. Statistically significant differences: * at $p < 0.05$; ** at $p < 0.005$; *** at $p < 0.001$; **** at $p < 0.0001$

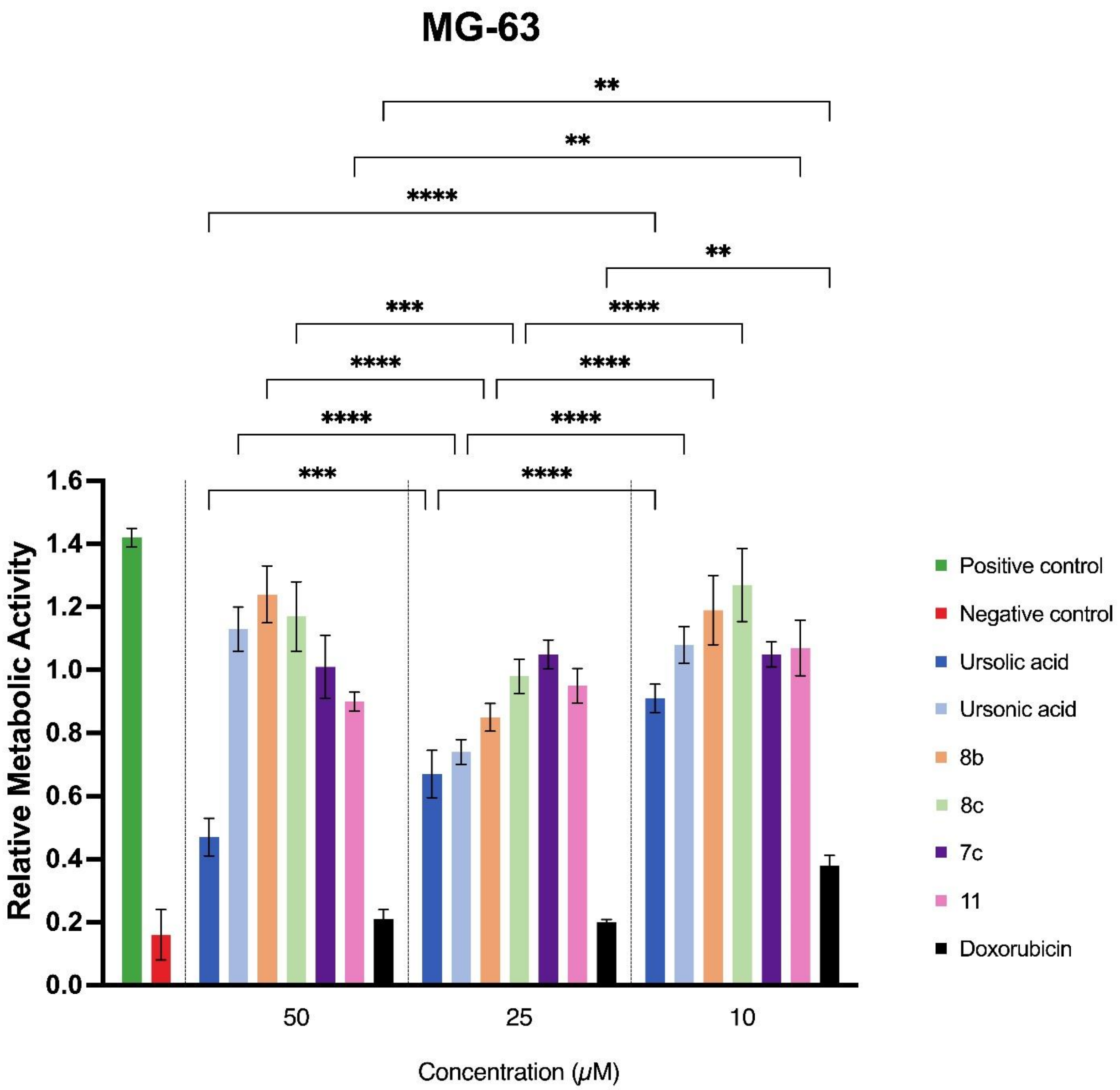

Figure S2. Relative metabolic activity of MG-63 cells in the presence of **7c, 8b,c** and **12** at various concentrations and their comparison with their relative metabolic activity in the presence of ursolic and ursonic acids, and doxorubicin. Statistically significant differences: * at $p < 0.05$; ** at $p < 0.005$; *** at $p < 0.001$; **** at $p < 0.0001$

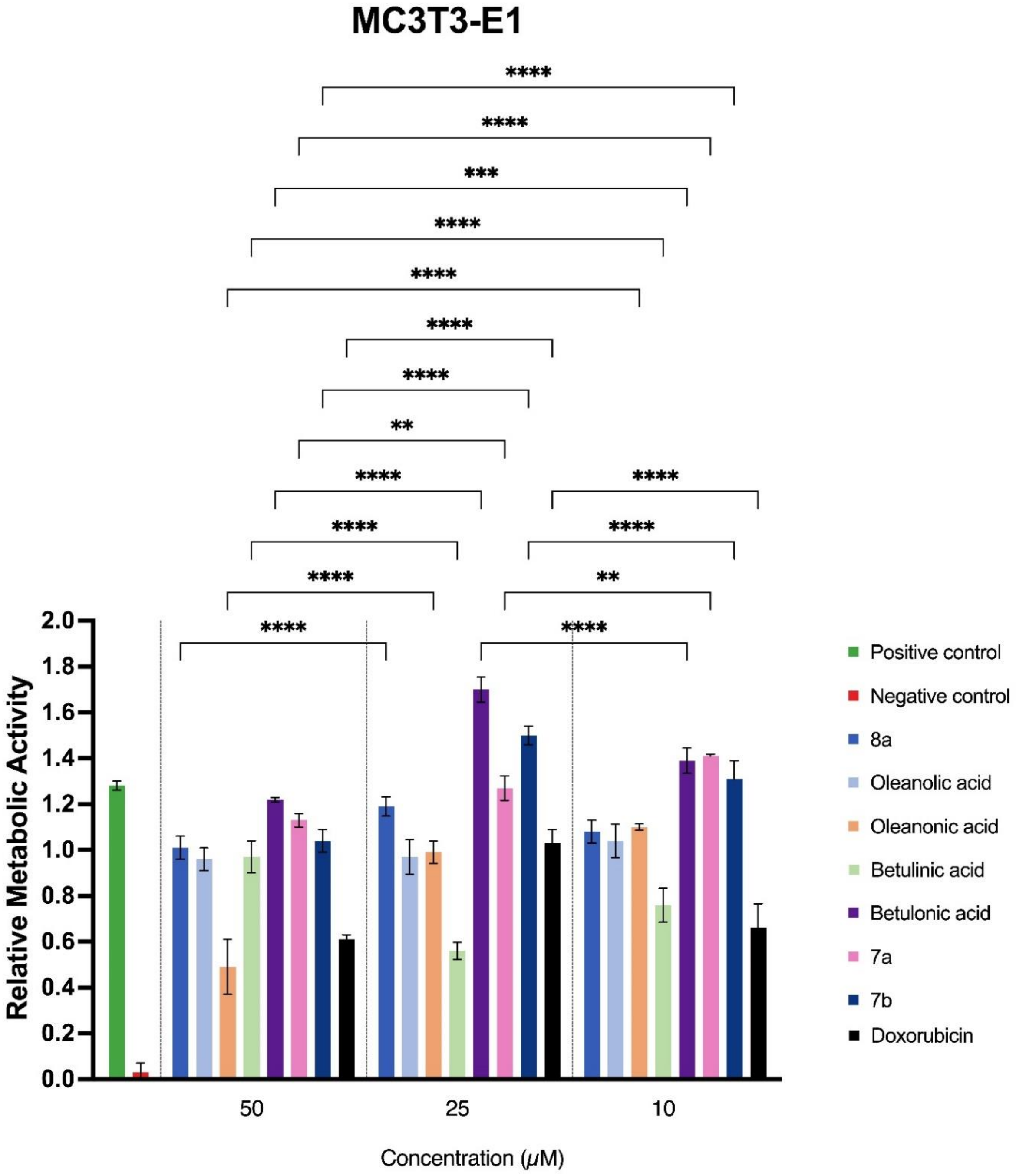

Figure S3. Relative metabolic activity of MC3T3-E1 cells in the presence of **7a,b** and **8a** at various concentrations and their comparison with their relative metabolic activity in the presence of oleanolic, oleanonic and betulinic, betulonic acids, and doxorubicin. Statistically significant differences: * at $p < 0.05$; ** at $p < 0.005$; *** at $p < 0.001$; **** at $p < 0.0001$

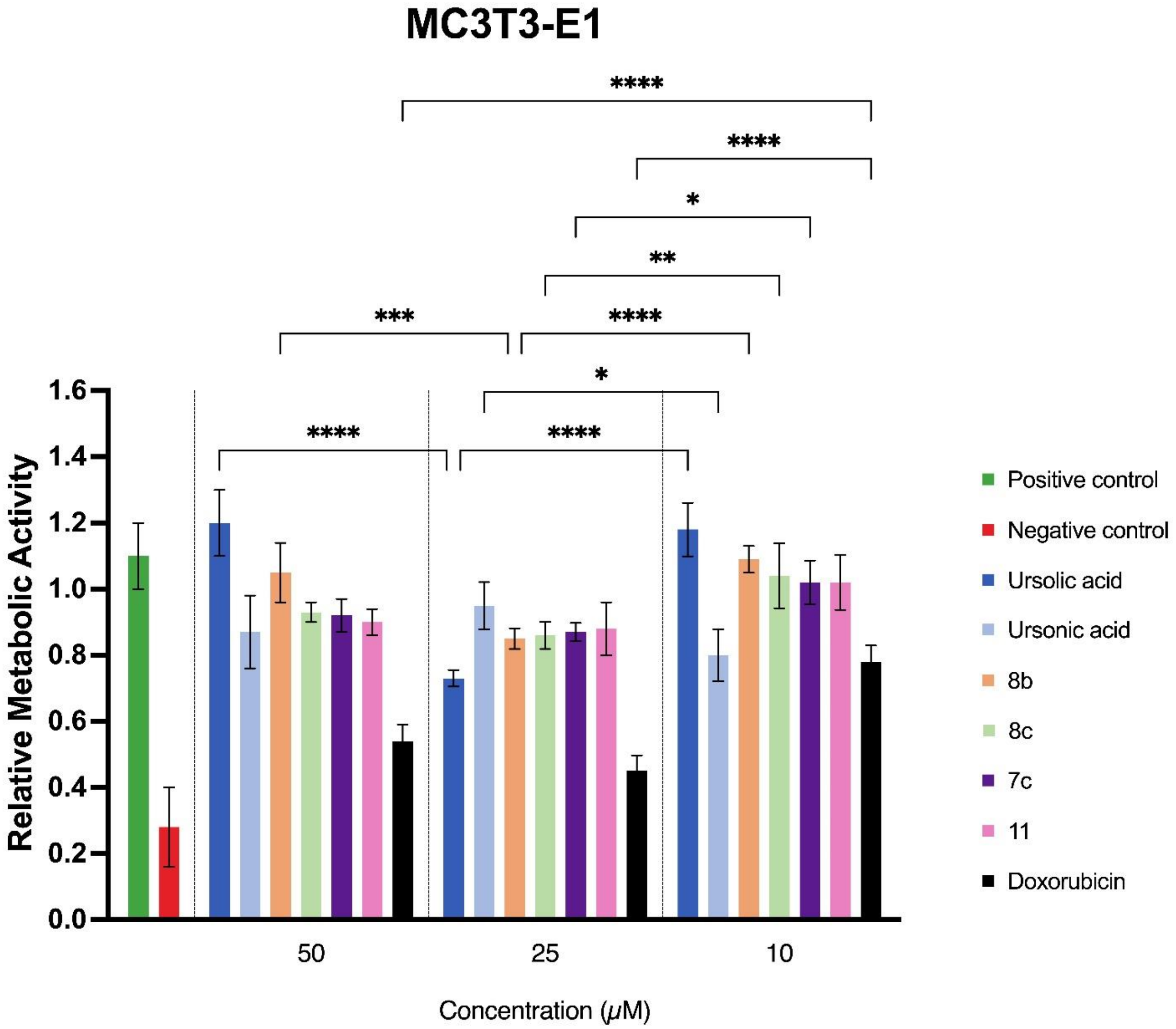

Figure S4. Relative metabolic activity of MC3T3-E1 cells in the presence of **7c, 8b,c** and **12** at various concentrations and their comparison with their relative metabolic activity in the presence of ursolic and ursonic acids, and doxorubicin. Statistically significant differences: * at $p < 0.05$; ** at $p < 0.005$; *** at $p < 0.001$; **** at $p < 0.0001$
